# Supplementary material for: The Uso1 globular head interacts with SNAREs to maintain viability even in the absence of the coiled-coil domain
Source: eLife. 2023 May 30;12:e85079. doi: 10.7554/eLife.85079 (PMC10275640; doi:10.7554/eLife.85079)
Supplement: Figure 1—source data 1. [file elife-85079-fig1-data1.docx]

| Strain | 30ºC | 37ºC | 42ºC | Gene | nt change | AA change |
| --- | --- | --- | --- | --- | --- | --- |
| *rab1 wt* | + | + | + | *rab1* | - | A136 |
| *rab1 ts* | + | - | - | *rab1* | C407A; A408C | A136D |
| Intragenic suppressors | | | | | | |
| *su2* | + | + | + | *rab1* | C408A | D136E |
| *su4* | + | + | + | *rab1* | C408A | D136E |
| *su15* | + | + | + | *rab1* | A407T; C408T | D136V |
| *su70* | + | + | + | *rab1* | G406A | D136N |
| Extragenic suppressors | | | | | | |
| *su1* | + | + | - | *uso1* | G16A | E6K |
| *su11* | + | + | - | *uso1* | G16A | E6K |
| *su16* | + | + | - | *uso1* | G16A | E6K |
| *su19* | + | + | - | *uso1* | G16A | E6K |
| *su23* | + | + | - | *uso1* | G16A | E6K |
| *su31* | + | + | - | *uso1* | G16A | E6K |
| *su40* | + | + | - | *uso1* | G16A | E6K |
| *su63* | + | + | - | *uso1* | G16A | E6K |
| *su85* | + | + | + | *uso1* | G1617A; G1618A | G540S |
| *su114* | + | + | - | *uso1* | G16A | E6K |
