# Supplementary material for: The Uso1 globular head interacts with SNAREs to maintain viability even in the absence of the coiled-coil domain
Source: eLife. 2023 May 30;12:e85079. doi: 10.7554/eLife.85079 (PMC10275640; doi:10.7554/eLife.85079)
Supplement: Figure 3—source data 1. [file elife-85079-fig3-data1.docx]

| Protein | Genotype | *s_exp_*  (x10^-13^ s) | *D_exp_*  (x10^-11^ m^2^/s) | *M_r_* (kDa) | *M_1_*  (kDa) | *n* | Quaternary  structure |
| --- | --- | --- | --- | --- | --- | --- | --- |
| Uso1 | wild-type | 4.8 | 1.8 | 246 | 125 | 1.98 | dimer |
|  | E6K G540S | 4.7 | 1.7 | 255 | 125 | 2.05 | dimer |
| Uso1  ∆CTR | wild-type | 4.8 | 2.0 | 231 | 118 | 1.96 | dimer |
|  | E6K G540S | 4.9 | 2.1 | 224 | 118 | 1.91 | dimer |
| Uso1  GHD | wild-type | 3.7 | 5.2 | 68 | 74 | 0.93 | monomer |
|  | E6K G540S | 3.9 | 5.4 | 69 | 74 | 0.94 | monomer |
| Uso1  CCD | wild-type | 2.7 | 2.3 | 104 | 52 | 2.01 | dimer |
