# Supplementary figures and images for: The Uso1 globular head interacts with SNAREs to maintain viability even in the absence of the coiled-coil domain
Source: eLife. 2023 May 30;12:e85079. doi: 10.7554/eLife.85079 (PMC10275640; doi:10.7554/eLife.85079)

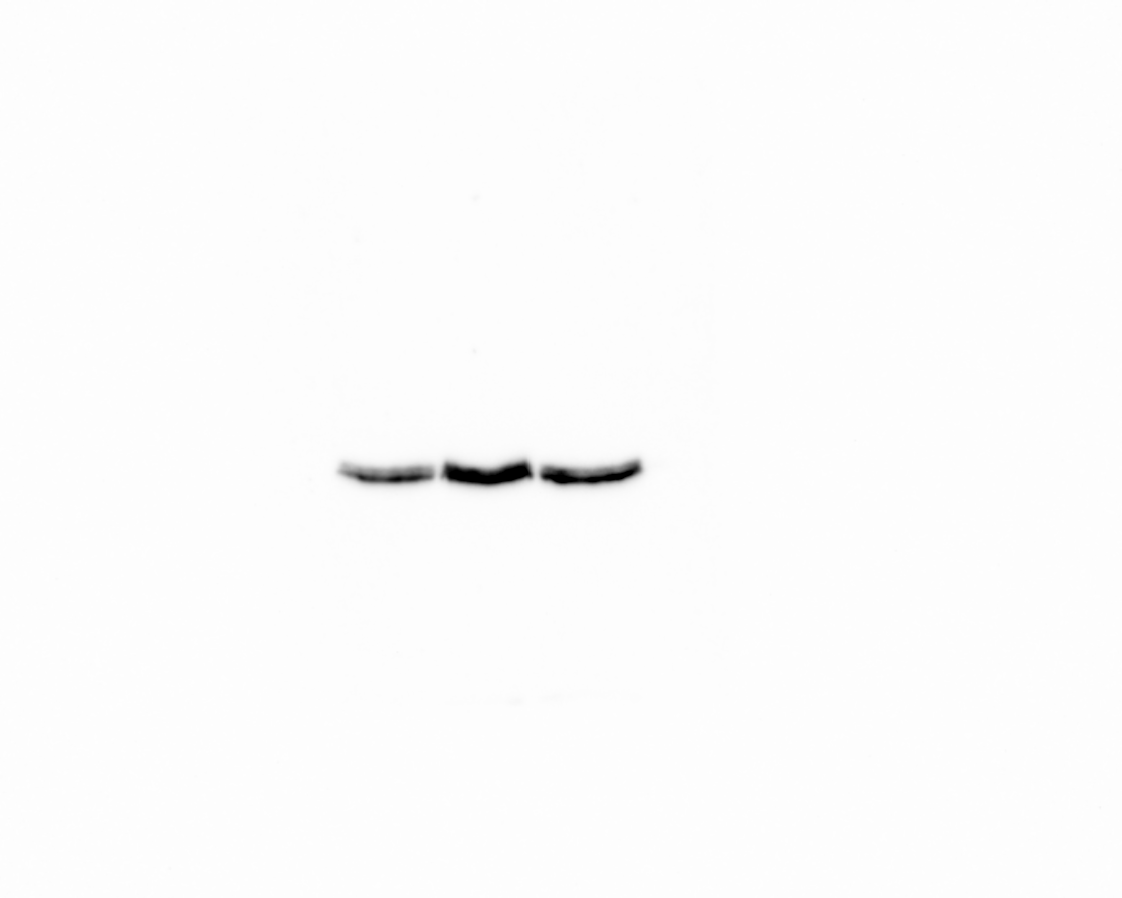

Supplement: Figure 8—source data 1. [file elife-85079-fig8-data1.zip › FIGURE 8 Source data/RAW data/Figure 8. panel D. WB anti-tubuline.tif]

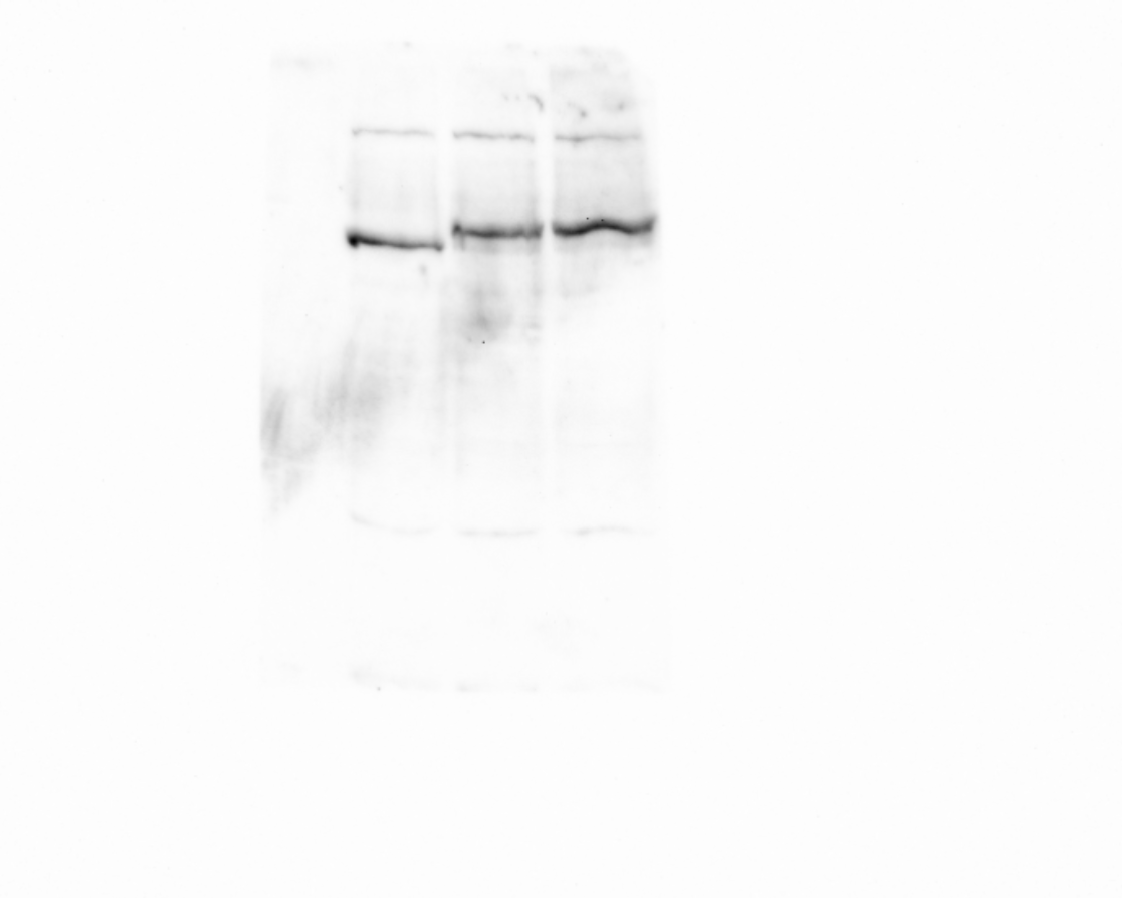

Supplement: Figure 8—source data 1. [file elife-85079-fig8-data1.zip › FIGURE 8 Source data/RAW data/Figure 8. panel D. WB anti-Uso1 GHD.tif]

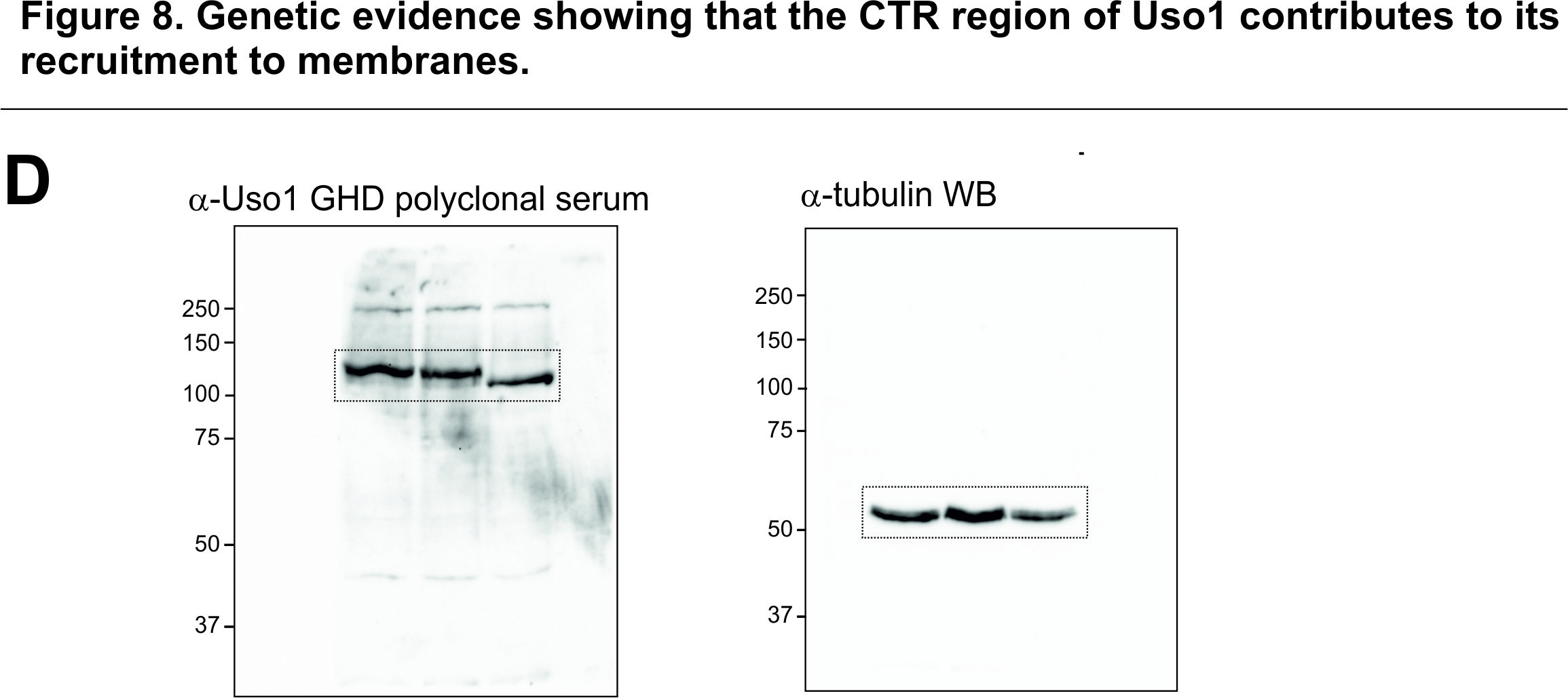

Supplement: Figure 8—source data 1. [file elife-85079-fig8-data1.zip › FIGURE 8 Source data/uncropped blots Figure 8.jpg]

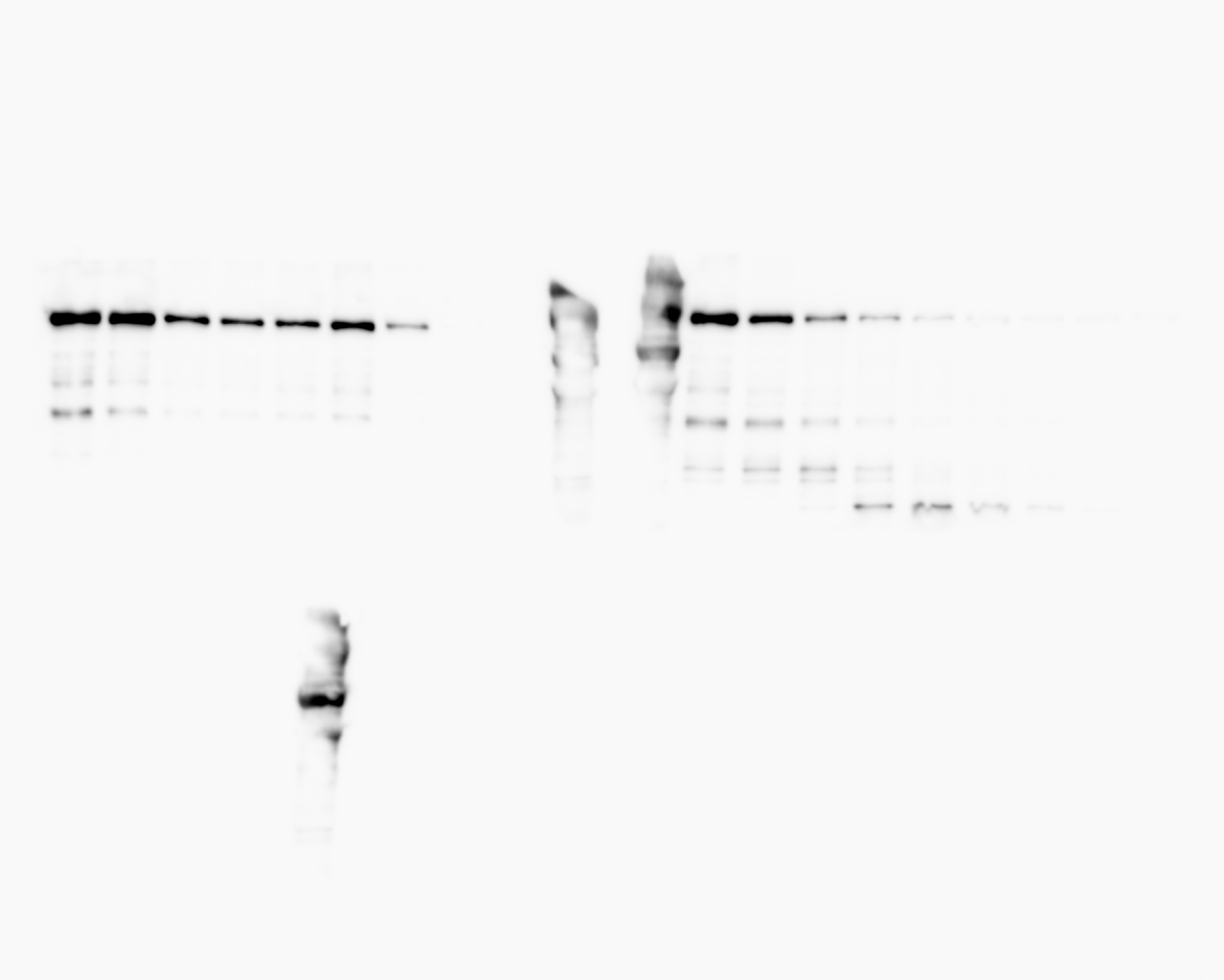

Supplement: Figure 9—source data 1. [file elife-85079-fig9-data1.zip › FIGURE 9 Source data/RAW data Fig 9/Fig. 09 panel B/Figure 9. panel B. Uso1 full length-His. WB anti-His.tif]

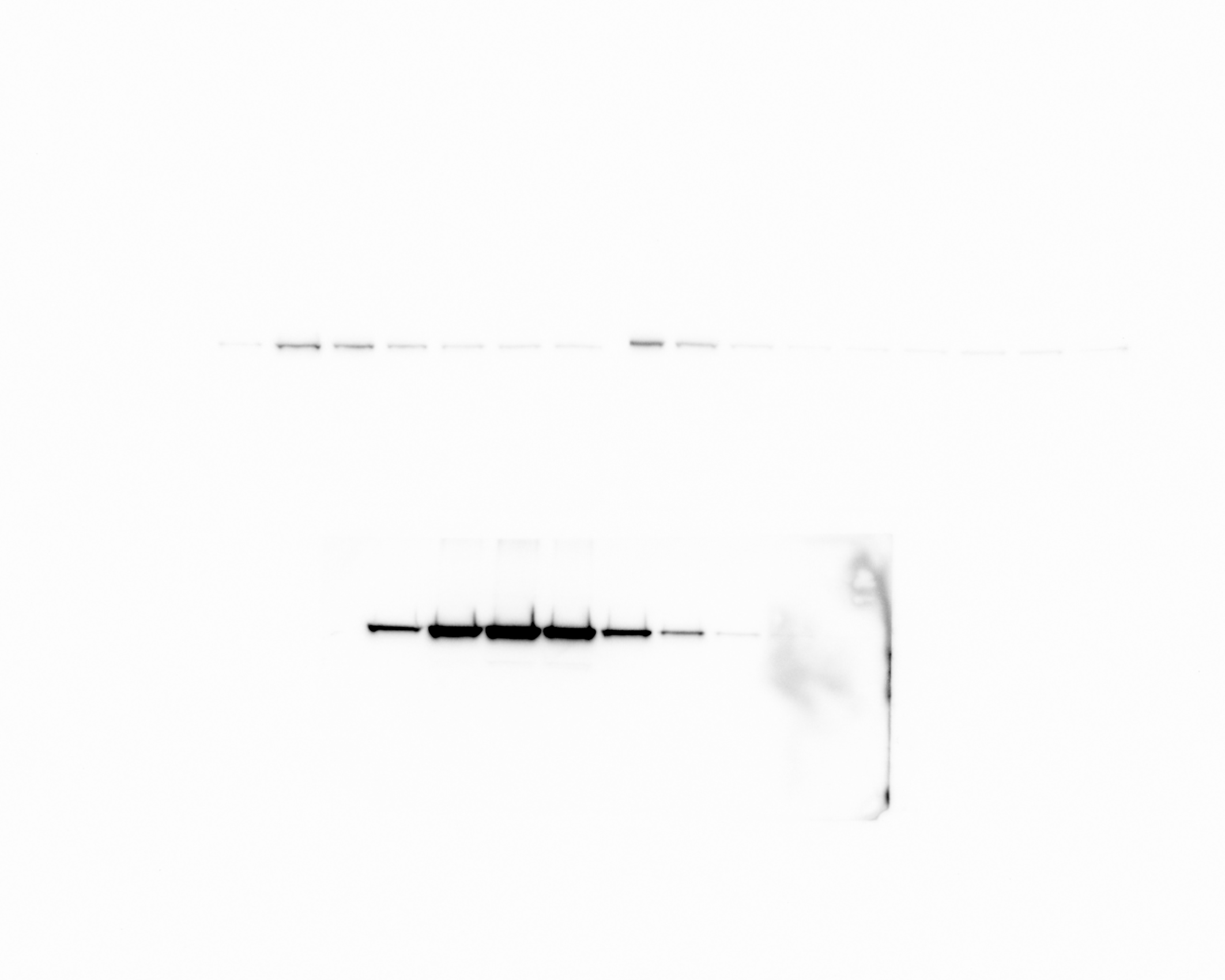

Supplement: Figure 9—source data 1. [file elife-85079-fig9-data1.zip › FIGURE 9 Source data/RAW data Fig 9/Fig. 09 panel B/Figure 9. panel B. Uso1 GHD-HA. WB anti-HA.tif]

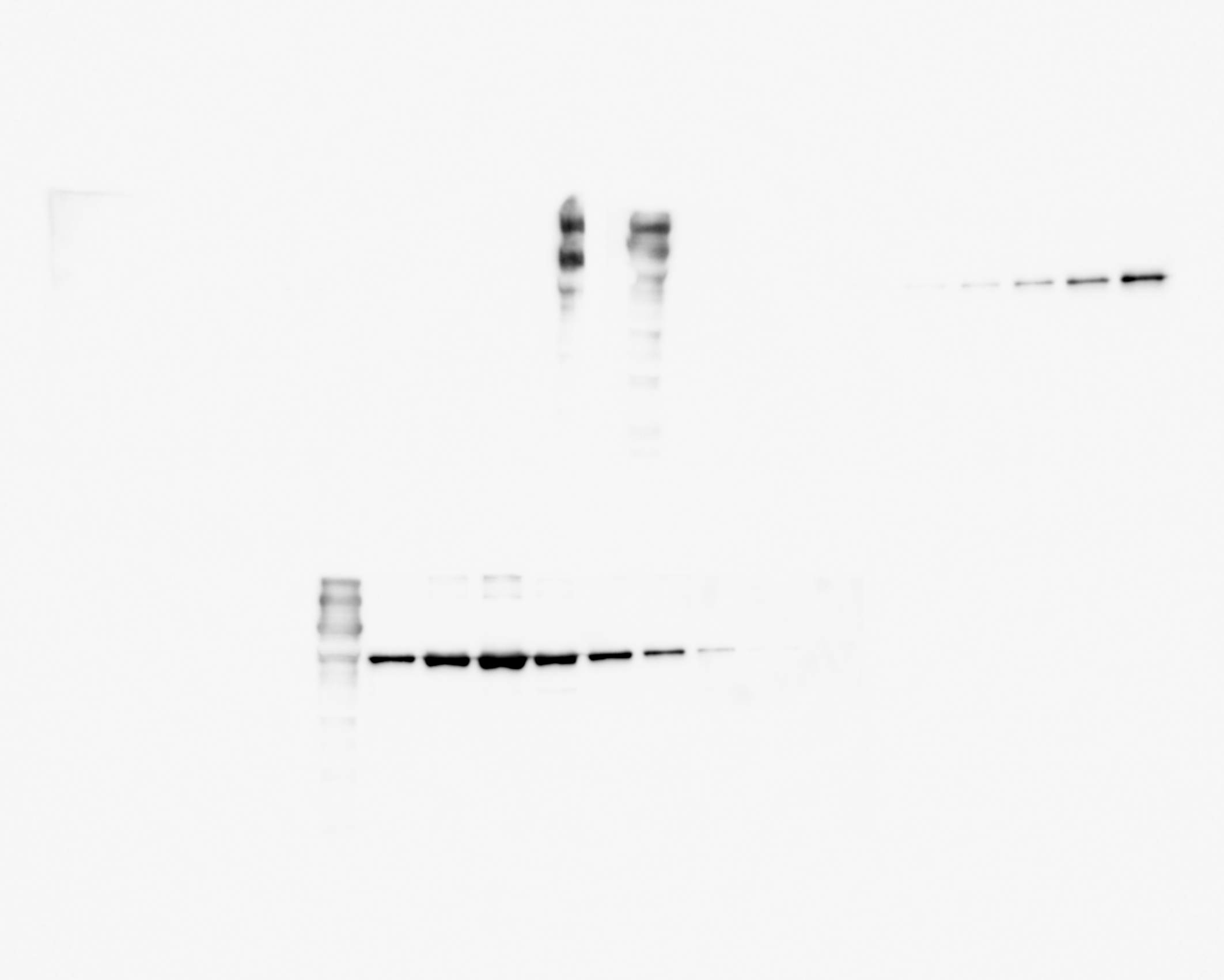

Supplement: Figure 9—source data 1. [file elife-85079-fig9-data1.zip › FIGURE 9 Source data/RAW data Fig 9/Fig. 09 panel B/Figure 9. panel B. Uso1 GHD-His. WB anti-His.tif]

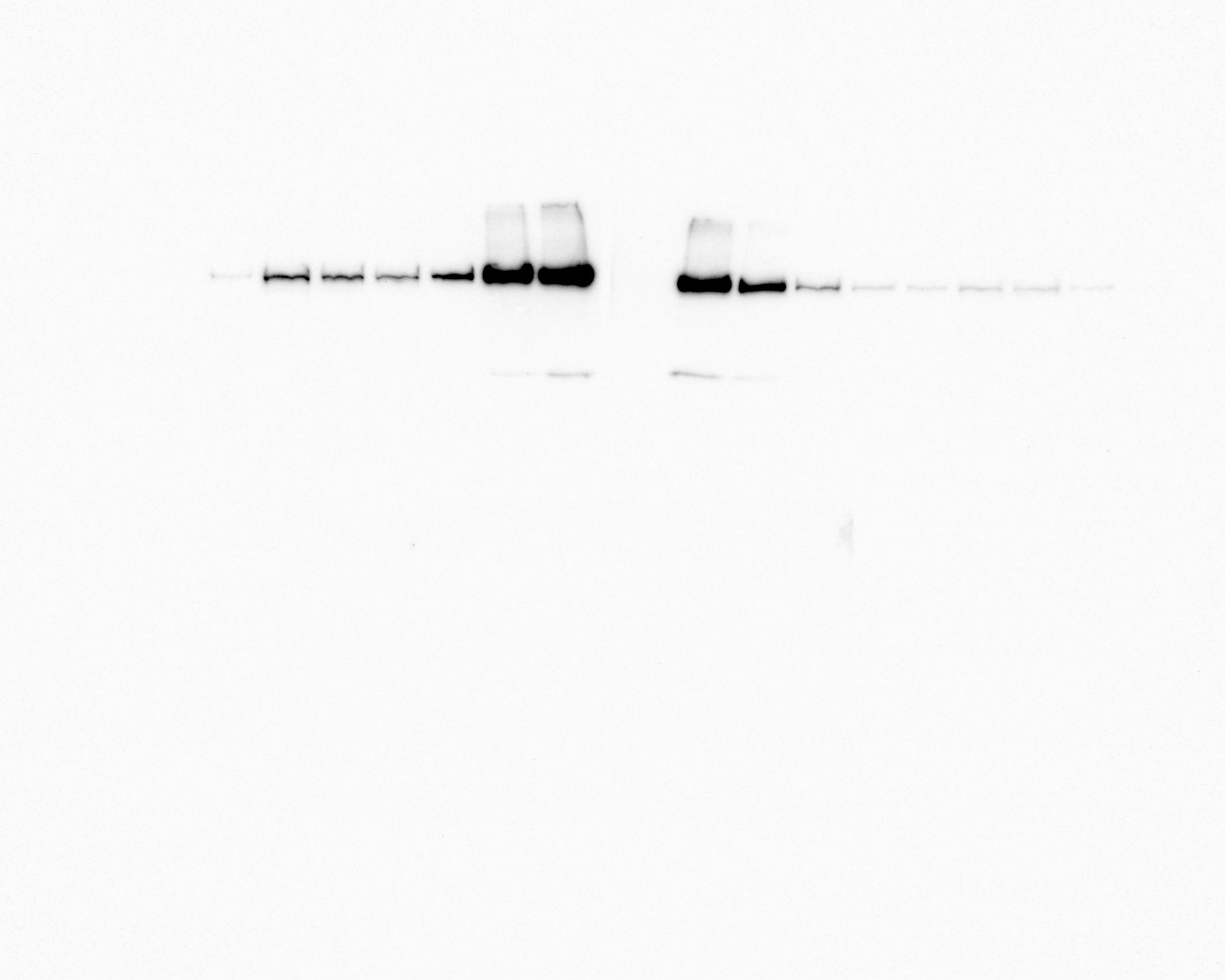

Supplement: Figure 9—source data 1. [file elife-85079-fig9-data1.zip › FIGURE 9 Source data/RAW data Fig 9/Fig. 09 panel B/Figure 9. panel B. Uso1-HA. WB anti-HA.tif]

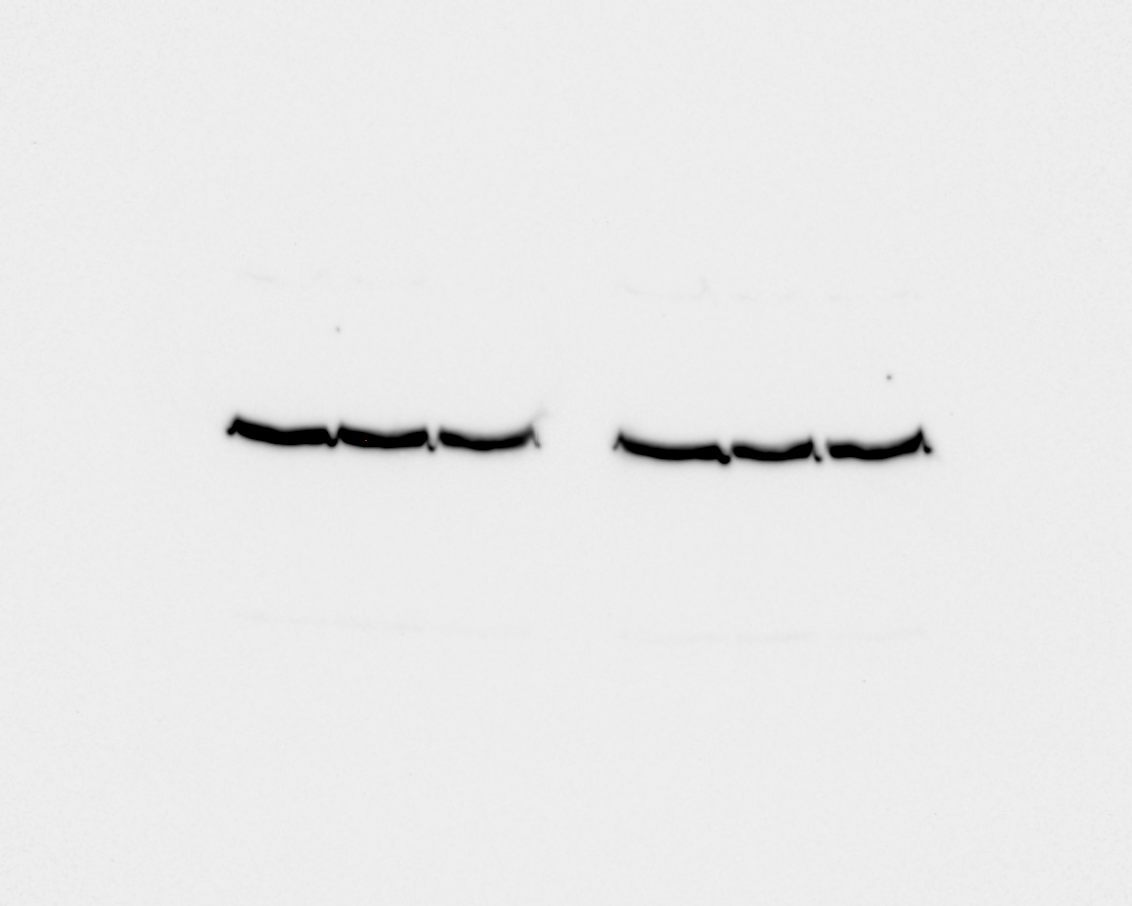

Supplement: Figure 9—source data 1. [file elife-85079-fig9-data1.zip › FIGURE 9 Source data/RAW data Fig 9/Fig. 09 panel C/Figure 9. panel C WB anti-tubulin.tif]

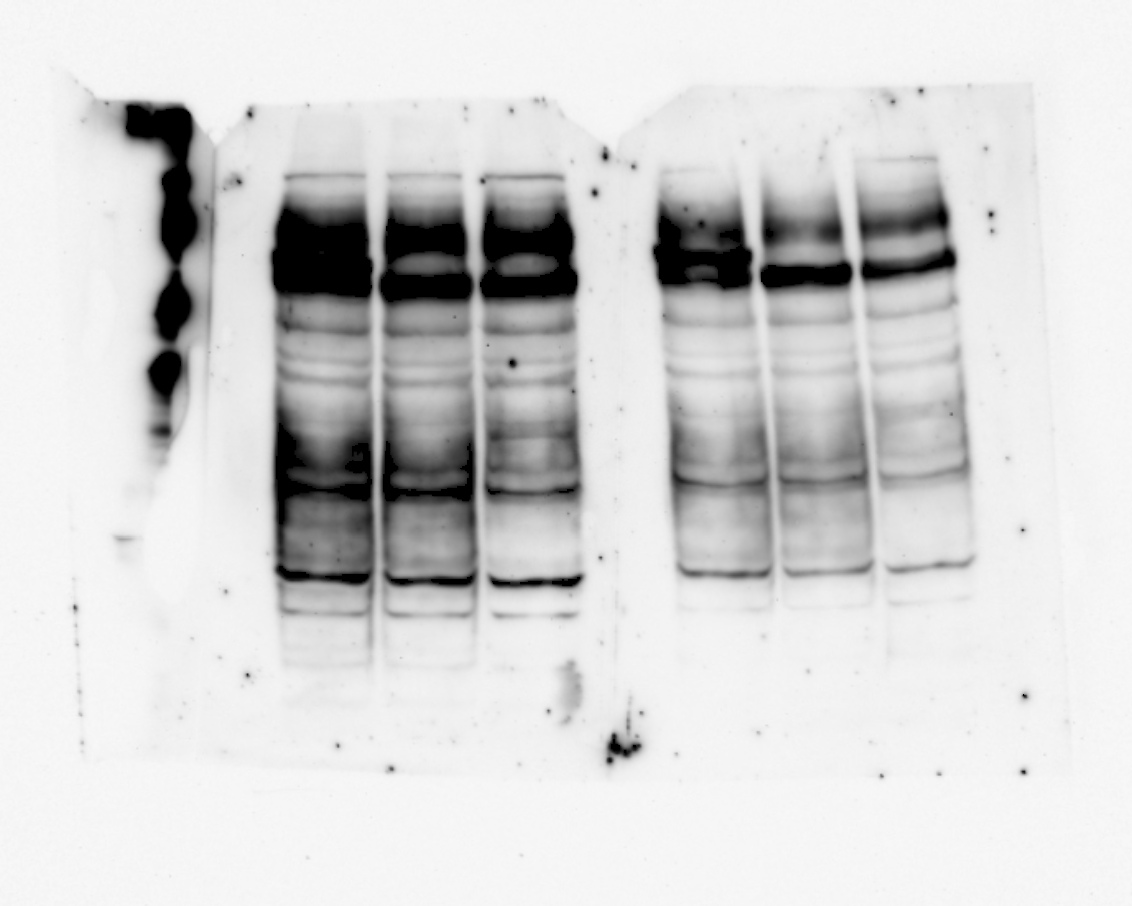

Supplement: Figure 9—source data 1. [file elife-85079-fig9-data1.zip › FIGURE 9 Source data/RAW data Fig 9/Fig. 09 panel C/Figure 9. panel C WB anti-Uso1 GHD (+ exp).tif]

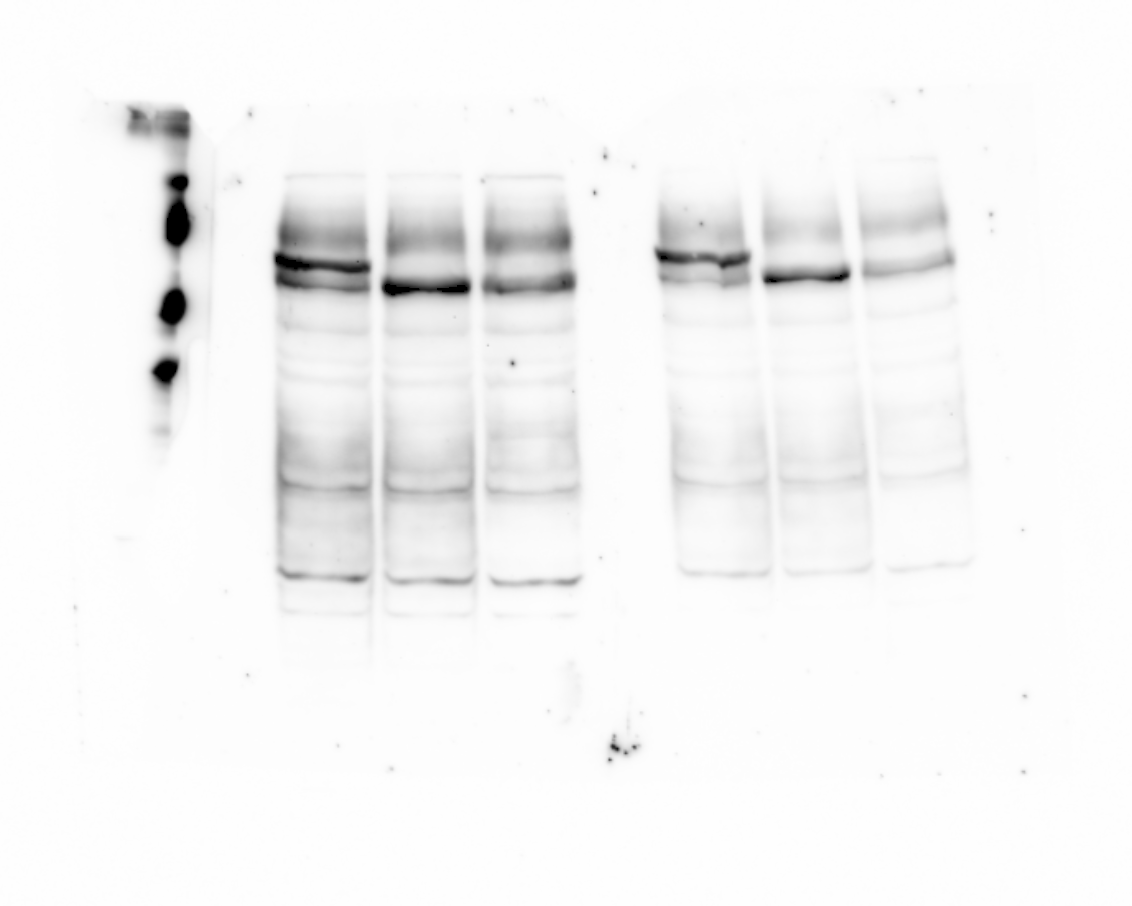

Supplement: Figure 9—source data 1. [file elife-85079-fig9-data1.zip › FIGURE 9 Source data/RAW data Fig 9/Fig. 09 panel C/Figure 9. panel C. WB anti-Uso1 GHD.tif]

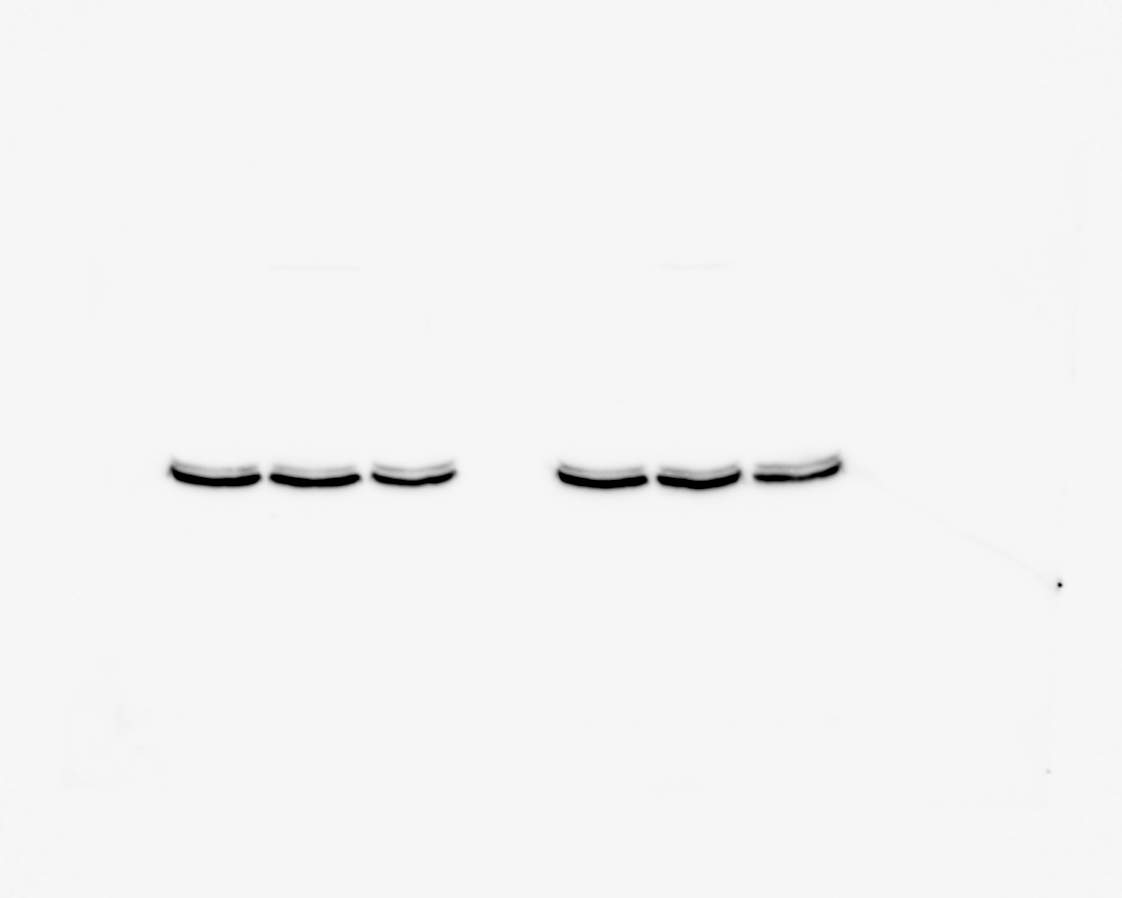

Supplement: Figure 9—source data 1. [file elife-85079-fig9-data1.zip › FIGURE 9 Source data/RAW data Fig 9/Fig. 09 panel D/Figure 9. panel D. WB anti-tubulin.tif]

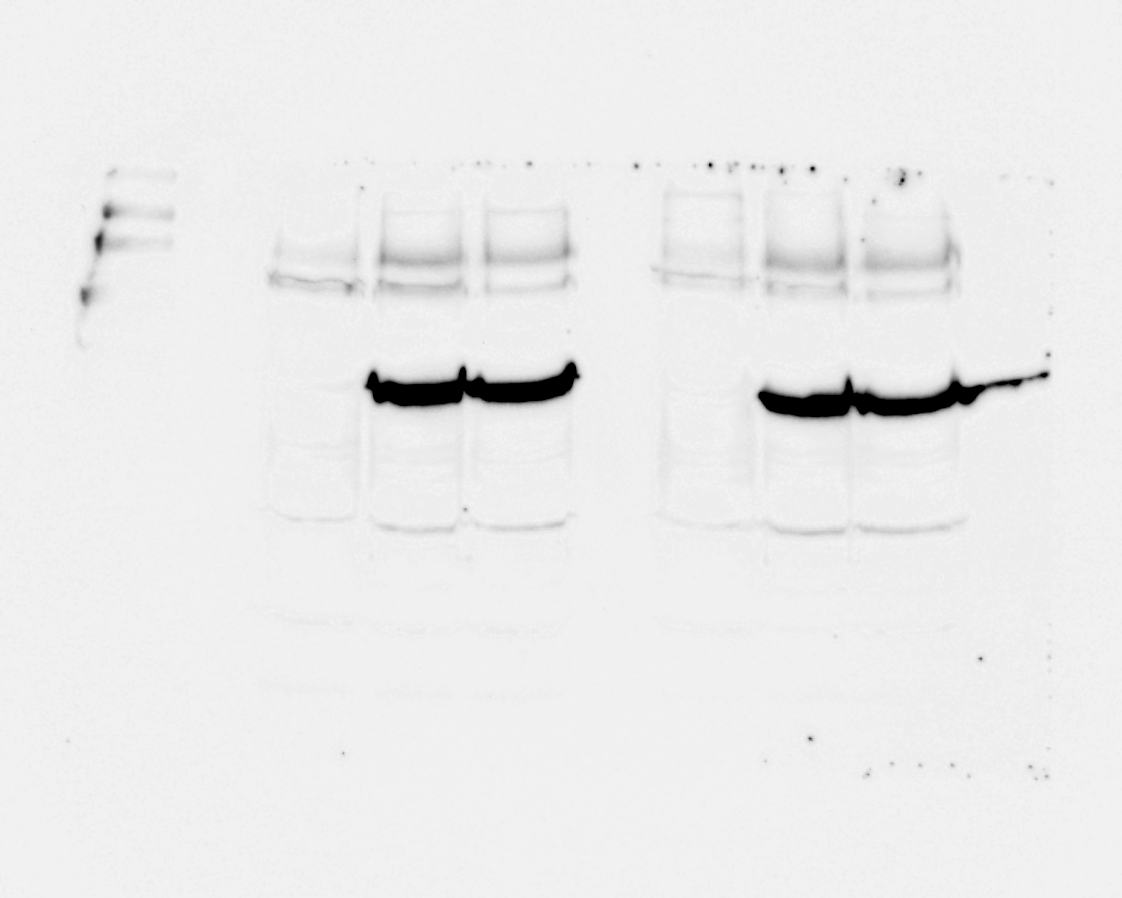

Supplement: Figure 9—source data 1. [file elife-85079-fig9-data1.zip › FIGURE 9 Source data/RAW data Fig 9/Fig. 09 panel D/Figure 9. panel D. WB anti-Uso1 GHD 2.5.tif]

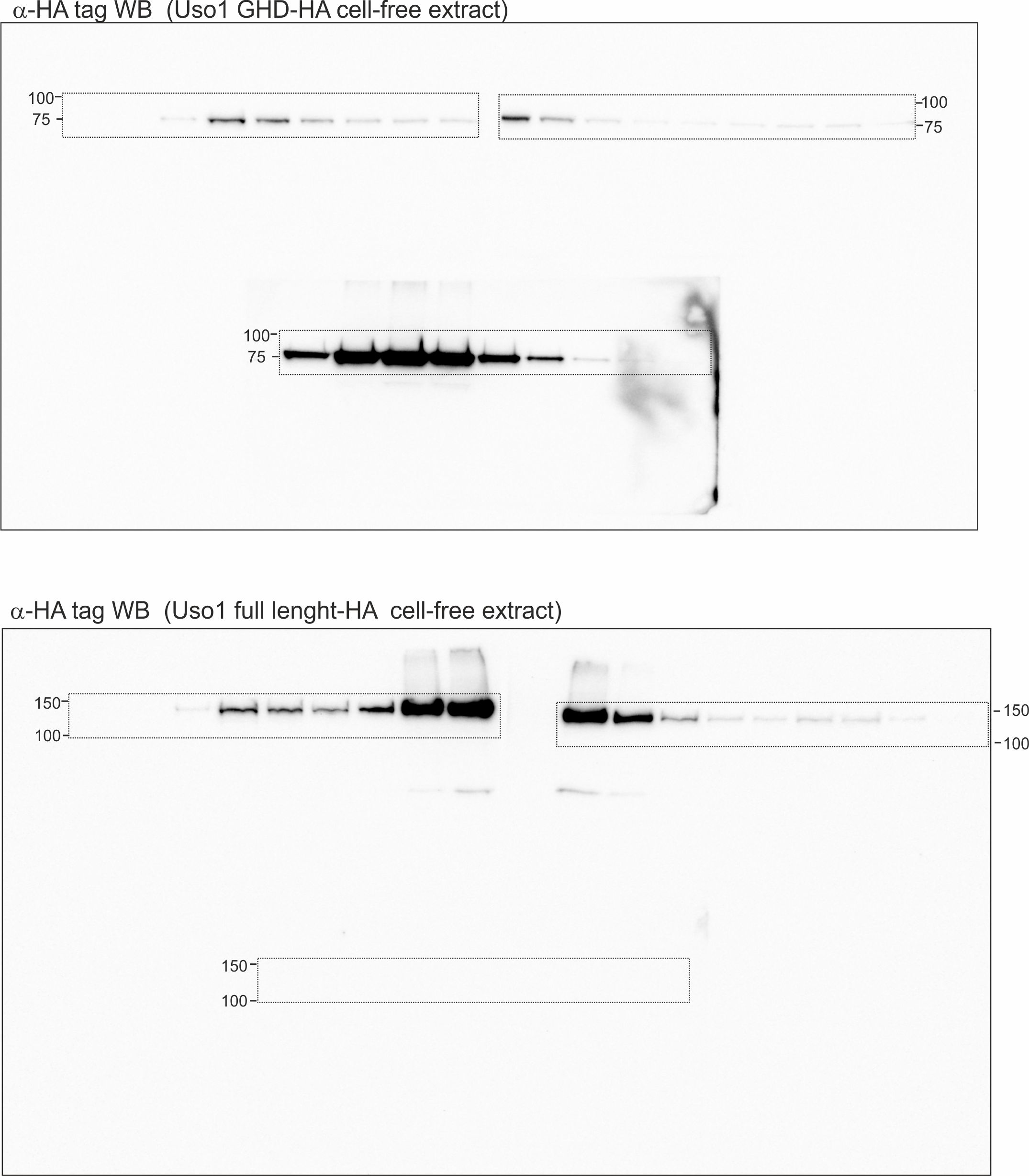

Supplement: Figure 9—source data 1. [file elife-85079-fig9-data1.zip › FIGURE 9 Source data/uncropped anti HA WBs Fig 9B.jpg]

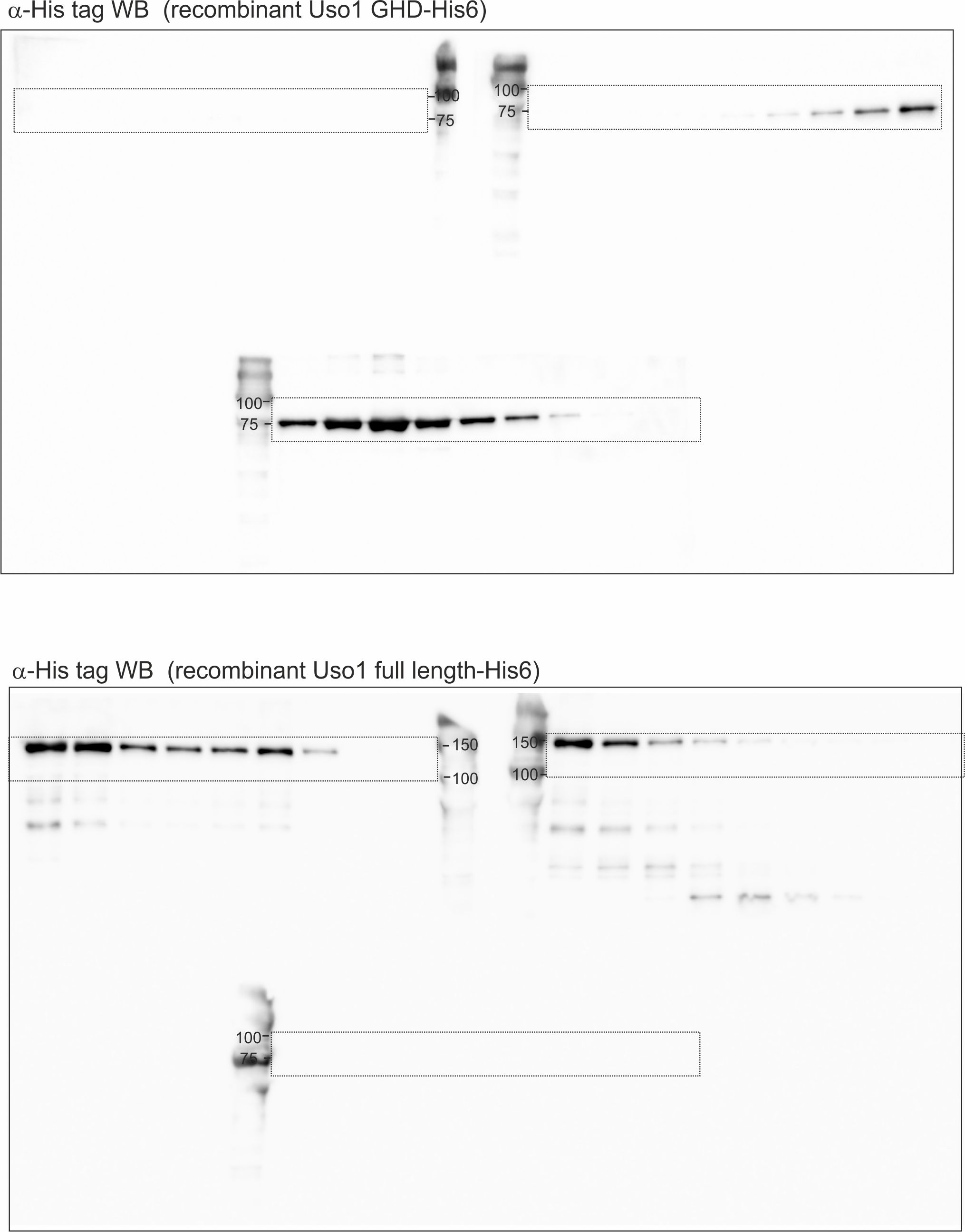

Supplement: Figure 9—source data 1. [file elife-85079-fig9-data1.zip › FIGURE 9 Source data/uncropped anti_His WBs Figure 9B.jpg]

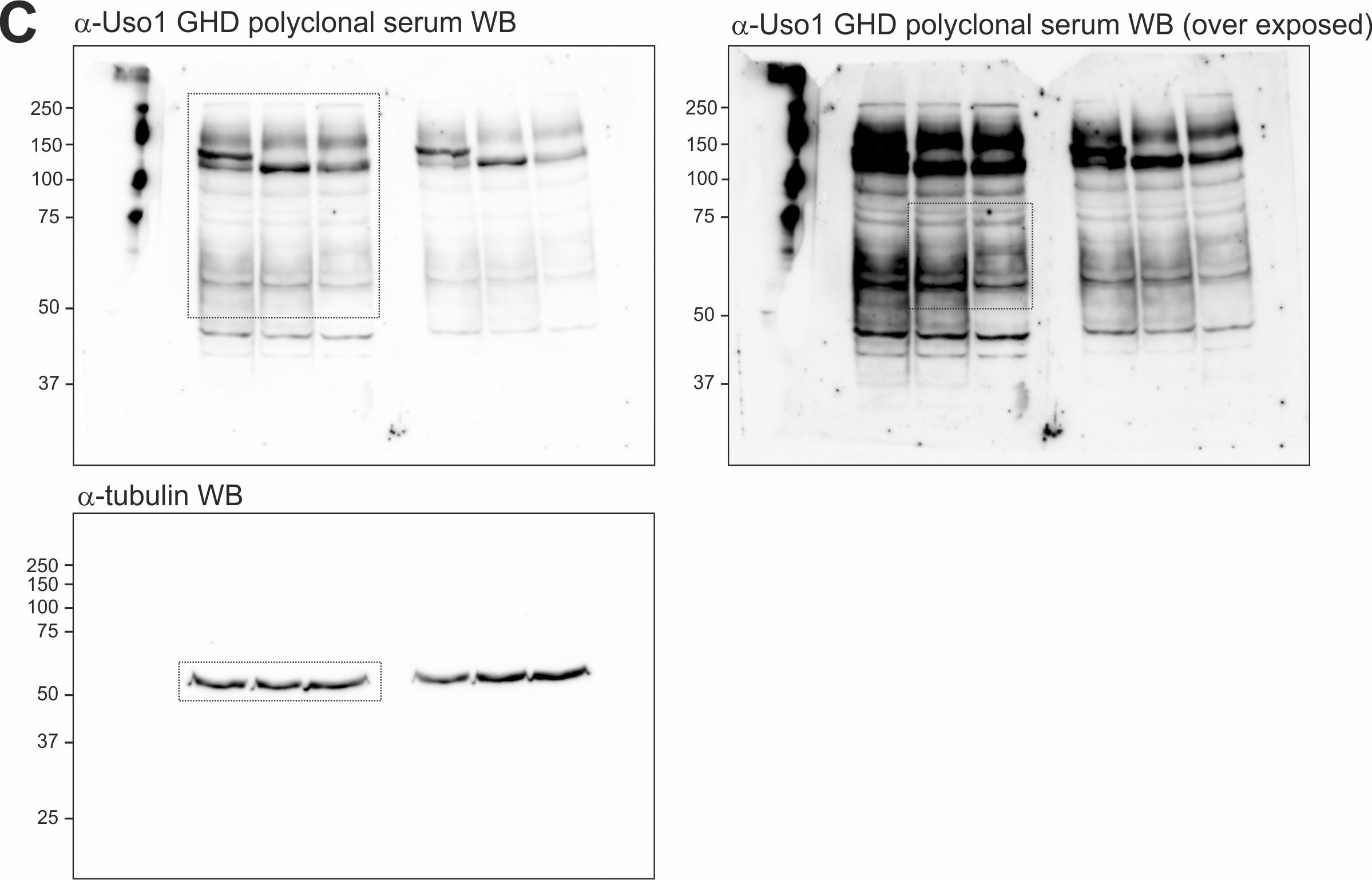

Supplement: Figure 9—source data 1. [file elife-85079-fig9-data1.zip › FIGURE 9 Source data/uncropped blots Fig 9C.jpg]

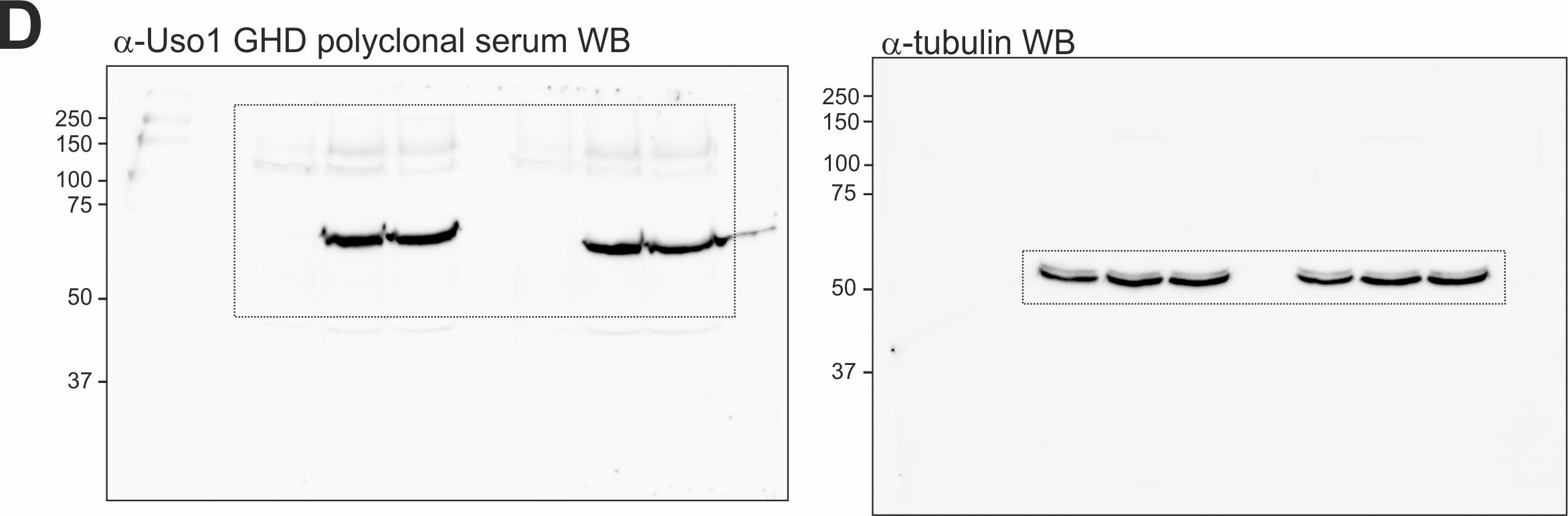

Supplement: Figure 9—source data 1. [file elife-85079-fig9-data1.zip › FIGURE 9 Source data/uncropped blots Figure 9D.jpg]

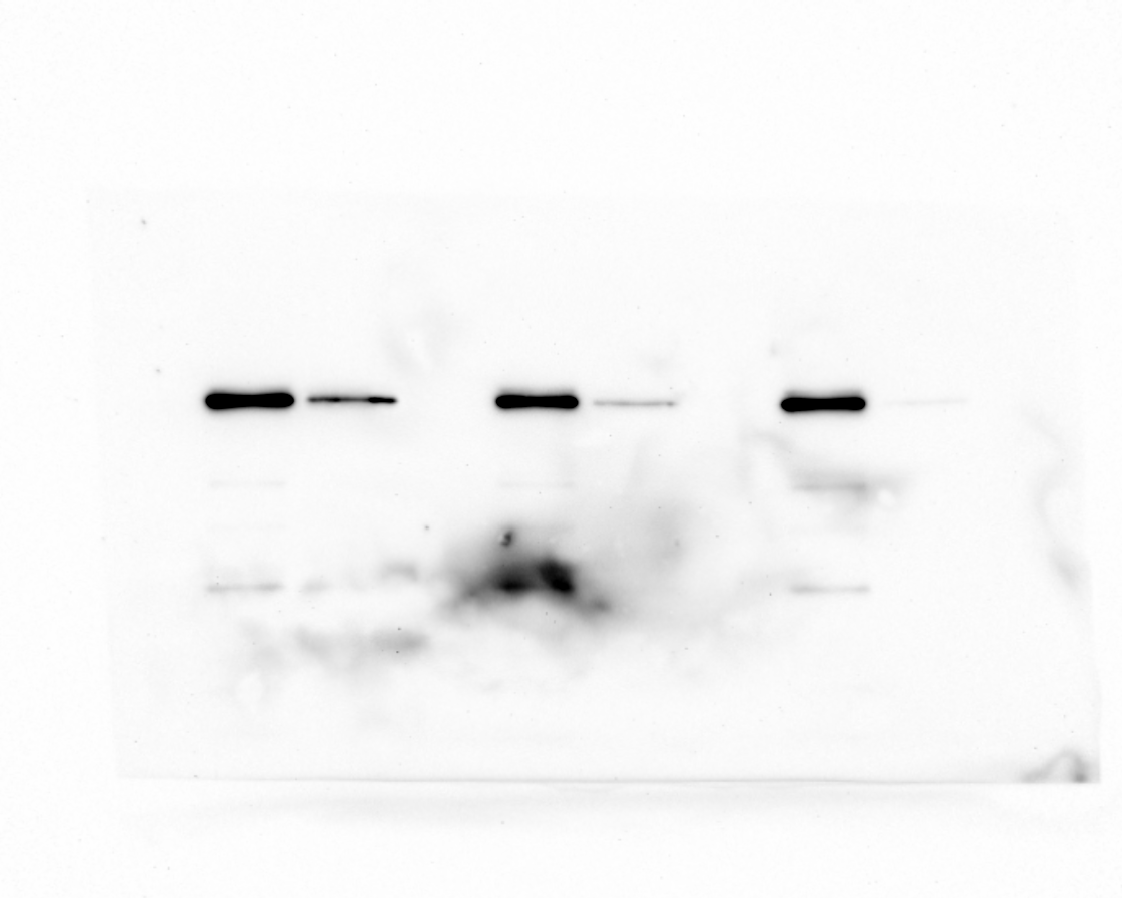

Supplement: Figure 10—source data 1. [file elife-85079-fig10-data1.zip › FIGURE 10 Source data/Fig. 10 raw data/1. Sed5-HA3 pull-down. anti-HA blot (PREY).tif]

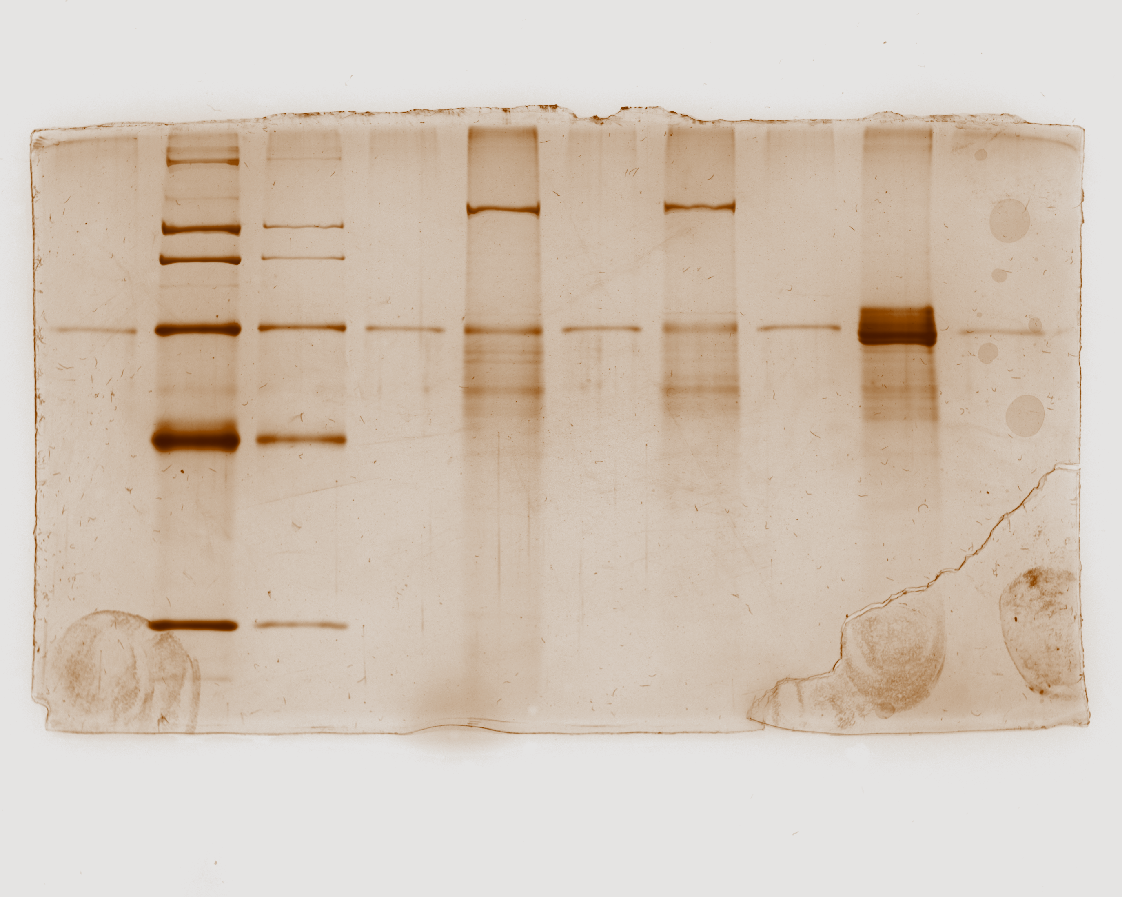

Supplement: Figure 10—source data 1. [file elife-85079-fig10-data1.zip › FIGURE 10 Source data/Fig. 10 raw data/1. Sed5-HA3 pull-down. silver stain gel (BAITS).tif]

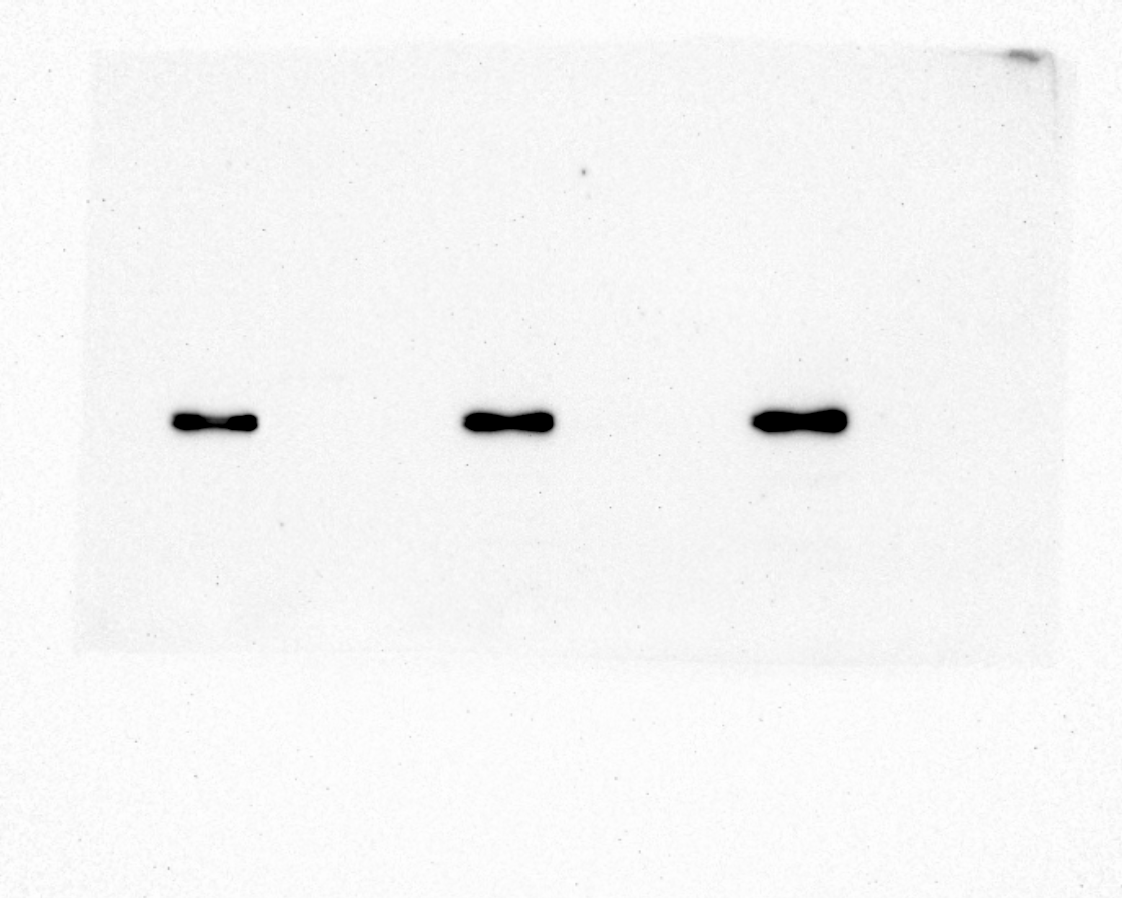

Supplement: Figure 10—source data 1. [file elife-85079-fig10-data1.zip › FIGURE 10 Source data/Fig. 10 raw data/10. Cog2-HA3 pull-down. anti-HA blot (PREY).tif]

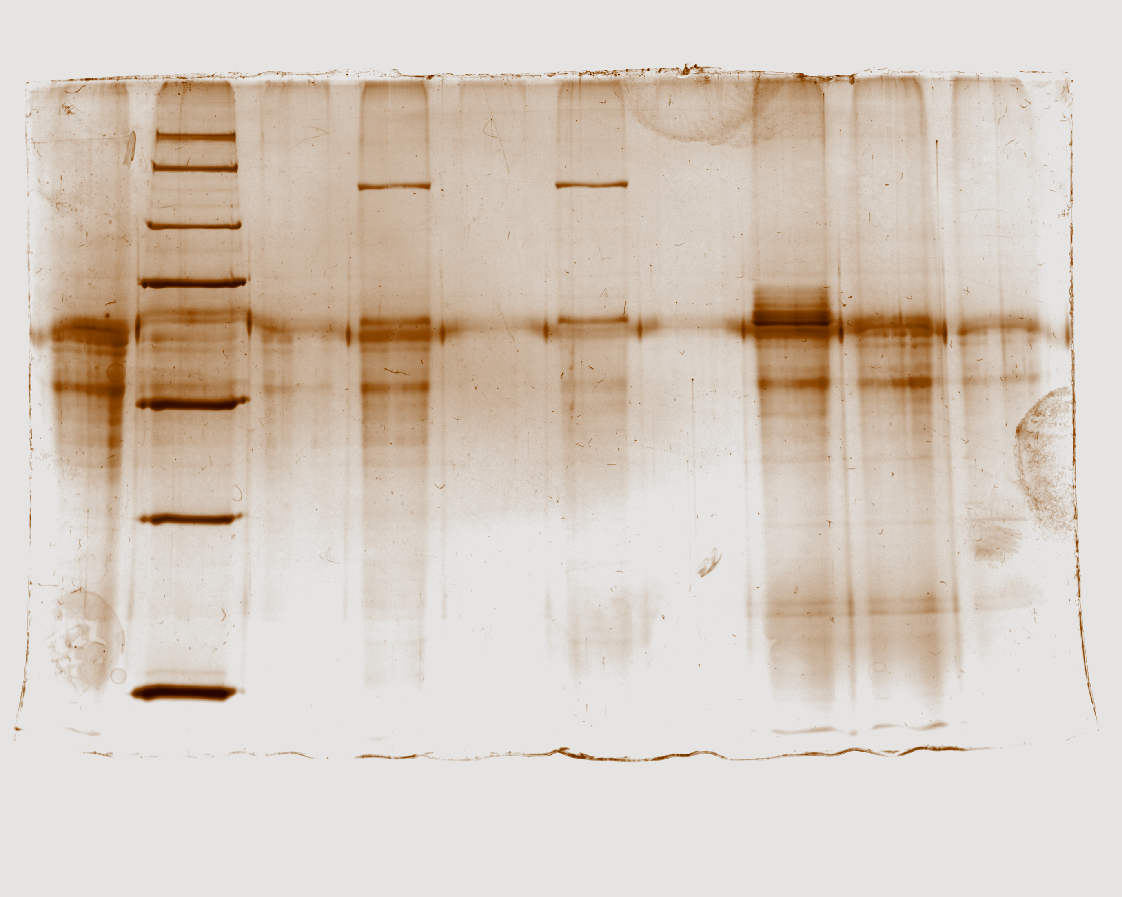

Supplement: Figure 10—source data 1. [file elife-85079-fig10-data1.zip › FIGURE 10 Source data/Fig. 10 raw data/10. Cog2-HA3 pull-down. silver stain gel (BAITS).tif]

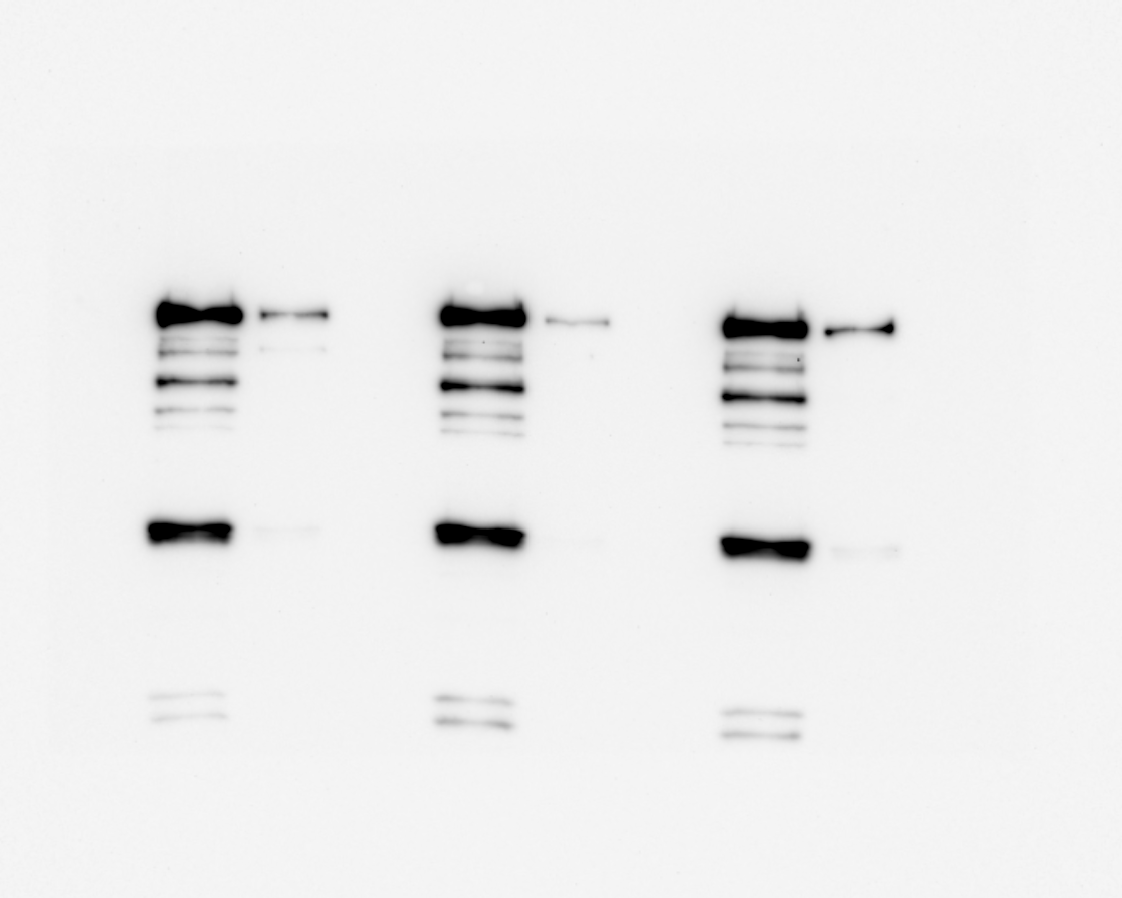

Supplement: Figure 10—source data 1. [file elife-85079-fig10-data1.zip › FIGURE 10 Source data/Fig. 10 raw data/11. ╬▓COP-HA3 pull-down. anti-HA blot (PREY).tif]

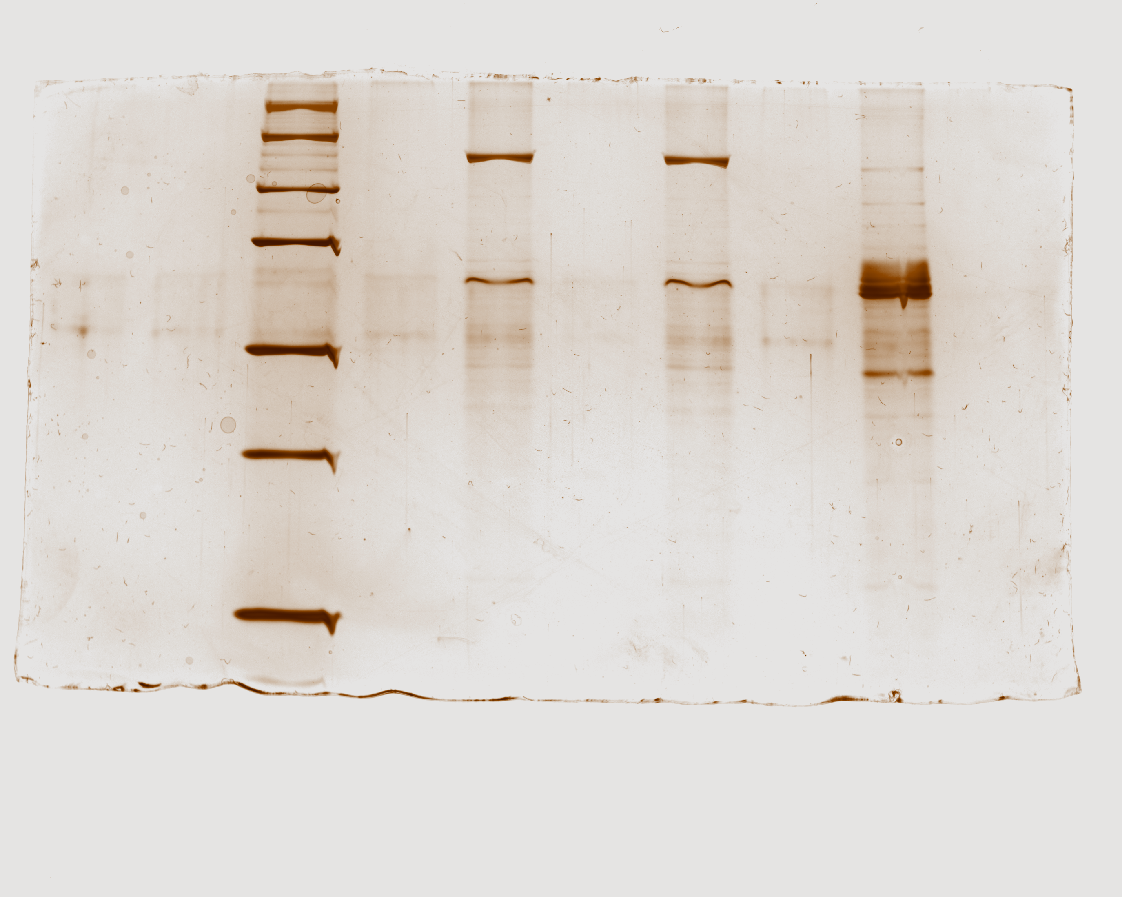

Supplement: Figure 10—source data 1. [file elife-85079-fig10-data1.zip › FIGURE 10 Source data/Fig. 10 raw data/11. ╬▓COP-HA3 pull-down. silver stain gel (BAITS).tif]

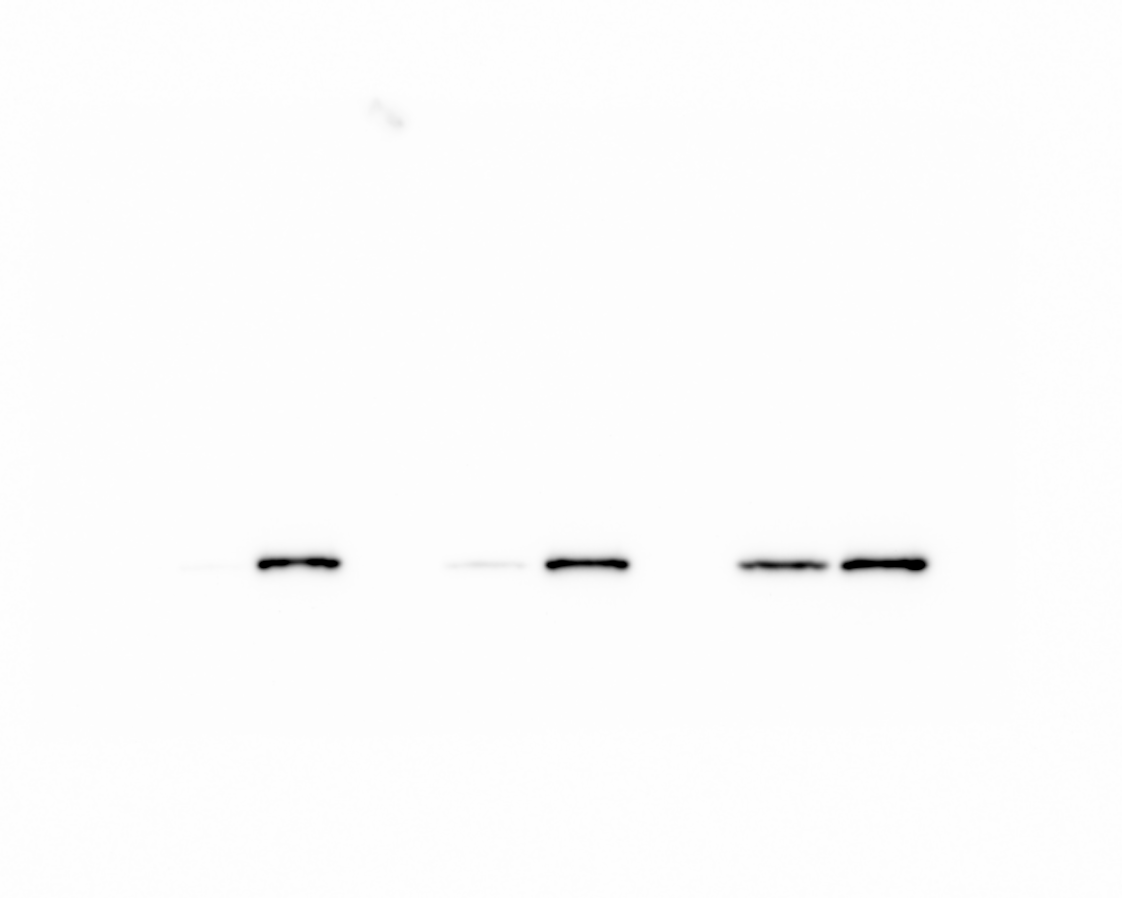

Supplement: Figure 10—source data 1. [file elife-85079-fig10-data1.zip › FIGURE 10 Source data/Fig. 10 raw data/3. Bos1-HA3 pull-down. anti-HA blot (PREY).tif]

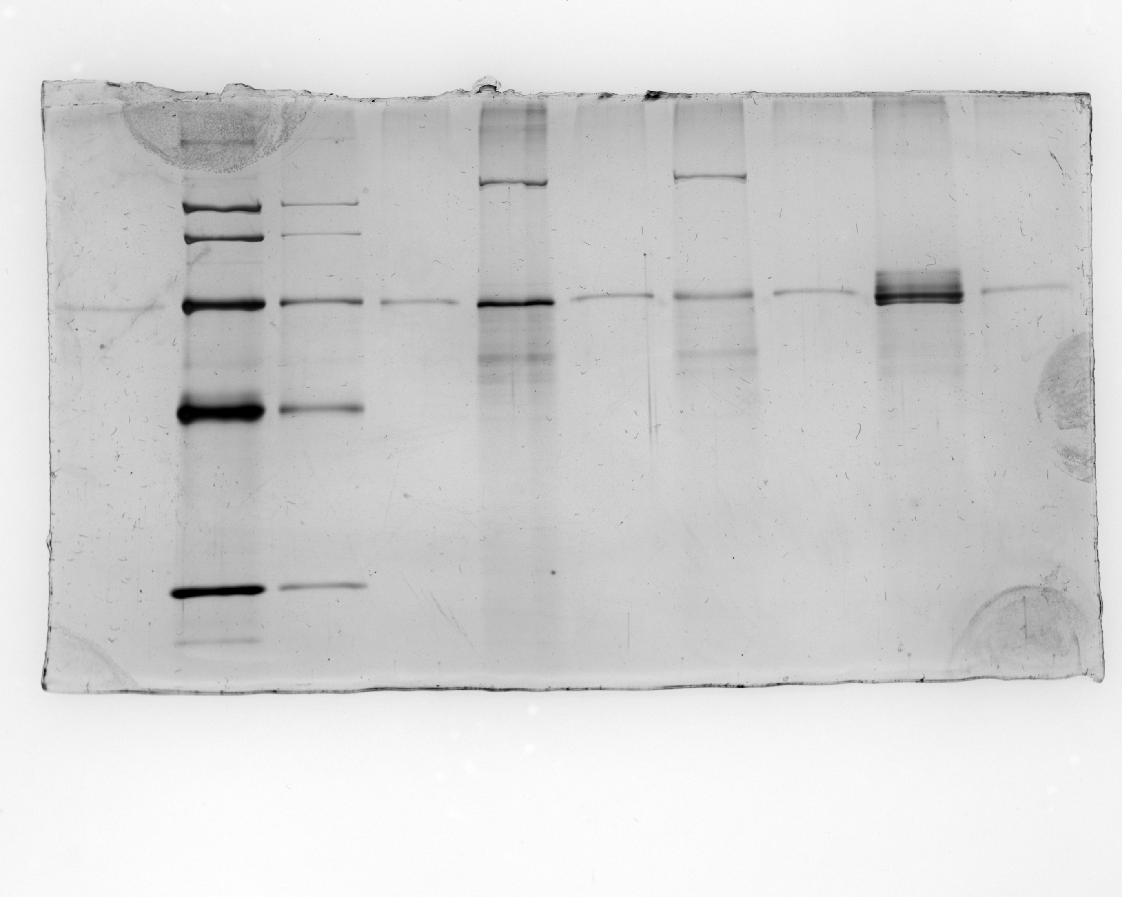

Supplement: Figure 10—source data 1. [file elife-85079-fig10-data1.zip › FIGURE 10 Source data/Fig. 10 raw data/3. Bos1-HA3 pull-down. silver stain gel (BAITS).tif]

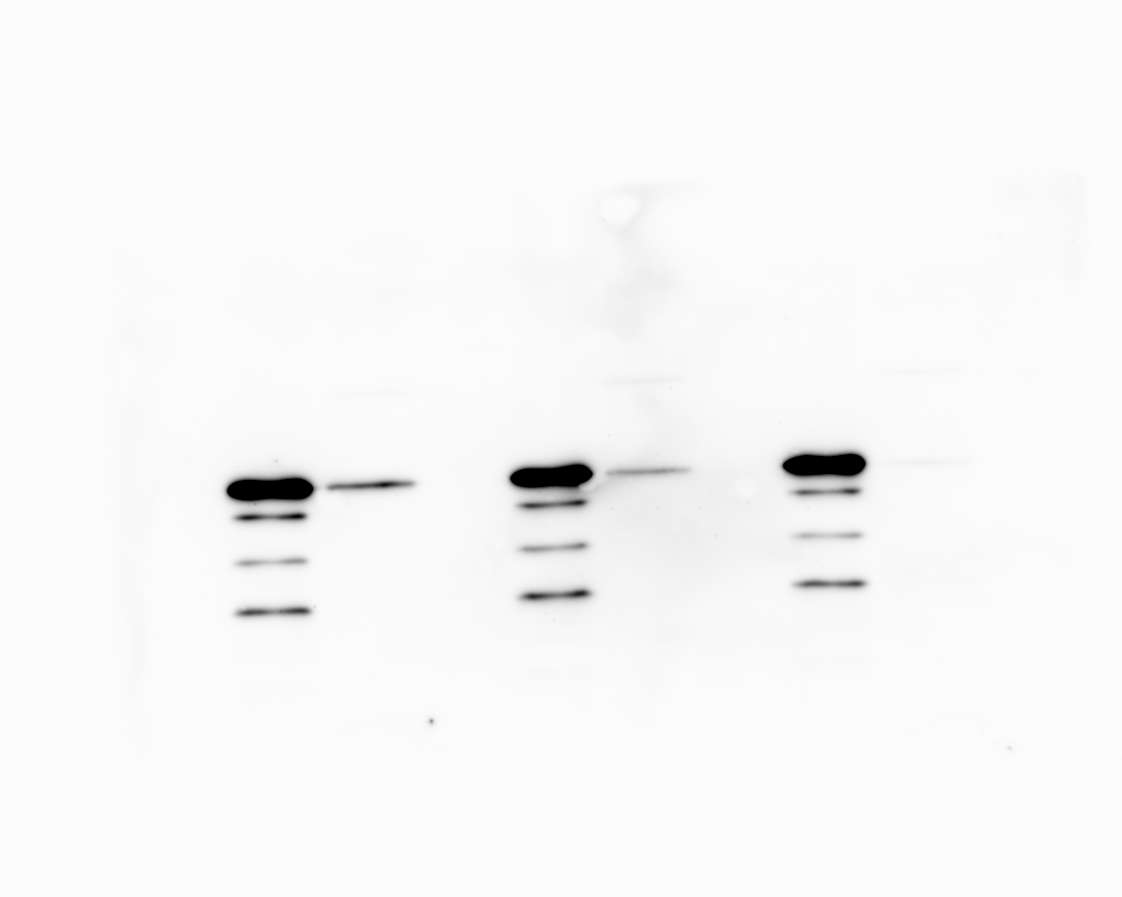

Supplement: Figure 10—source data 1. [file elife-85079-fig10-data1.zip › FIGURE 10 Source data/Fig. 10 raw data/4. Sec22-HA3 pull-down. anti-HA blot (PREY).tif]

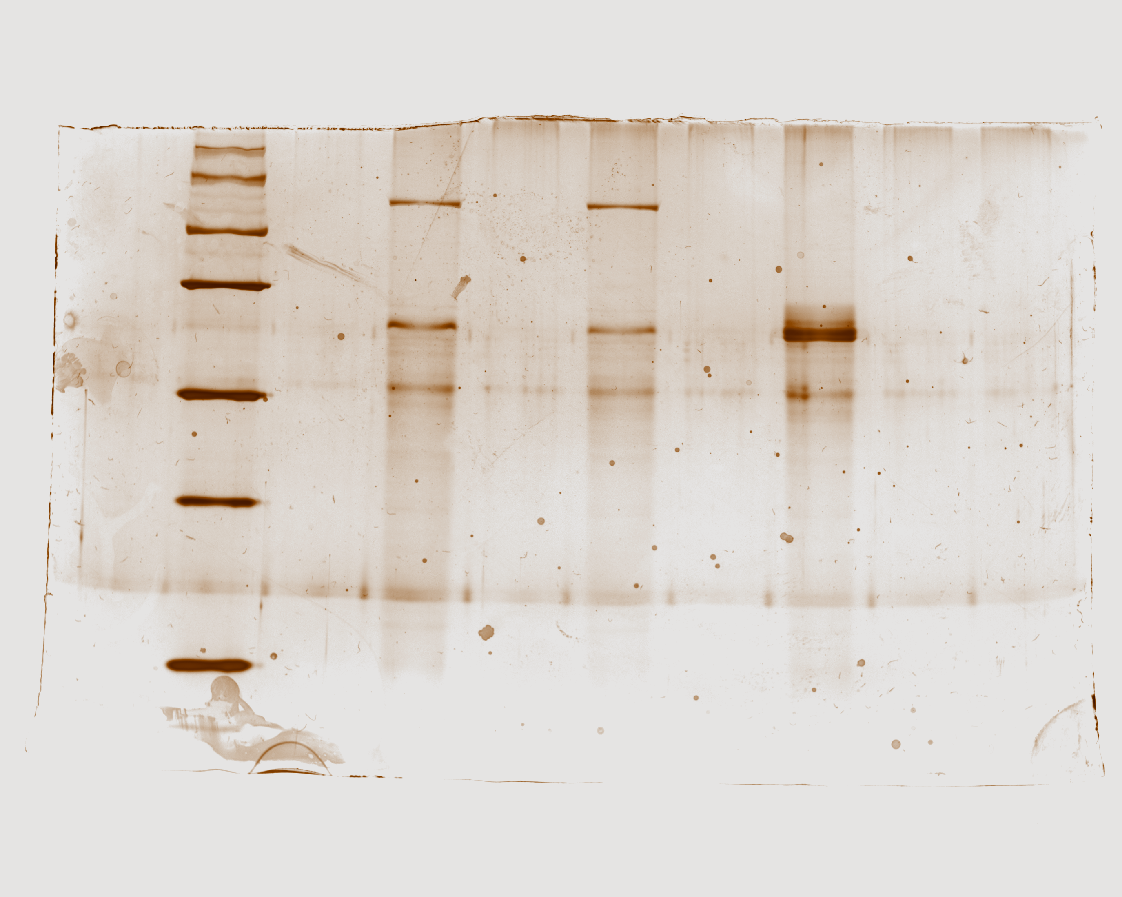

Supplement: Figure 10—source data 1. [file elife-85079-fig10-data1.zip › FIGURE 10 Source data/Fig. 10 raw data/4. Sec22-HA3 pull-down. silver stain gel (BAITS).tif]

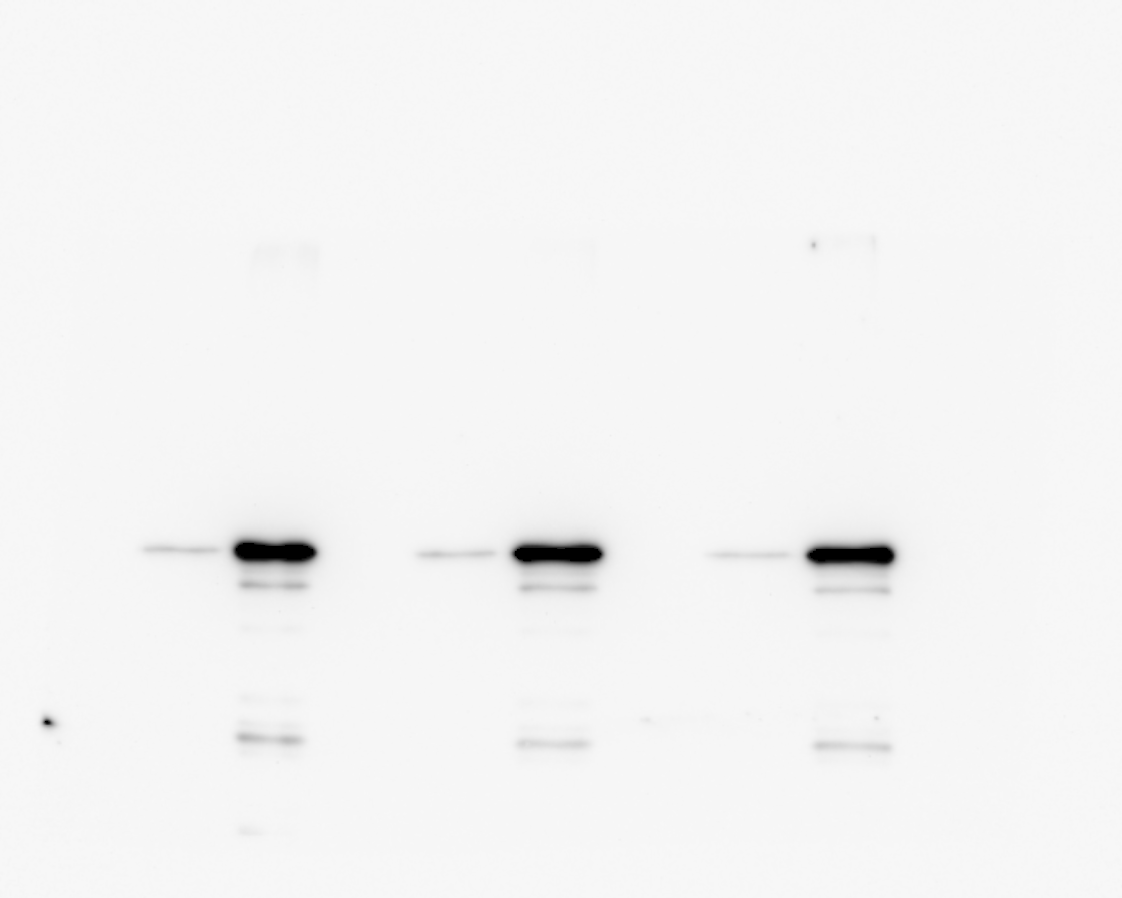

Supplement: Figure 10—source data 1. [file elife-85079-fig10-data1.zip › FIGURE 10 Source data/Fig. 10 raw data/5. Sso1-HA3 pull-down. anti-HA blot (PREY).tif]

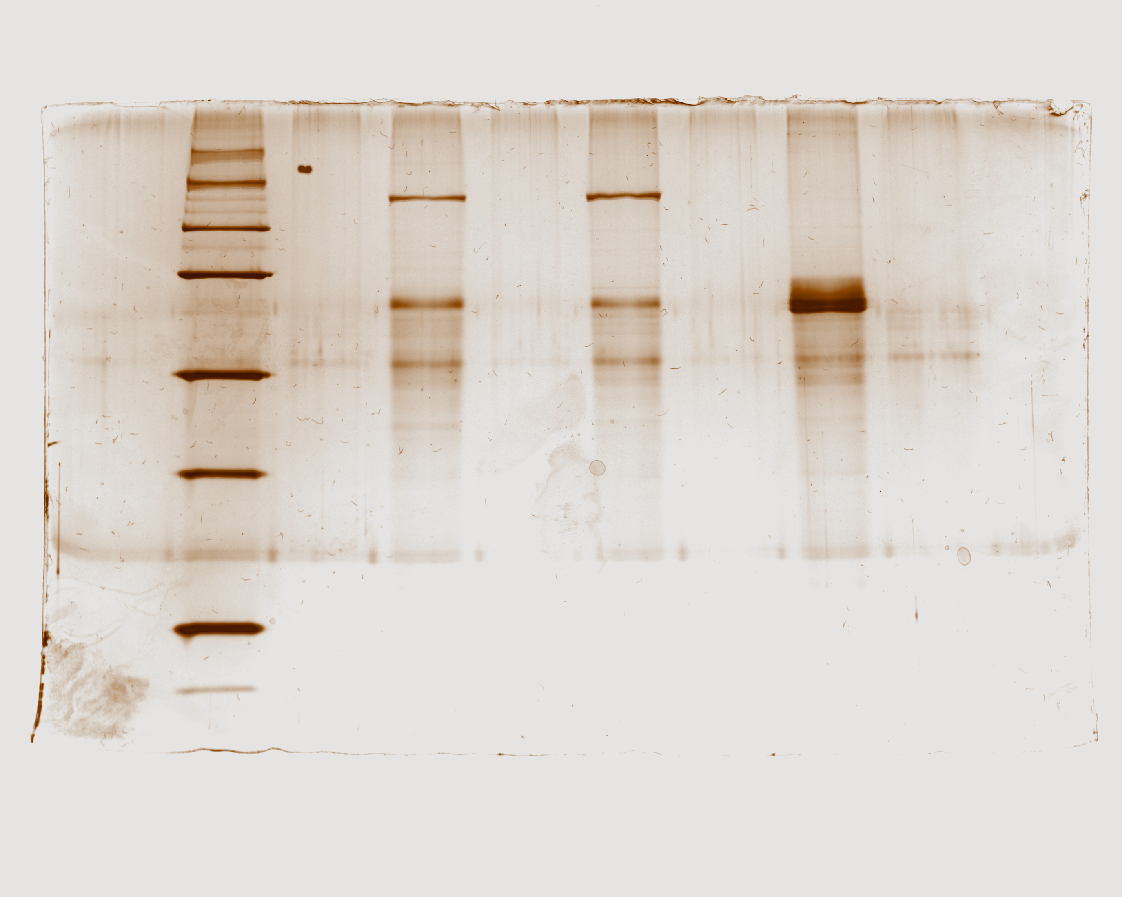

Supplement: Figure 10—source data 1. [file elife-85079-fig10-data1.zip › FIGURE 10 Source data/Fig. 10 raw data/5. Sso1-HA3 pull-down. silver stain gel (BAITS).tif]

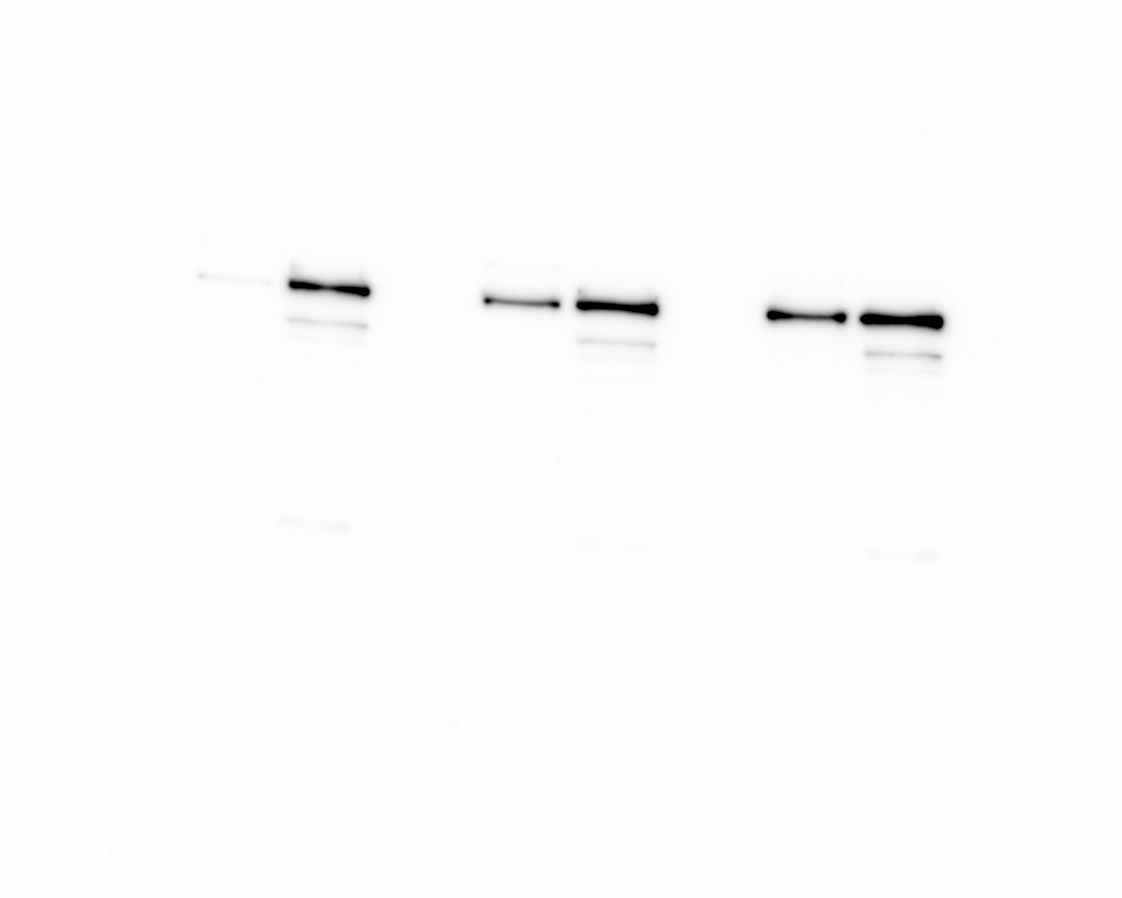

Supplement: Figure 10—source data 1. [file elife-85079-fig10-data1.zip › FIGURE 10 Source data/Fig. 10 raw data/6. Sec18-HA3 pull-down. anti-HA blot (PREY).tif]

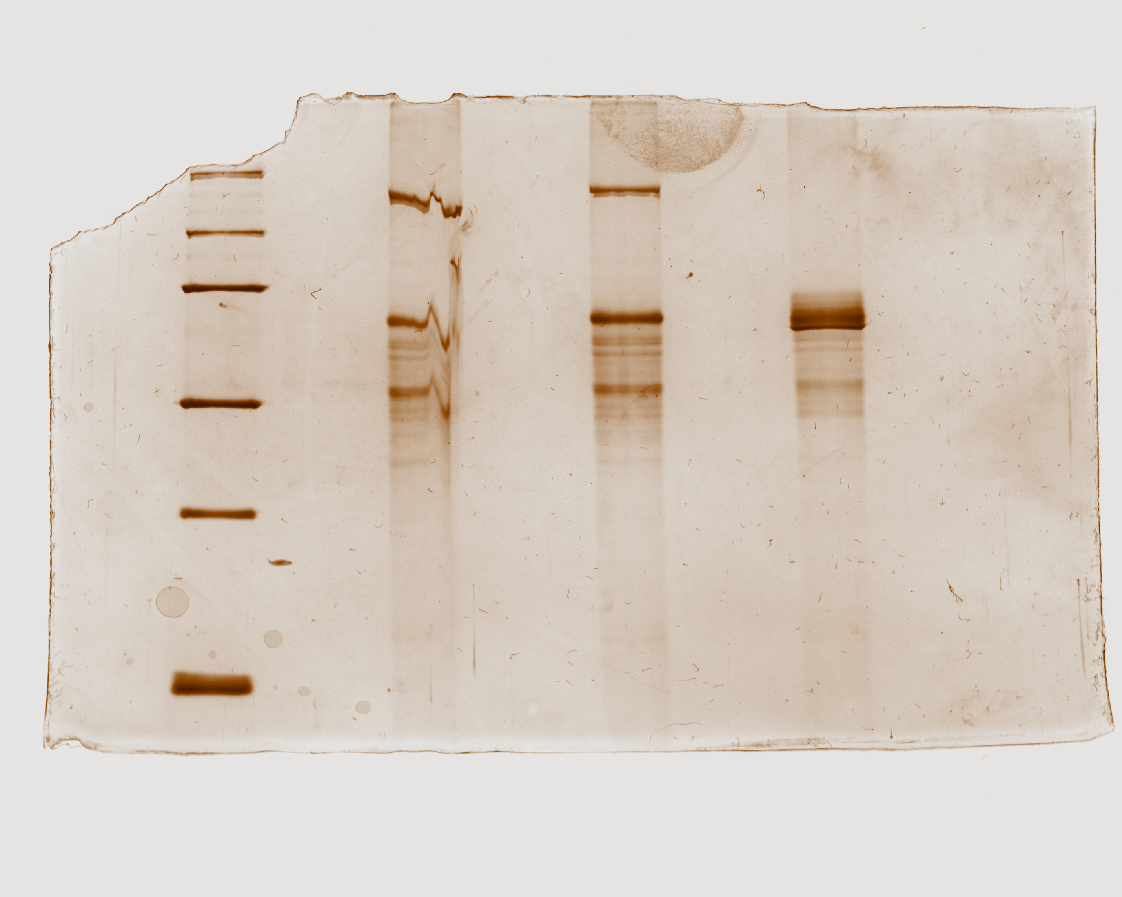

Supplement: Figure 10—source data 1. [file elife-85079-fig10-data1.zip › FIGURE 10 Source data/Fig. 10 raw data/6. Sec18-HA3 pull-down. silver stain gel (BAITS).tif]

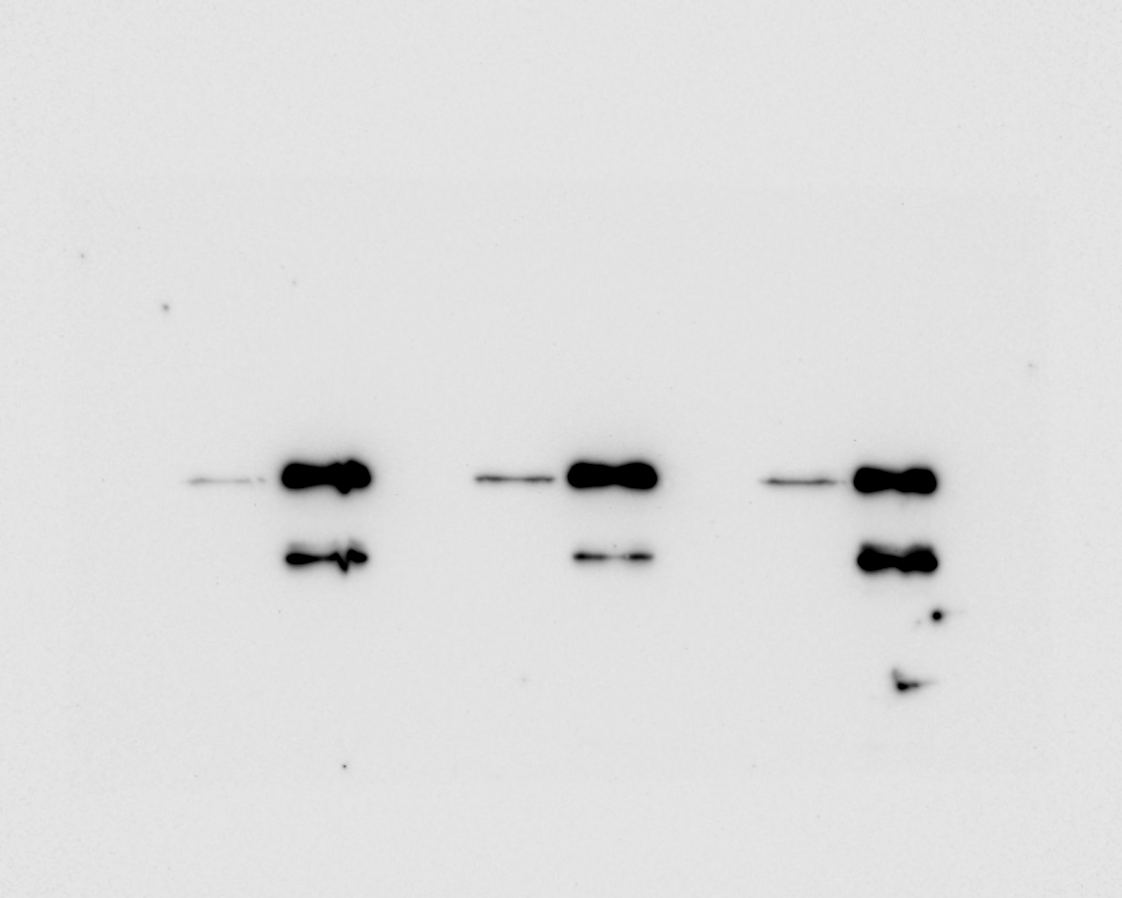

Supplement: Figure 10—source data 1. [file elife-85079-fig10-data1.zip › FIGURE 10 Source data/Fig. 10 raw data/7. Grh1-HA3 pull-down. anti-HA blot (PREY).tif]

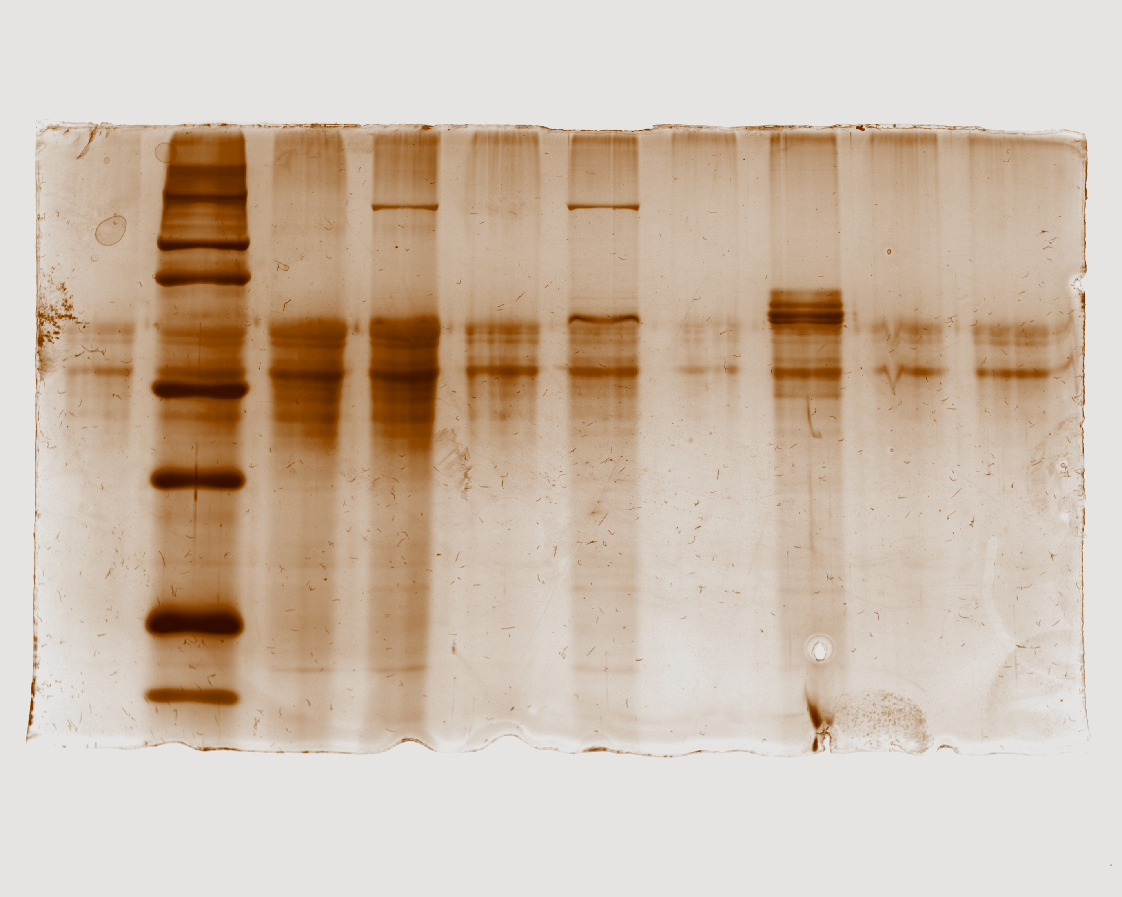

Supplement: Figure 10—source data 1. [file elife-85079-fig10-data1.zip › FIGURE 10 Source data/Fig. 10 raw data/7. Grh1-HA3 pull-down. silver stain gel (BAITS).tif]

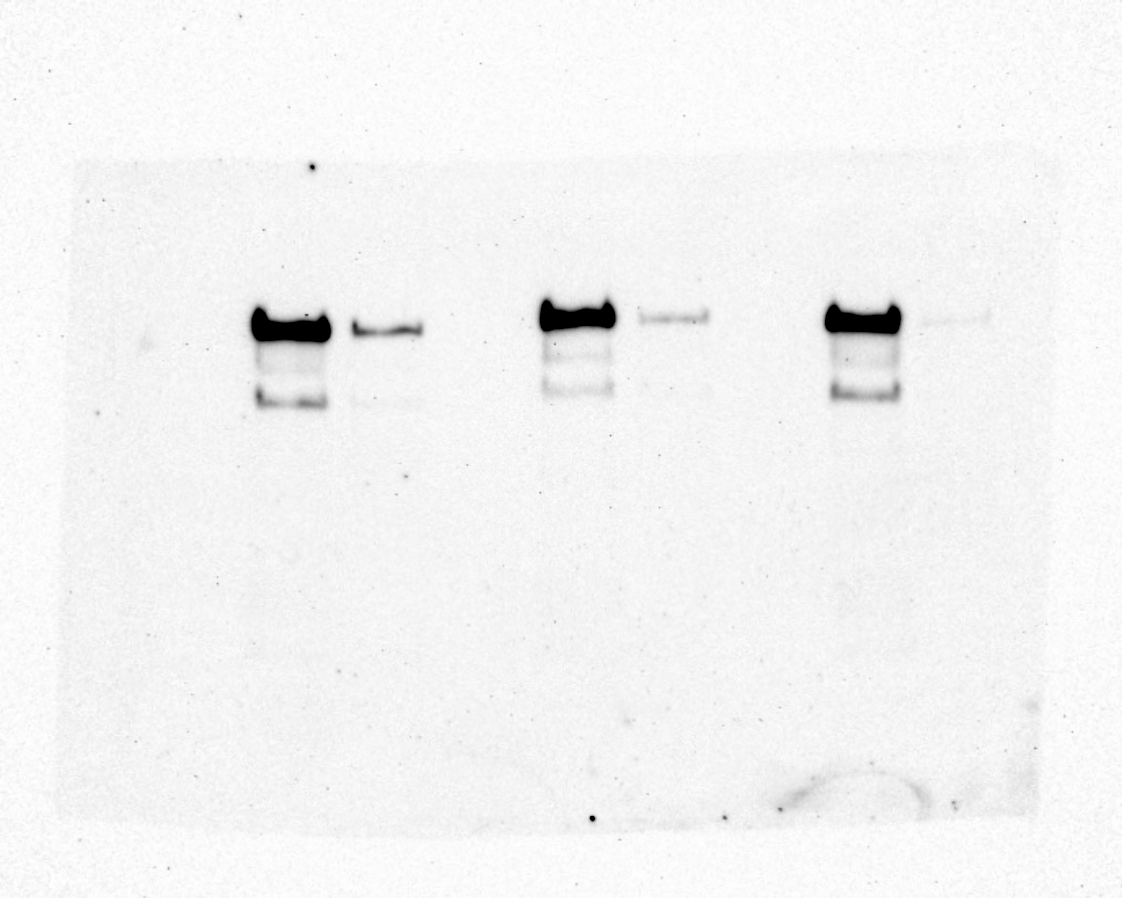

Supplement: Figure 10—source data 1. [file elife-85079-fig10-data1.zip › FIGURE 10 Source data/Fig. 10 raw data/8. Bug1-HA3 pull-down. anti-HA blot (PREY).tif]

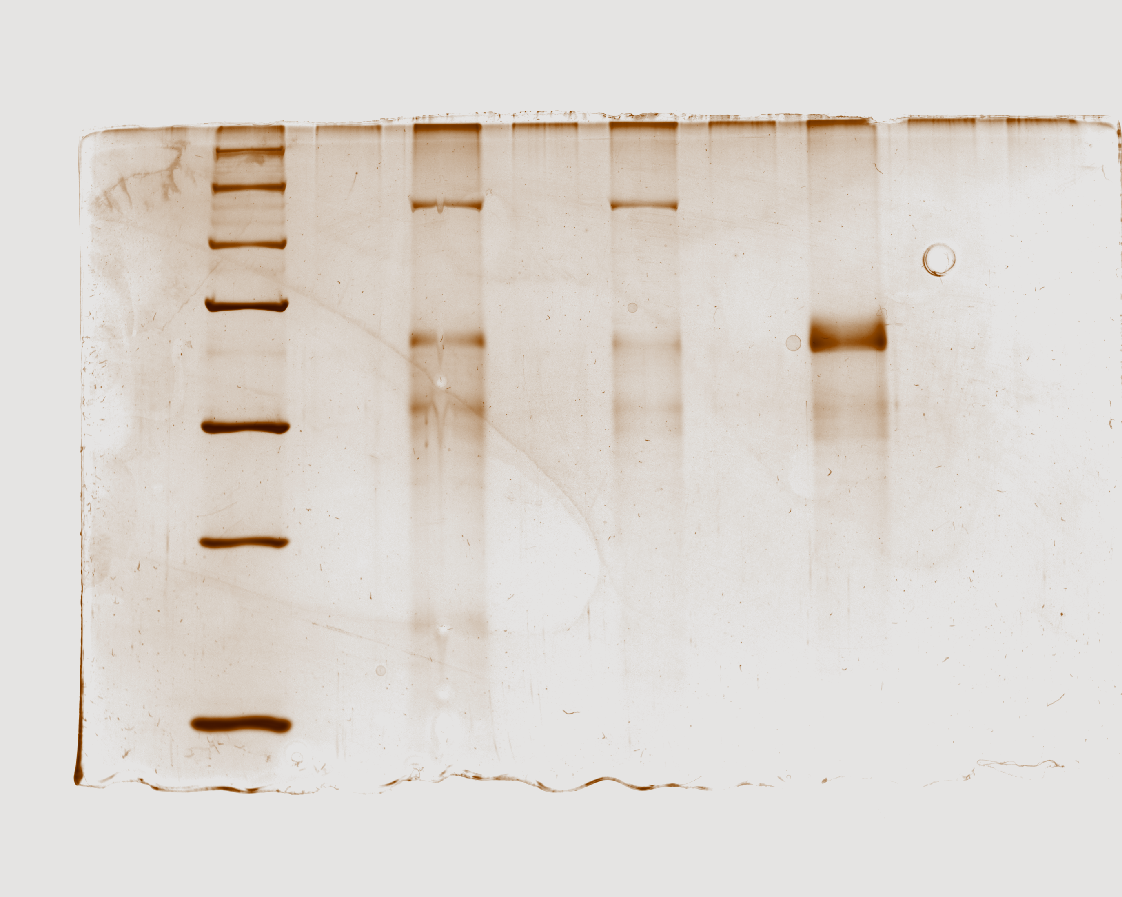

Supplement: Figure 10—source data 1. [file elife-85079-fig10-data1.zip › FIGURE 10 Source data/Fig. 10 raw data/8. Bug1-HA3 pull-down. silver stain gel (BAITS).tif]

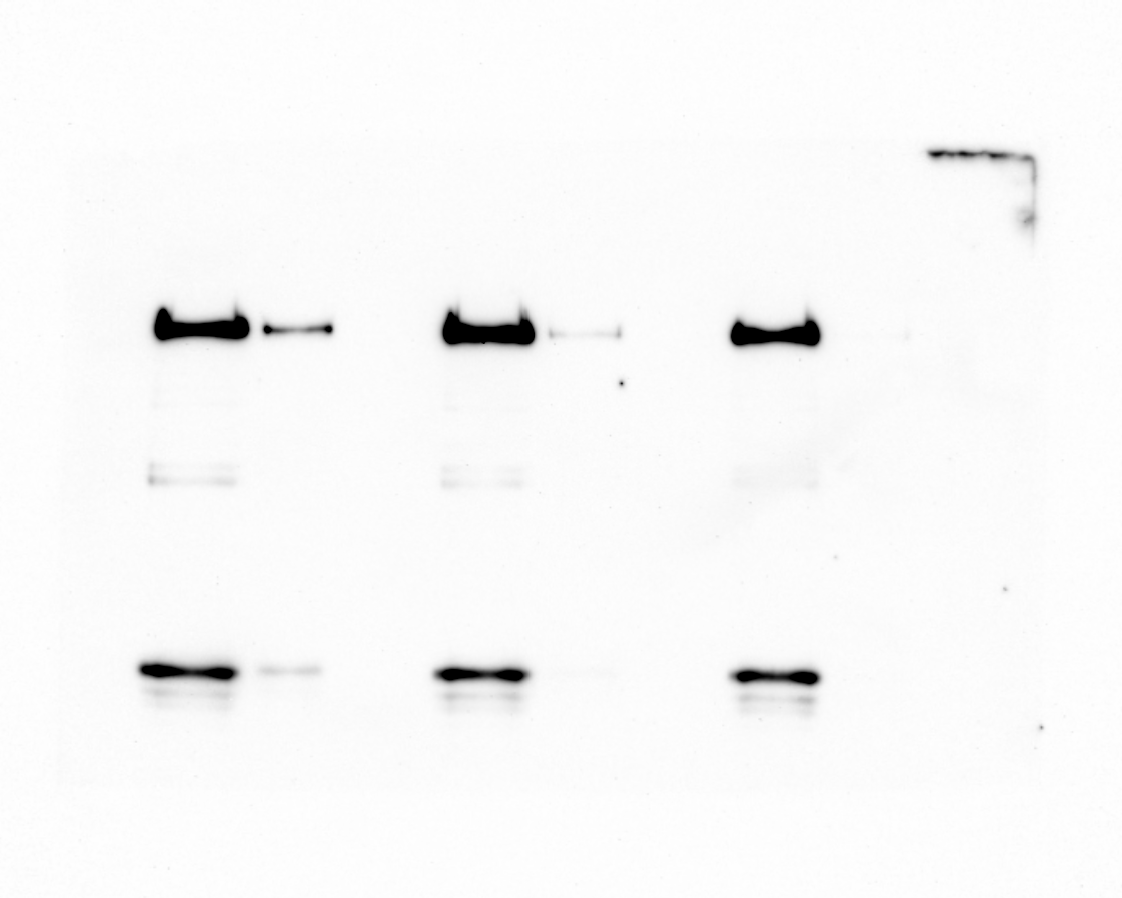

Supplement: Figure 10—source data 1. [file elife-85079-fig10-data1.zip › FIGURE 10 Source data/Fig. 10 raw data/9. Coy1-HA3 pull-down. anti-HA blot (PREY).tif]

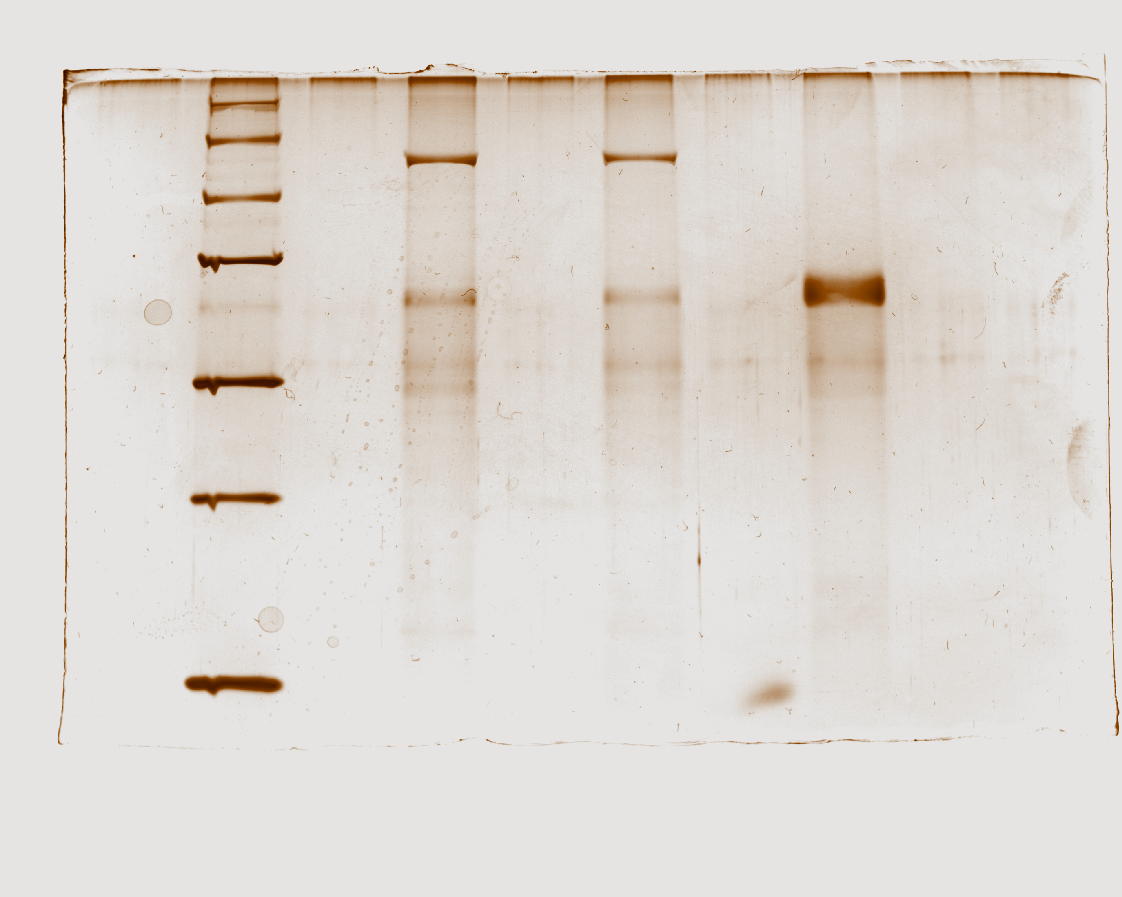

Supplement: Figure 10—source data 1. [file elife-85079-fig10-data1.zip › FIGURE 10 Source data/Fig. 10 raw data/9. Coy1-HA3 pull-down. silver stain gel (BAITS).tif]

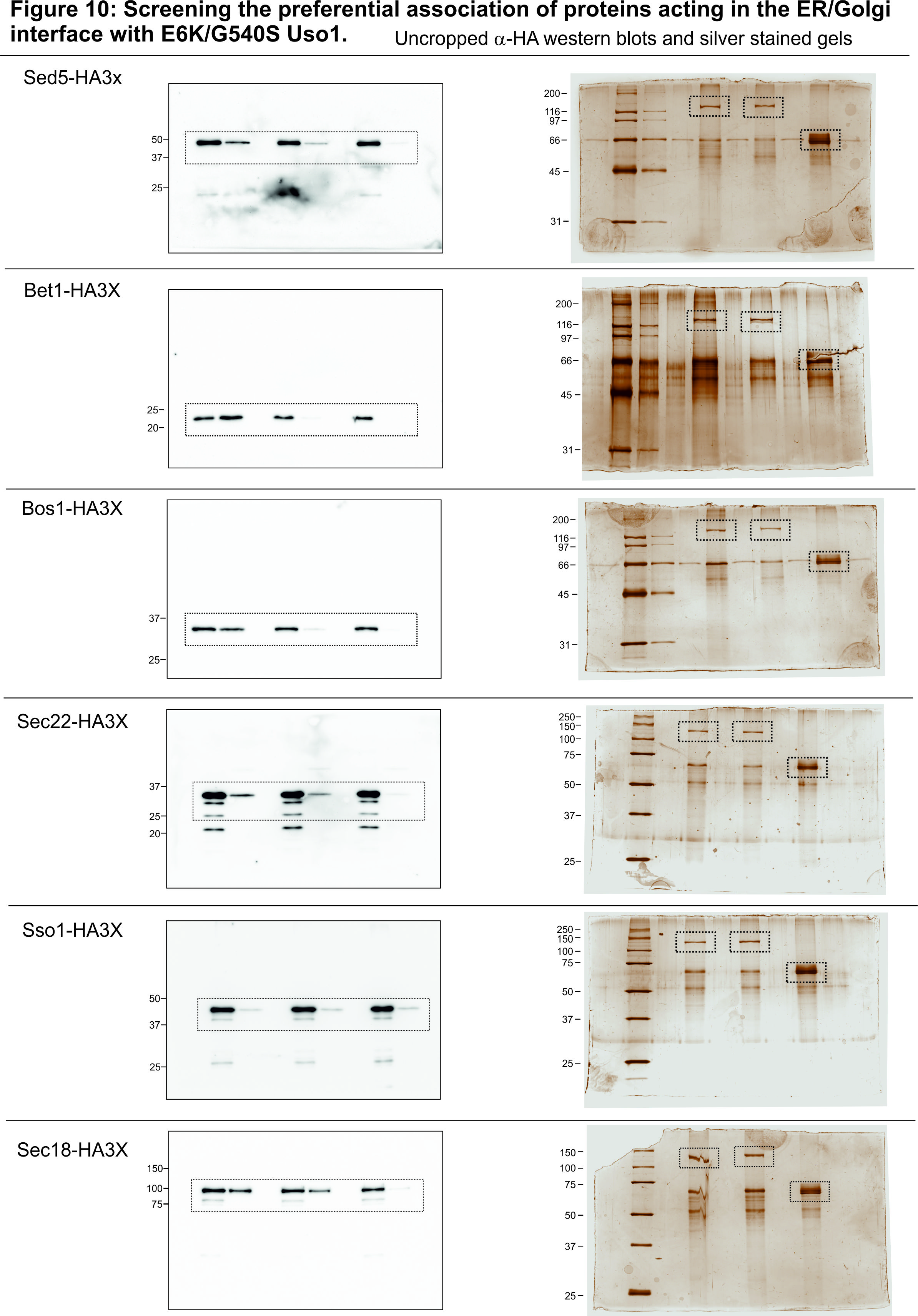

Supplement: Figure 10—source data 1. [file elife-85079-fig10-data1.zip › FIGURE 10 Source data/uncropped images Figure 10 (I).jpg]

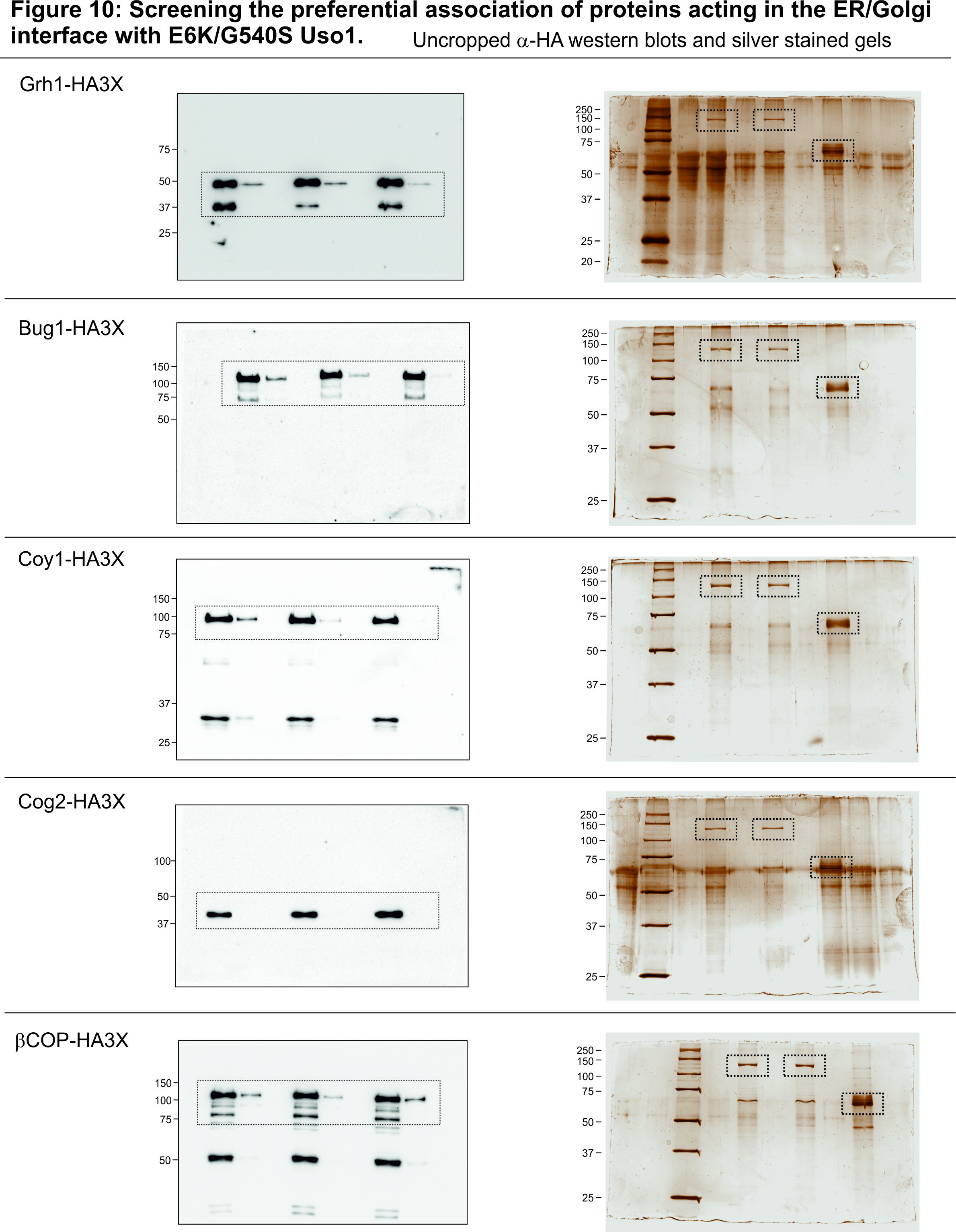

Supplement: Figure 10—source data 1. [file elife-85079-fig10-data1.zip › FIGURE 10 Source data/uncropped images Figure 10 (II).jpg]

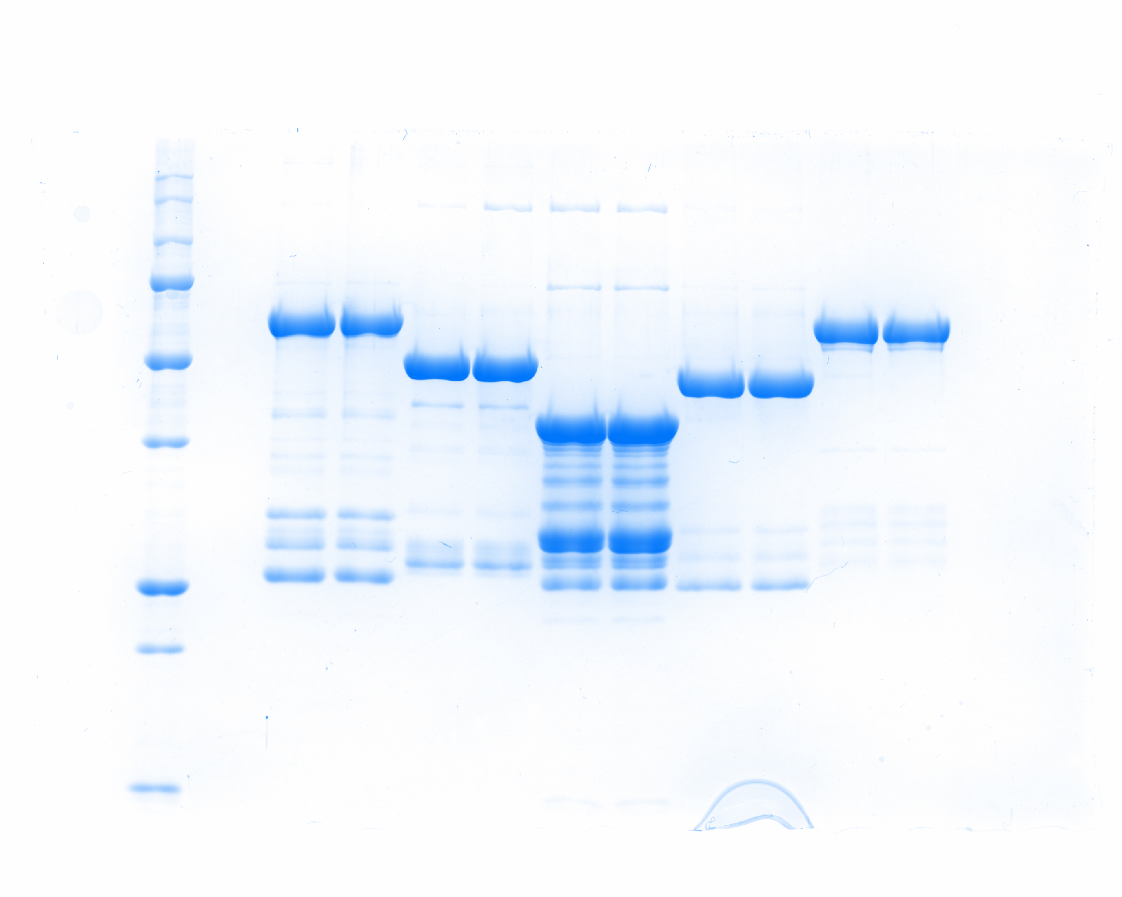

Supplement: Figure 11—source data 1. [file elife-85079-fig11-data1.zip › FIGURE 11 Source data/RAW data/Fig 11 panel A/Figure 11. panel A. Uso1-His6 pull-down. SNARE-GST coomassie.tif]

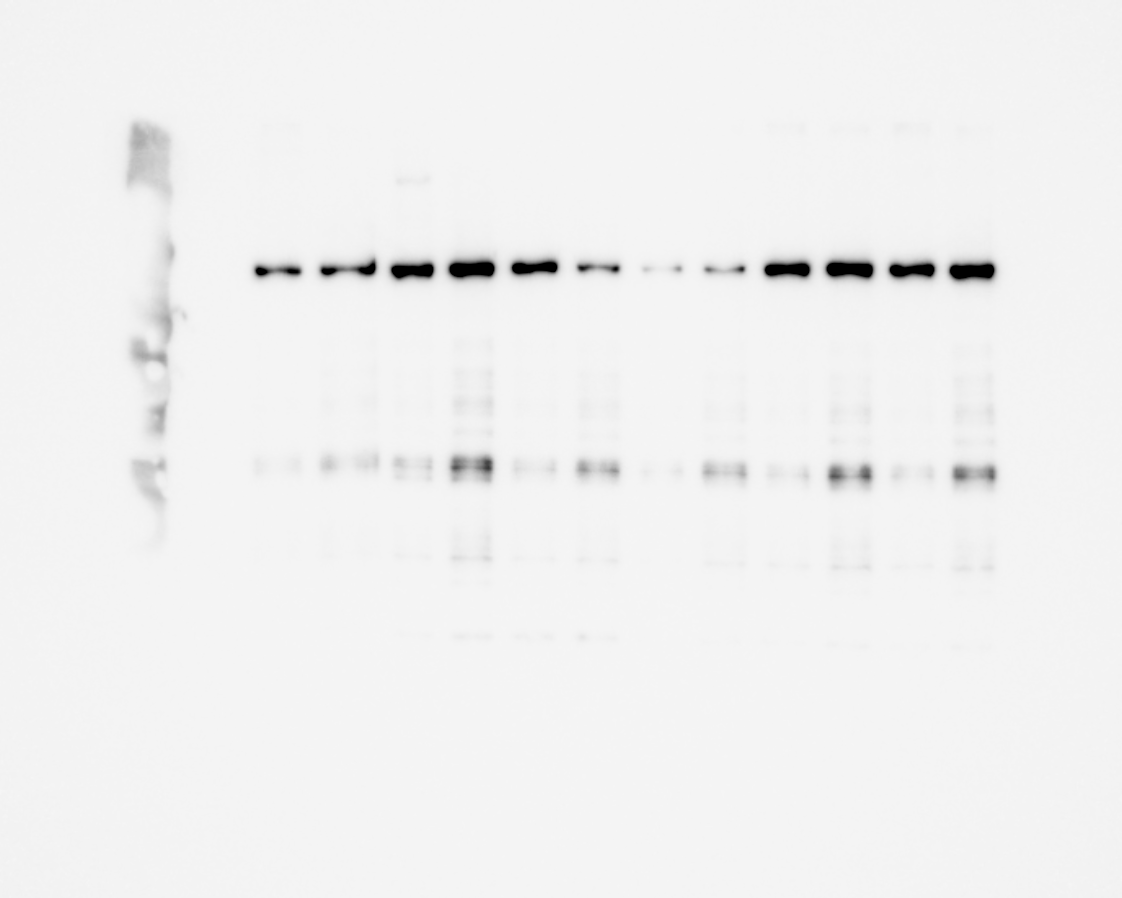

Supplement: Figure 11—source data 1. [file elife-85079-fig11-data1.zip › FIGURE 11 Source data/RAW data/Fig 11 panel A/Figure 11. panel A. Uso1-His6 pull-down. WB anti-His tag. Flow-through.tif]

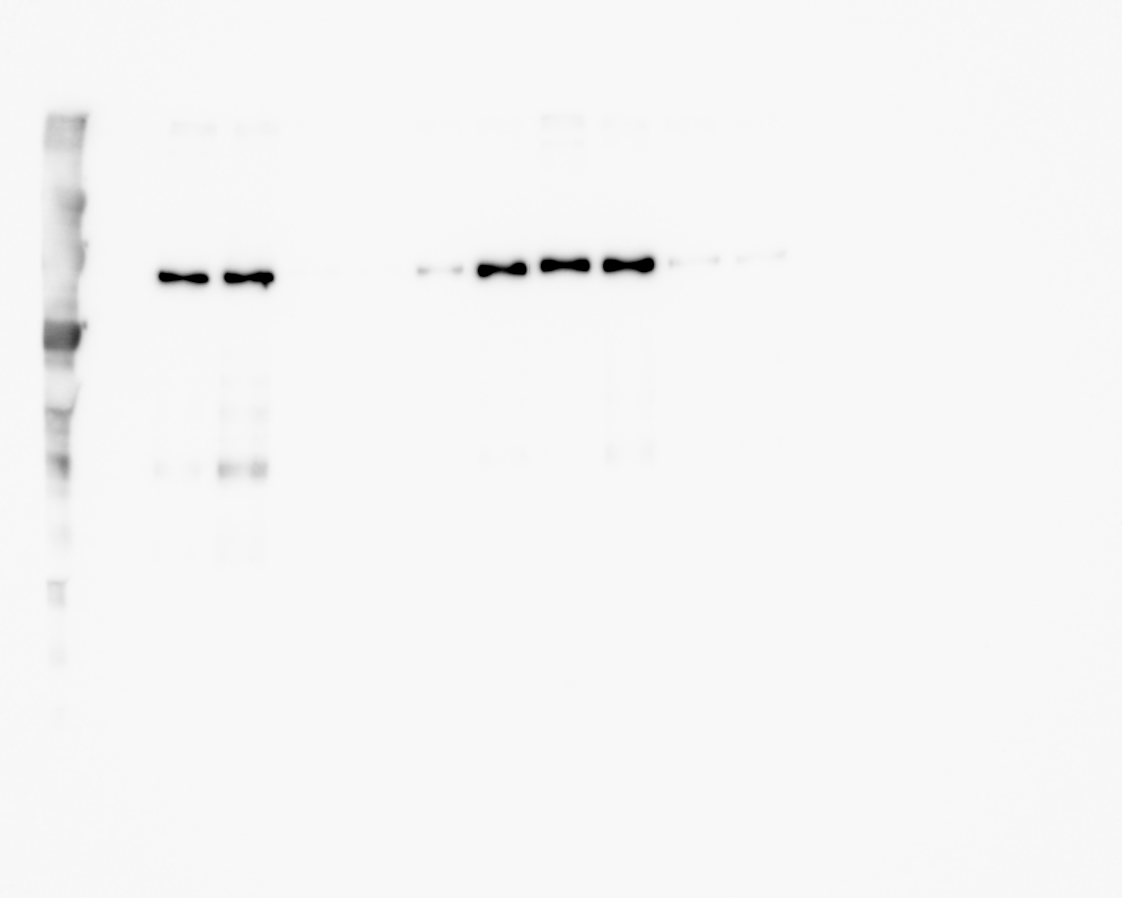

Supplement: Figure 11—source data 1. [file elife-85079-fig11-data1.zip › FIGURE 11 Source data/RAW data/Fig 11 panel A/Figure 11. panel A. Uso1-His6 pull-down. WB bound prey (+exp).tif]

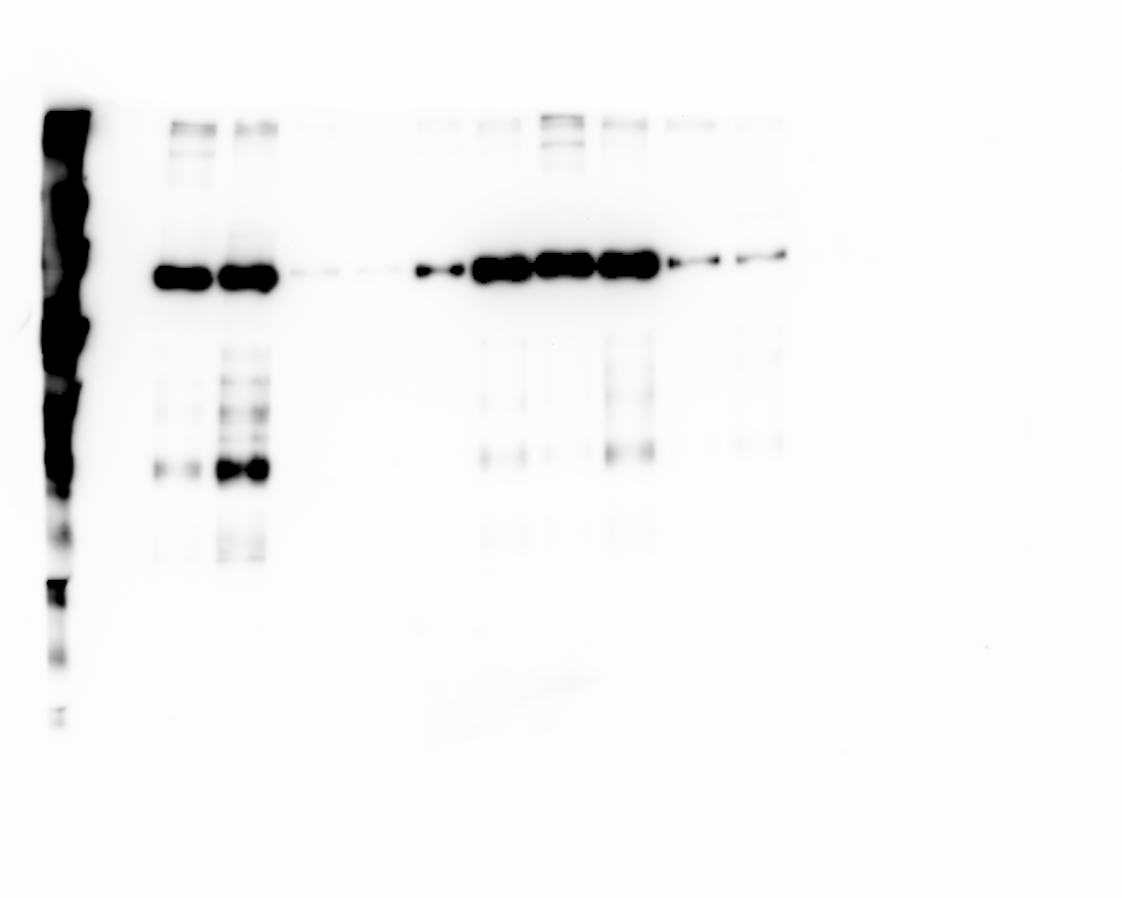

Supplement: Figure 11—source data 1. [file elife-85079-fig11-data1.zip › FIGURE 11 Source data/RAW data/Fig 11 panel A/Figure 11. panel A. Uso1-His6 pull-down. WB bound prey.tif]

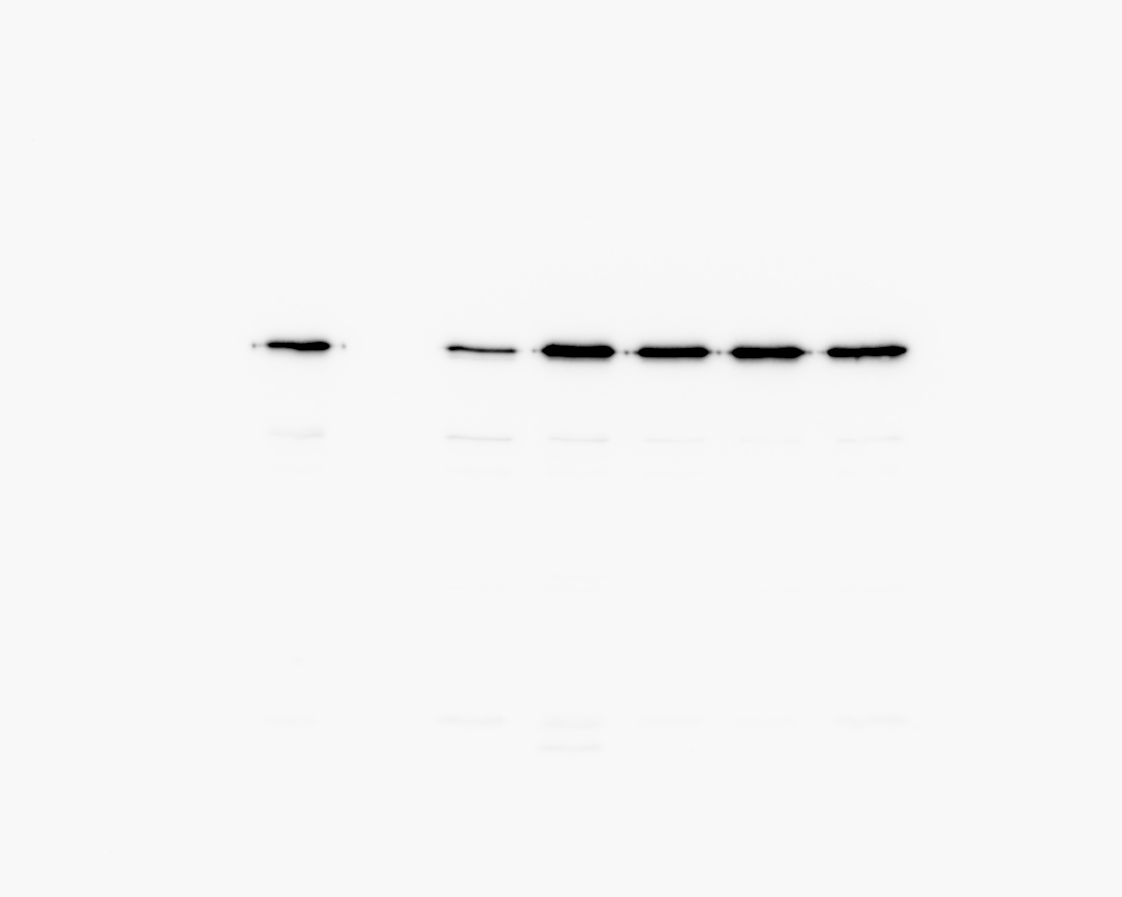

Supplement: Figure 11—source data 1. [file elife-85079-fig11-data1.zip › FIGURE 11 Source data/RAW data/Fig 11 panel C/Figure 11. panel C. Sly1-HA pull-down. Flow-through.tif]

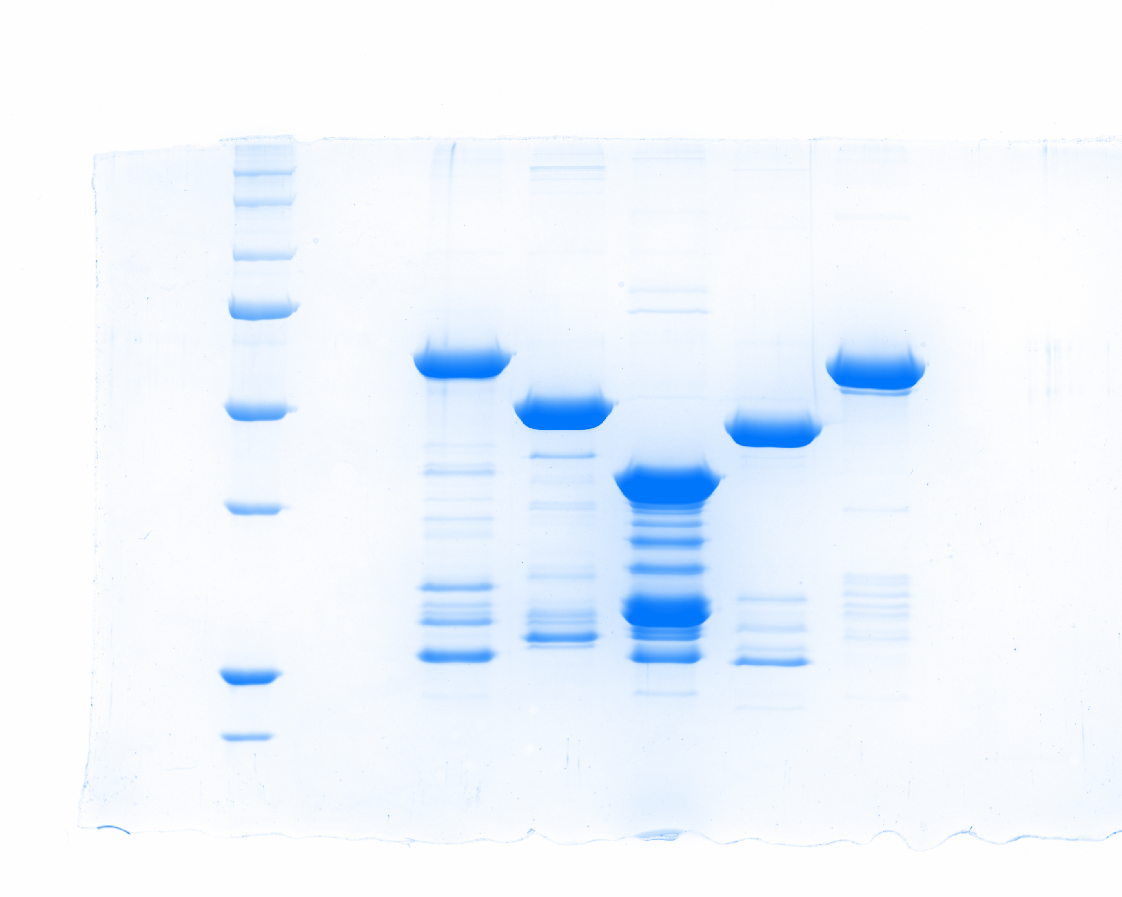

Supplement: Figure 11—source data 1. [file elife-85079-fig11-data1.zip › FIGURE 11 Source data/RAW data/Fig 11 panel C/Figure 11. panel C. Sly1-HA pull-down. SNARE-GST coomassie.tif]

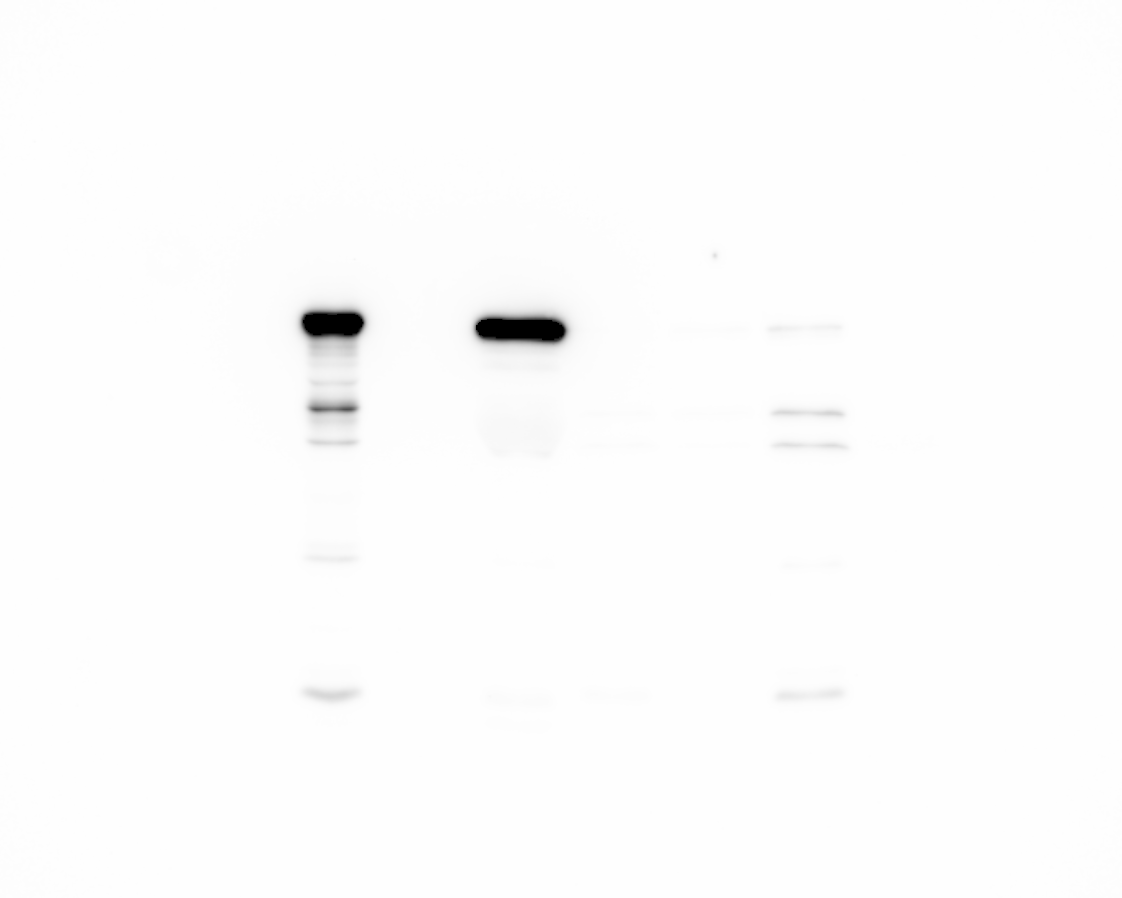

Supplement: Figure 11—source data 1. [file elife-85079-fig11-data1.zip › FIGURE 11 Source data/RAW data/Fig 11 panel C/Figure 11. panel C. Sly1-HA pull-down. WB bound prey (+exp).tif]

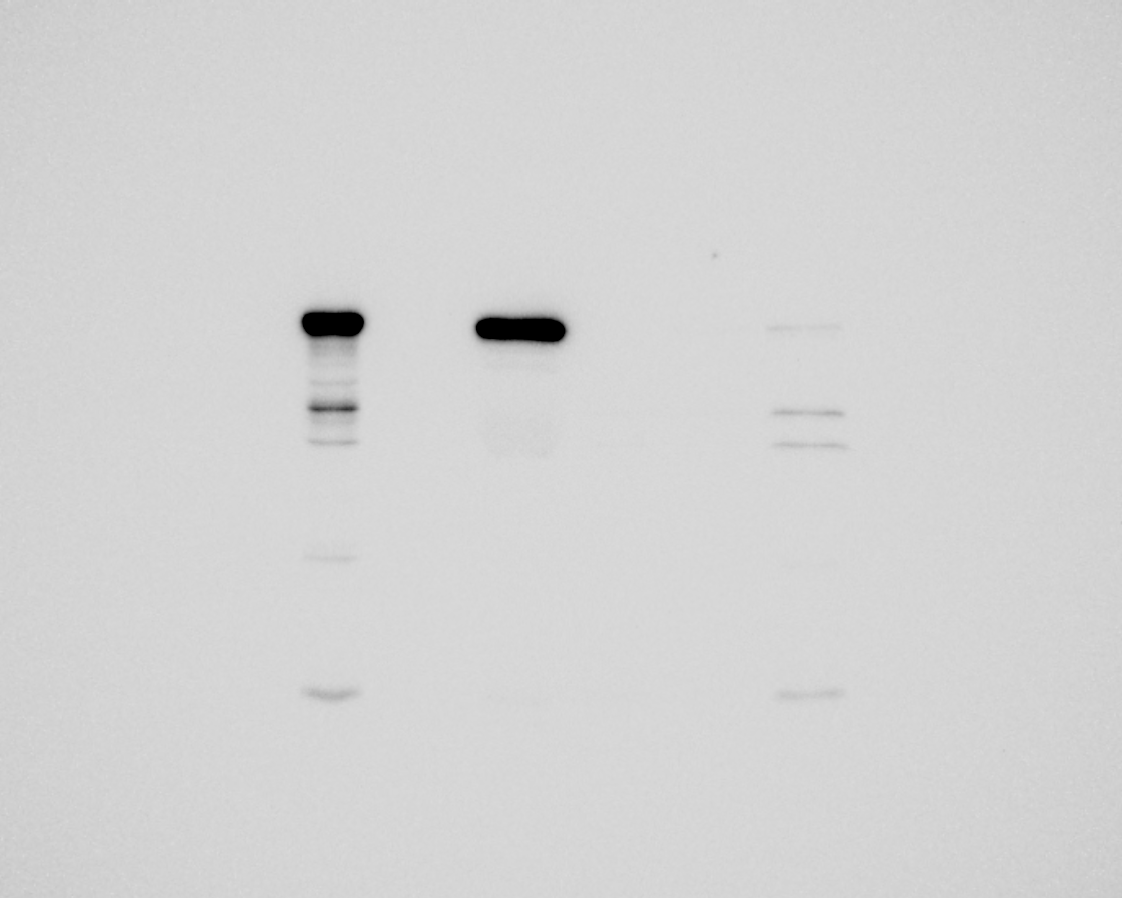

Supplement: Figure 11—source data 1. [file elife-85079-fig11-data1.zip › FIGURE 11 Source data/RAW data/Fig 11 panel C/Figure 11. panel C. Sly1-HA pull-down. WB bound prey.tif]

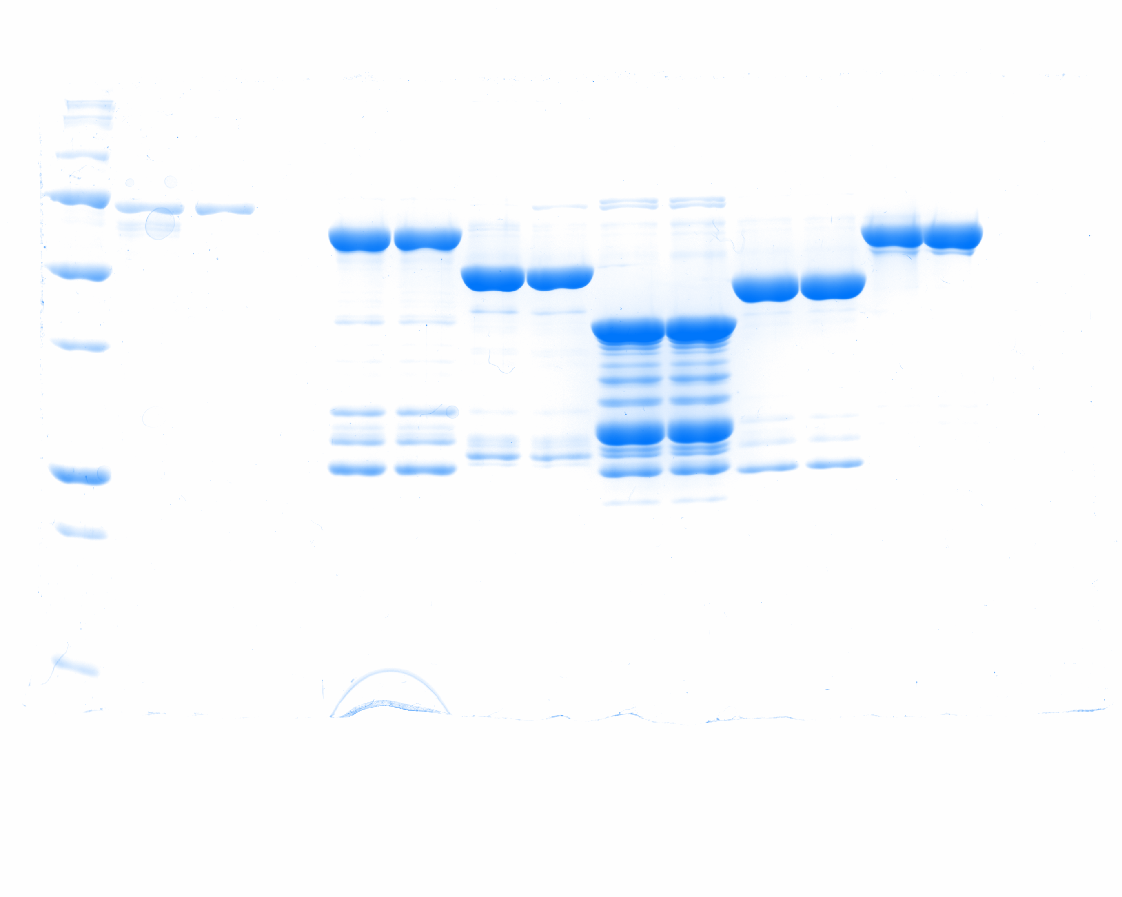

Supplement: Figure 11—source data 1. [file elife-85079-fig11-data1.zip › FIGURE 11 Source data/RAW data/Fig 11 panel D/Figure 11. panel D. Uso1 GHD-His6 pull-down. SNARE-GST coomassie.tif]

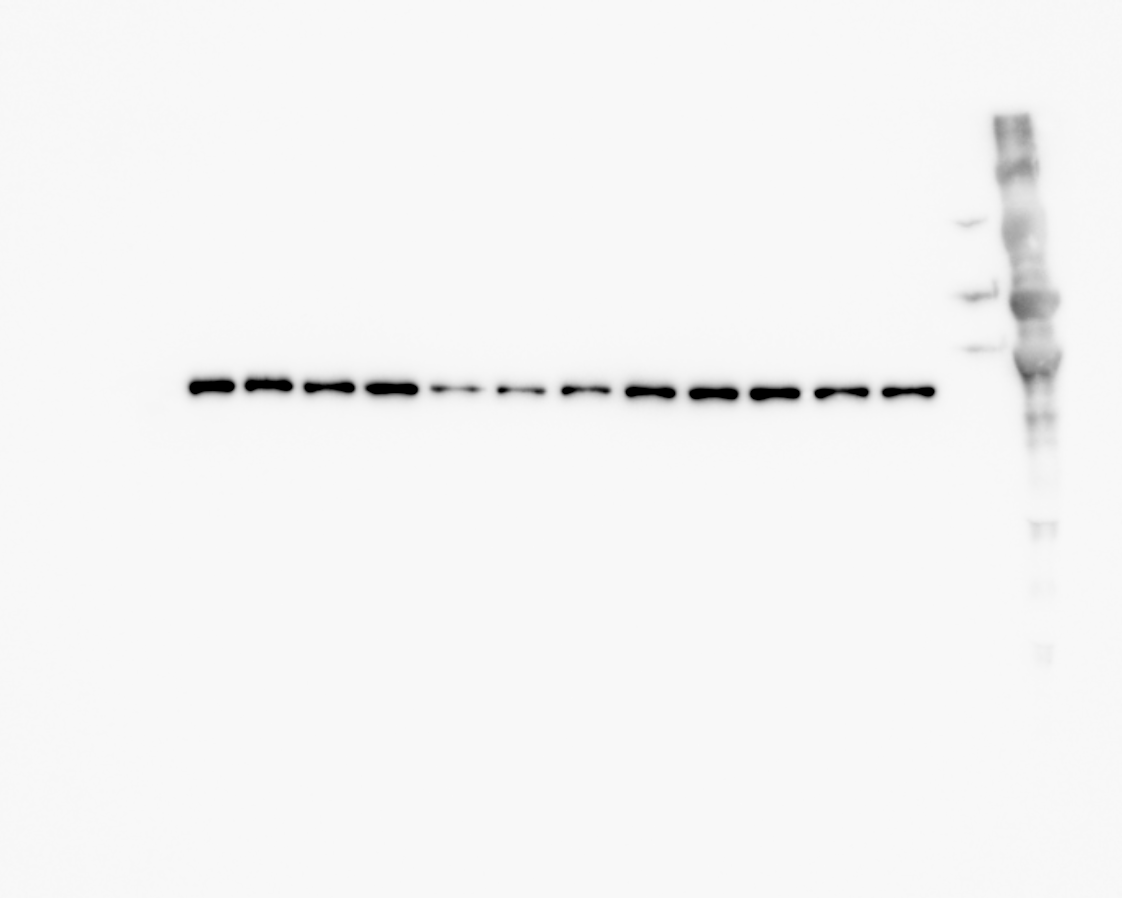

Supplement: Figure 11—source data 1. [file elife-85079-fig11-data1.zip › FIGURE 11 Source data/RAW data/Fig 11 panel D/Figure 11. panel D. Uso1 GHD-His6 pull-down. WB anti-His tag. Flow-through.tif]

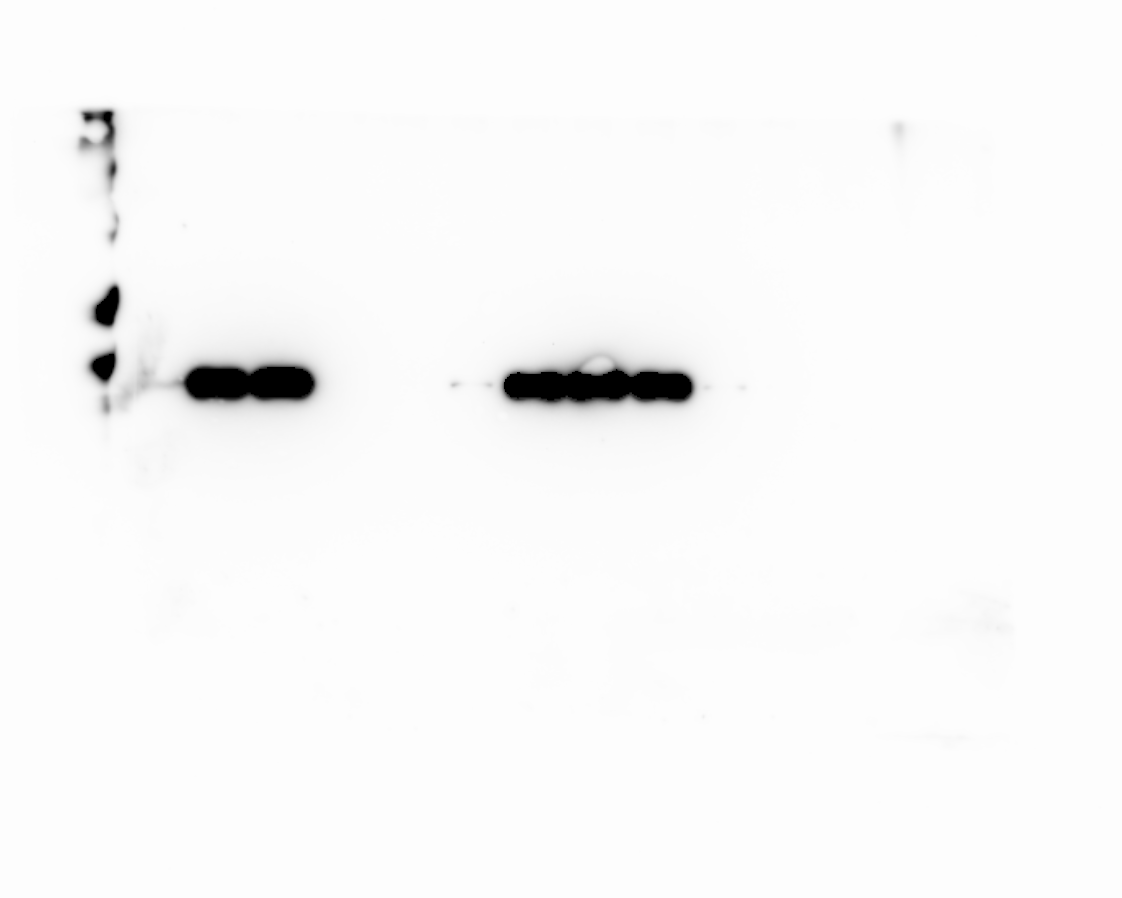

Supplement: Figure 11—source data 1. [file elife-85079-fig11-data1.zip › FIGURE 11 Source data/RAW data/Fig 11 panel D/Figure 11. panel D. Uso1 GHD-His6 pull-down. WB bound prey (+exp).tif]

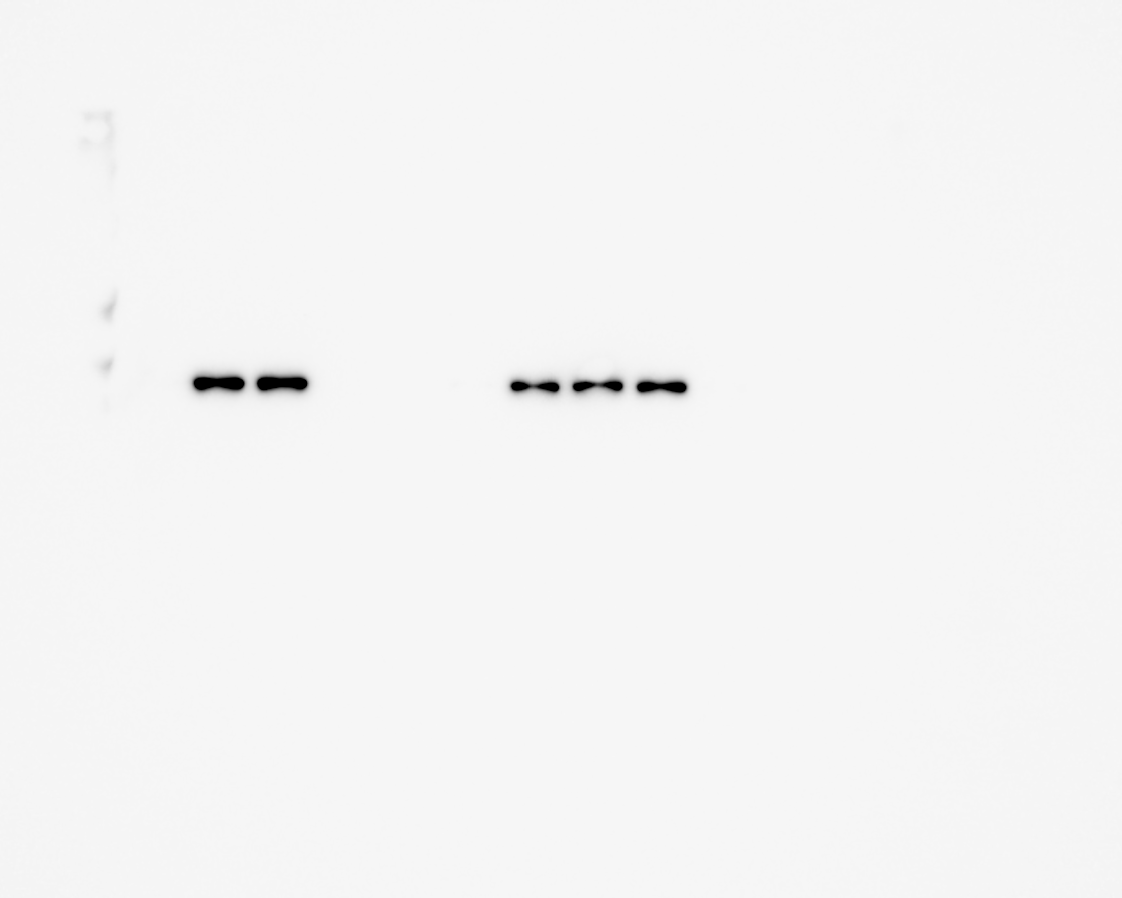

Supplement: Figure 11—source data 1. [file elife-85079-fig11-data1.zip › FIGURE 11 Source data/RAW data/Fig 11 panel D/Figure 11. panel D. Uso1 GHD-His6 pull-down. WB bound prey.tif]

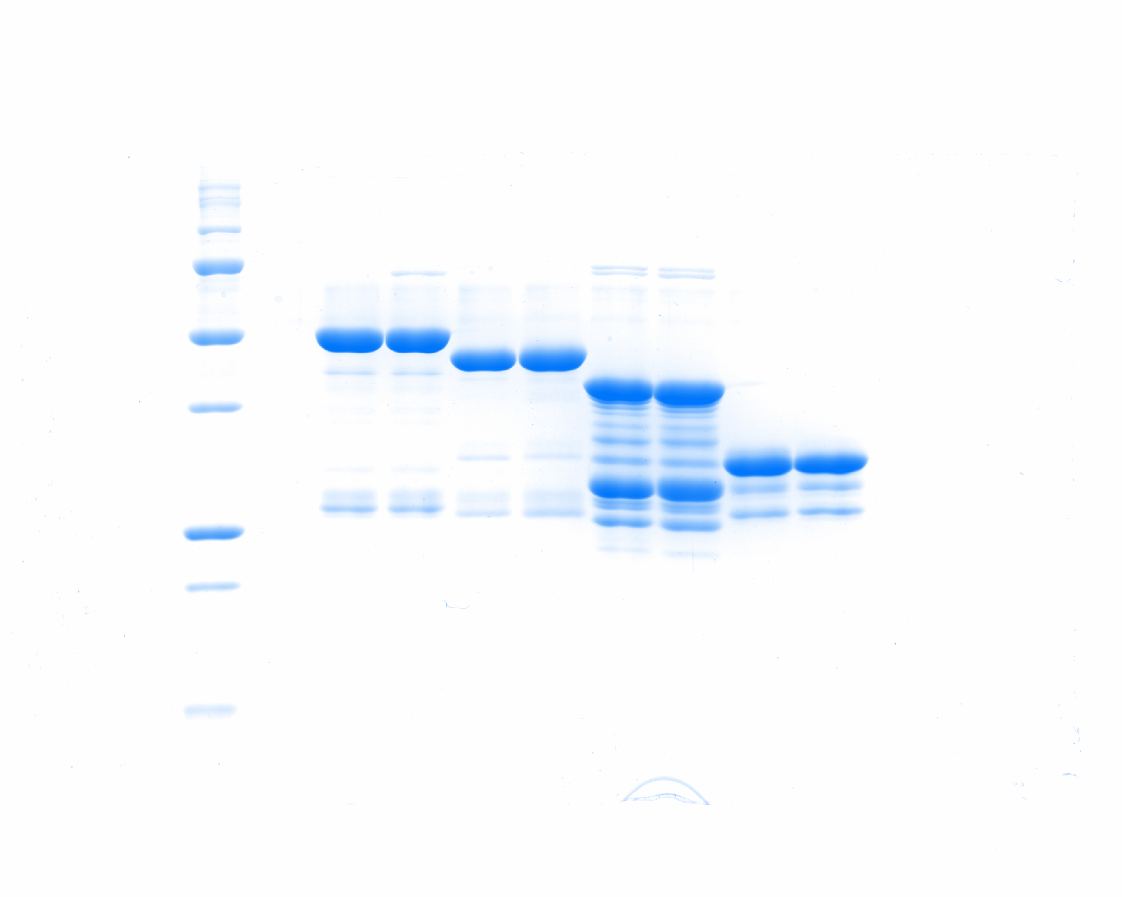

Supplement: Figure 11—source data 1. [file elife-85079-fig11-data1.zip › FIGURE 11 Source data/RAW data/Fig 11 panel F/Figure 11. panel F. Uso1 GHD-His6 pull-down. SNARE-GST coomassie.tif]

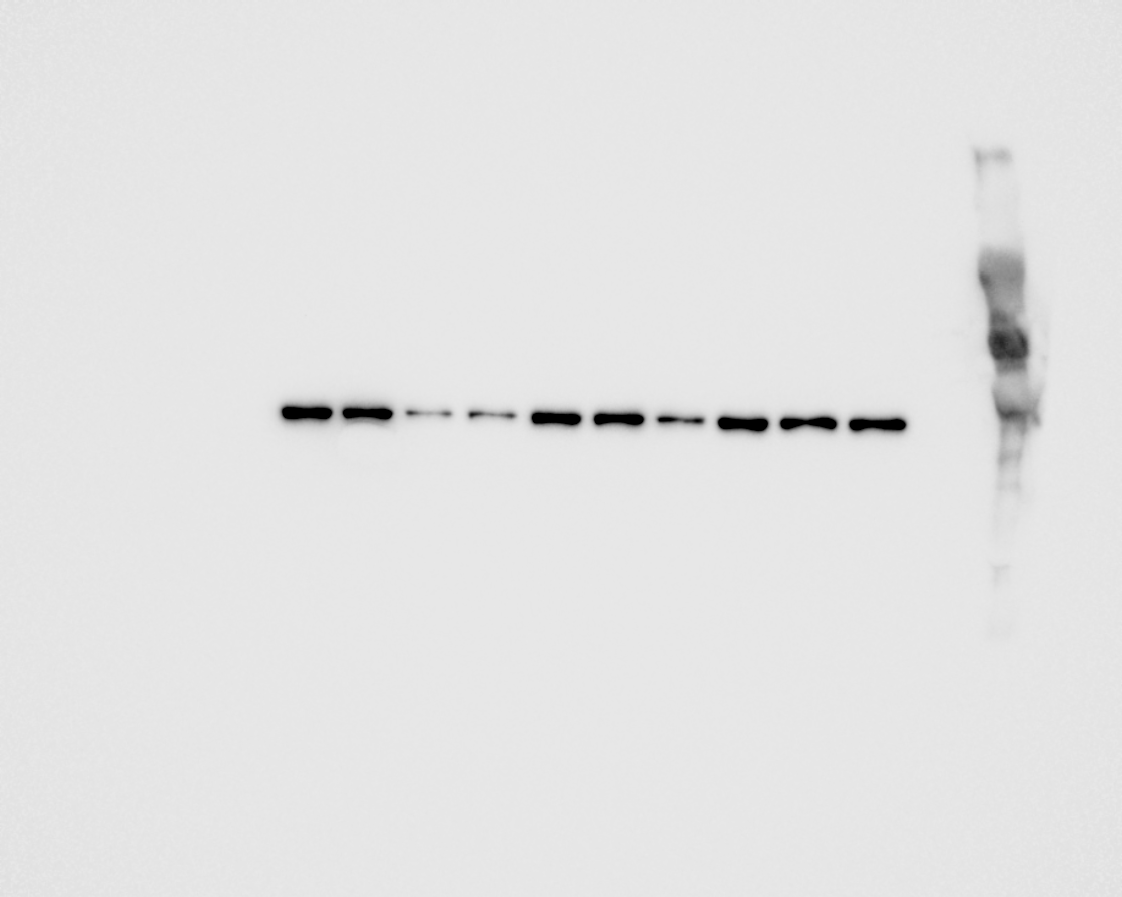

Supplement: Figure 11—source data 1. [file elife-85079-fig11-data1.zip › FIGURE 11 Source data/RAW data/Fig 11 panel F/Figure 11. panel F. Uso1 GHD-His6 pull-down. WB anti-His tag . Flow-through.tif]

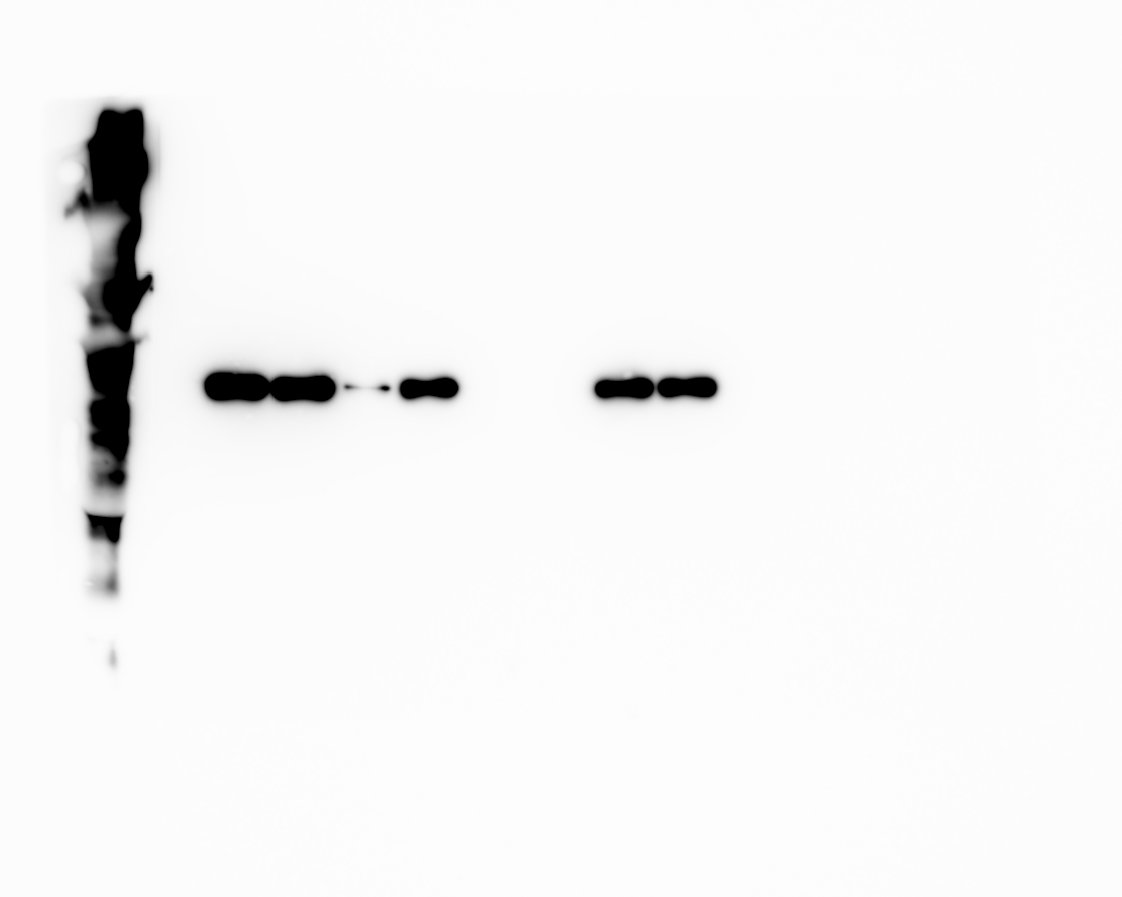

Supplement: Figure 11—source data 1. [file elife-85079-fig11-data1.zip › FIGURE 11 Source data/RAW data/Fig 11 panel F/Figure 11. panel F. Uso1 GHD-His6 pull-down. WB bound prey (+exp).tif]

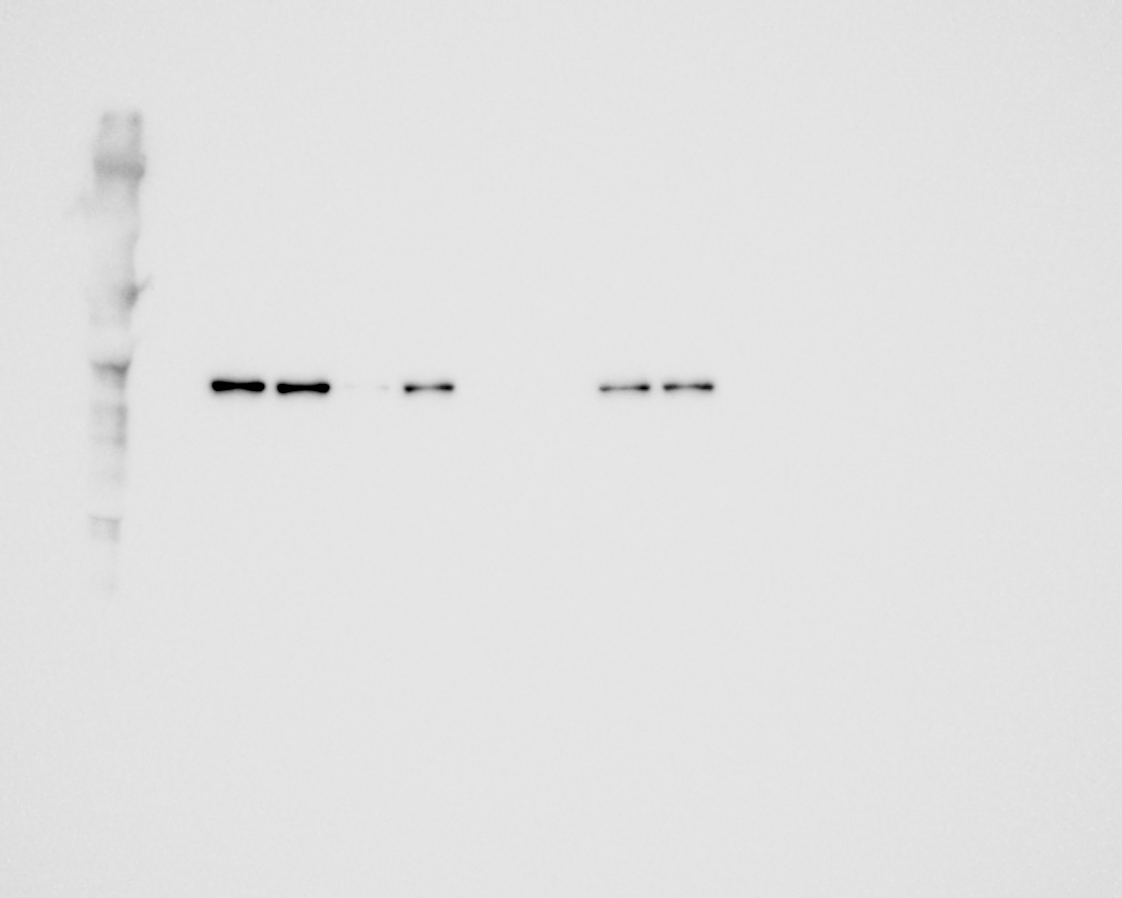

Supplement: Figure 11—source data 1. [file elife-85079-fig11-data1.zip › FIGURE 11 Source data/RAW data/Fig 11 panel F/Figure 11. panel F. Uso1 GHD-His6 pull-down. WB bound prey.tif]

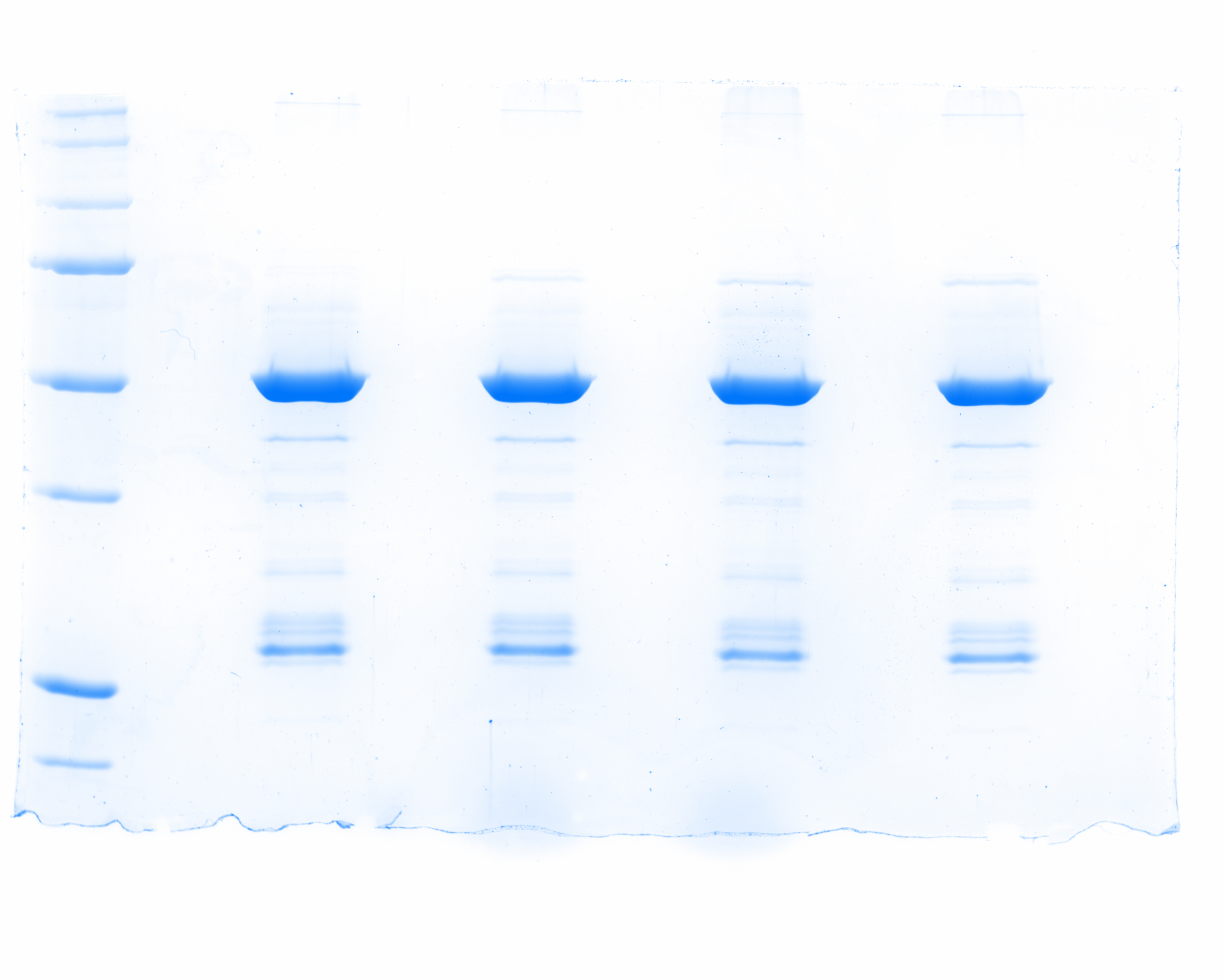

Supplement: Figure 11—source data 1. [file elife-85079-fig11-data1.zip › FIGURE 11 Source data/RAW data/Fig 11 panel G/Figure 11. panel G. GHDs-His pull-down Bos1-GST. Bos1-GST coomassie.tif]

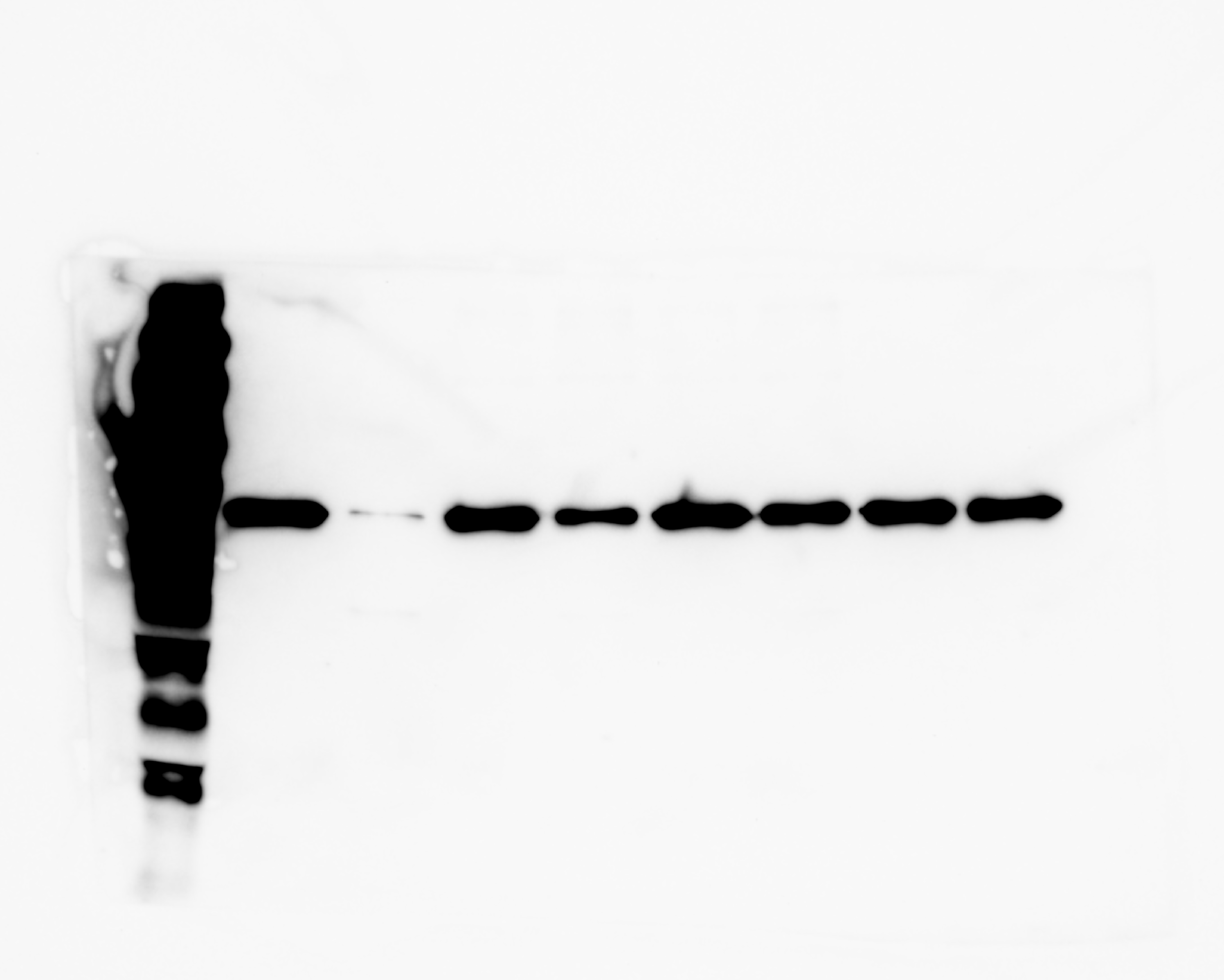

Supplement: Figure 11—source data 1. [file elife-85079-fig11-data1.zip › FIGURE 11 Source data/RAW data/Fig 11 panel G/Figure 11. panel G. GHDs-His pull-down Bos1-GST. WB bound prey (+exp).tif]

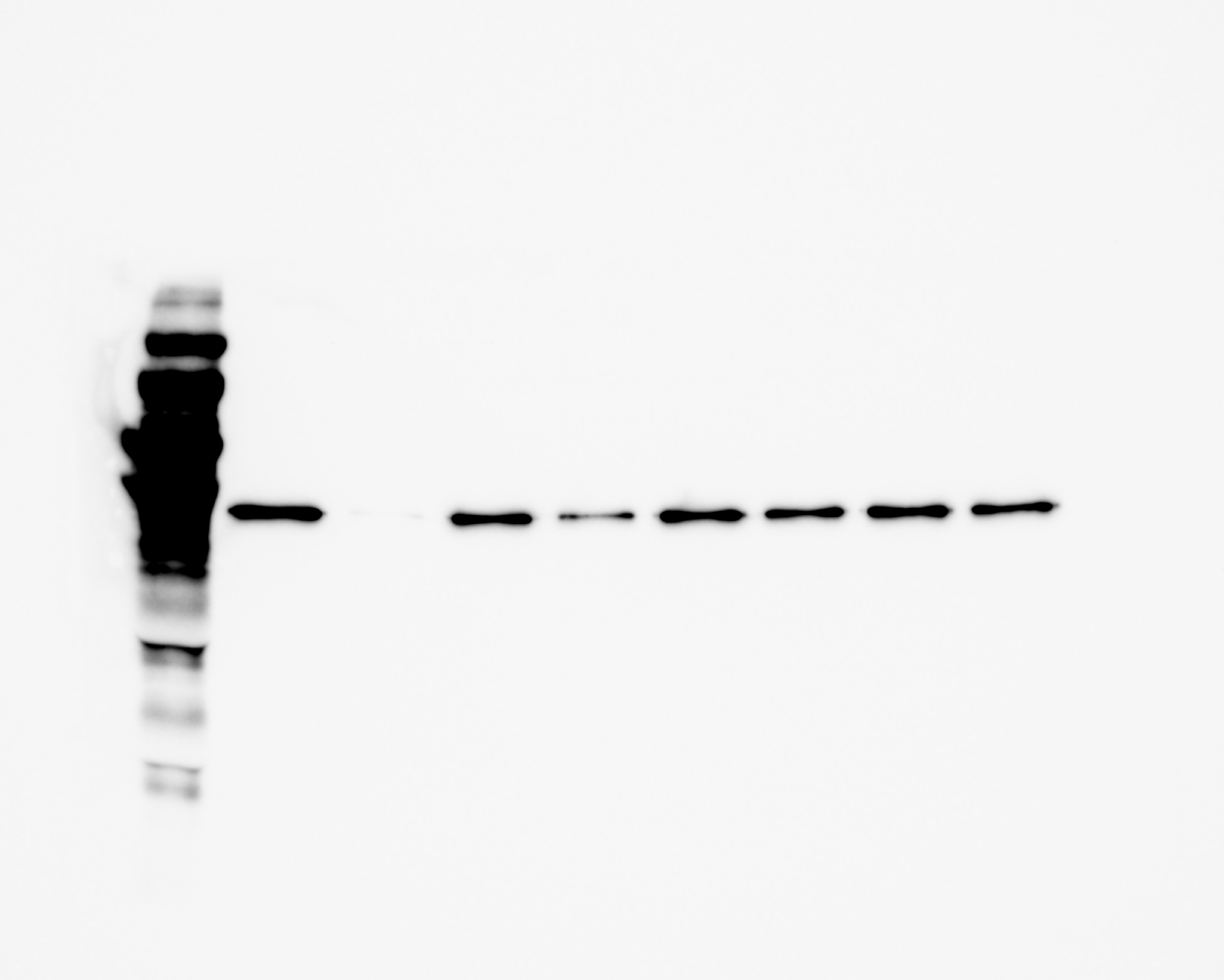

Supplement: Figure 11—source data 1. [file elife-85079-fig11-data1.zip › FIGURE 11 Source data/RAW data/Fig 11 panel G/Figure 11. panel G. GHDs-His pull-down Bos1-GST. WB bound prey (-exp).tif]

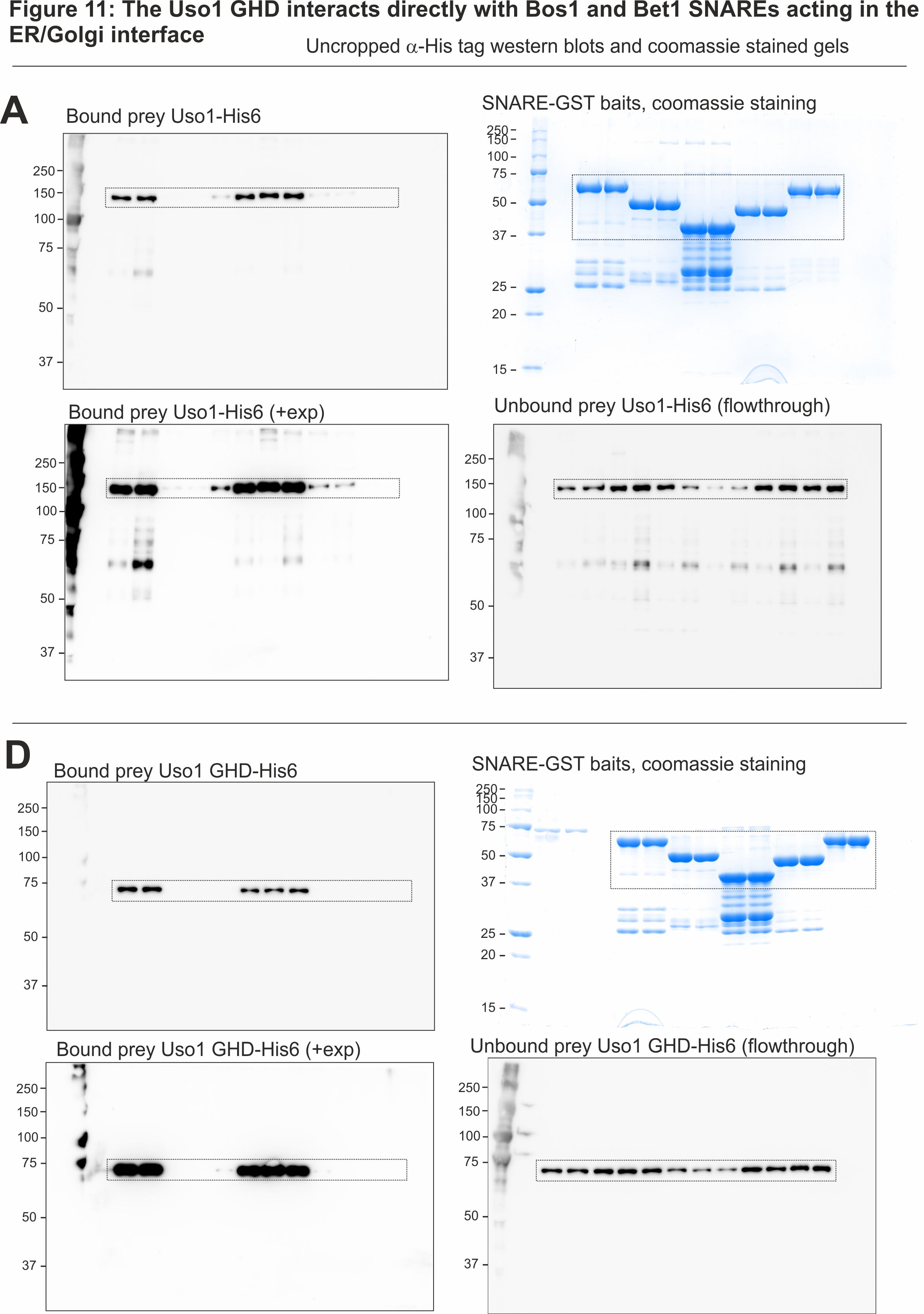

Supplement: Figure 11—source data 1. [file elife-85079-fig11-data1.zip › FIGURE 11 Source data/uncropped images Figure 11 (I).jpg]

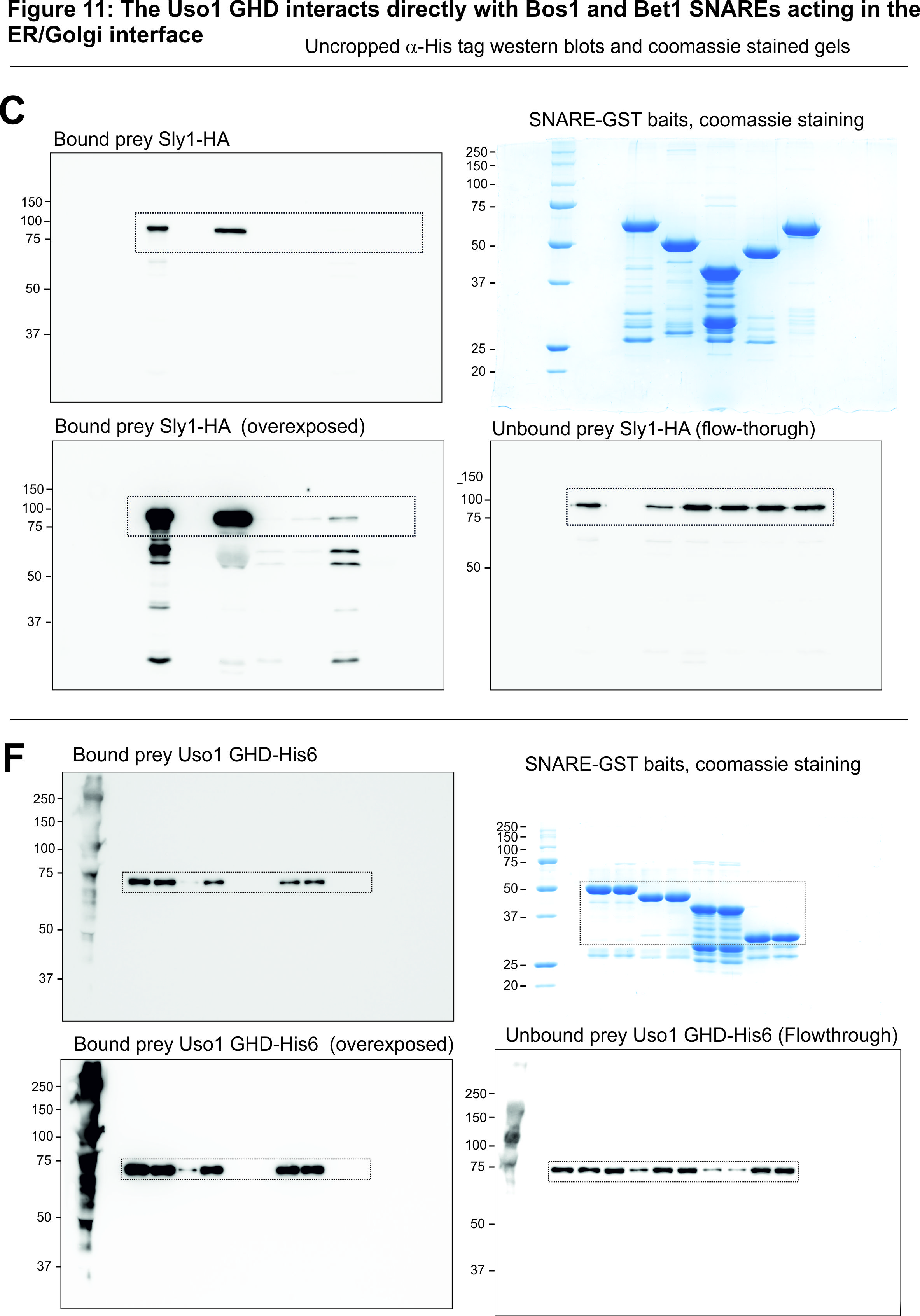

Supplement: Figure 11—source data 1. [file elife-85079-fig11-data1.zip › FIGURE 11 Source data/uncropped images Figure 11 (II).jpg]

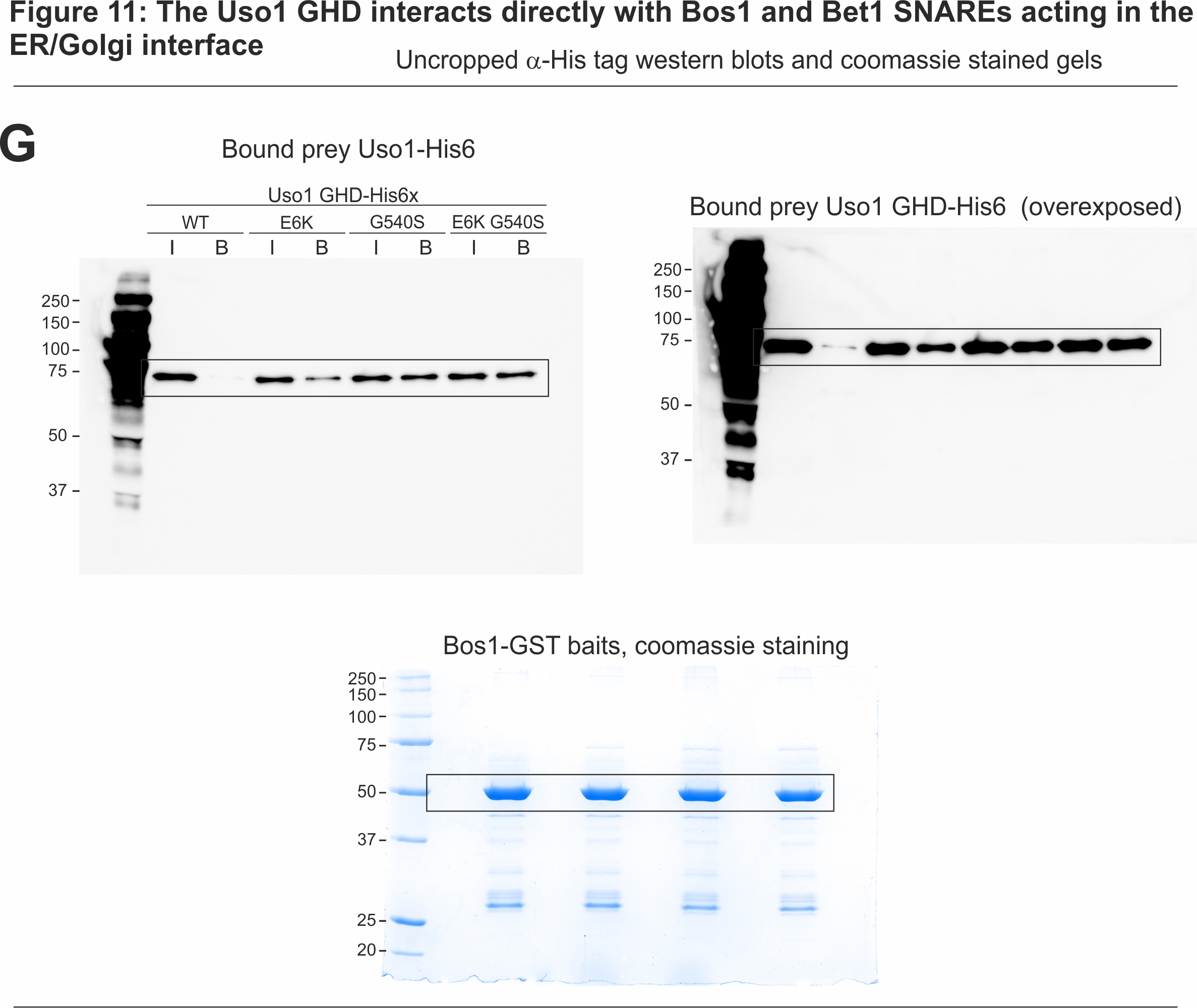

Supplement: Figure 11—source data 1. [file elife-85079-fig11-data1.zip › FIGURE 11 Source data/uncropped images Figure 11 (III).jpg]

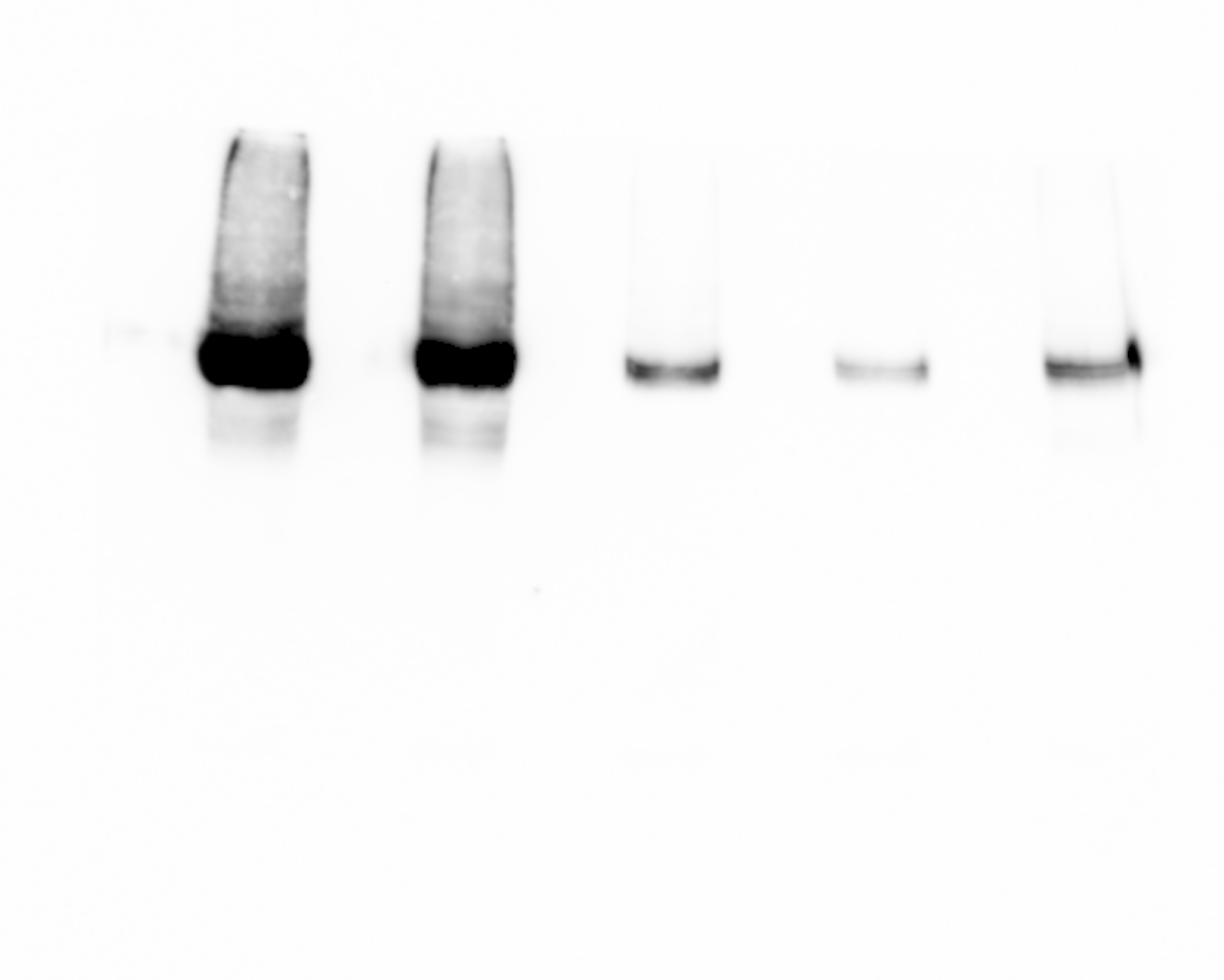

Supplement: Figure 13—figure supplement 2—source data 1. [file elife-85079-fig13-figsupp2-data1.zip › Figure 13 Fig suppl 2 Source data/raw data/Figure 13-figure supplement 2A. raw data. WB anti-HA tag.tif]

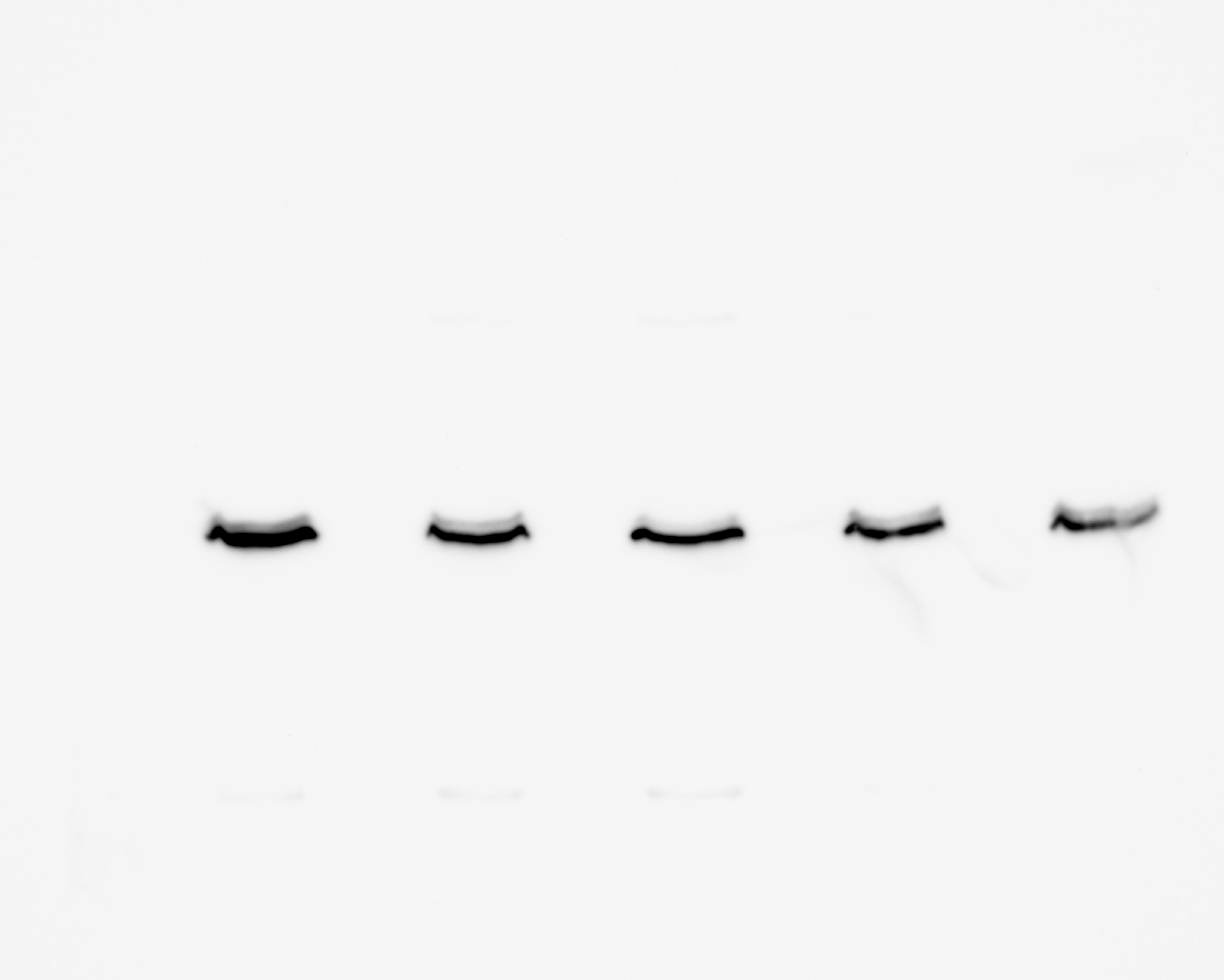

Supplement: Figure 13—figure supplement 2—source data 1. [file elife-85079-fig13-figsupp2-data1.zip › Figure 13 Fig suppl 2 Source data/raw data/Figure 13-figure supplement 2A. raw data. WB anti-tubulin.tif]

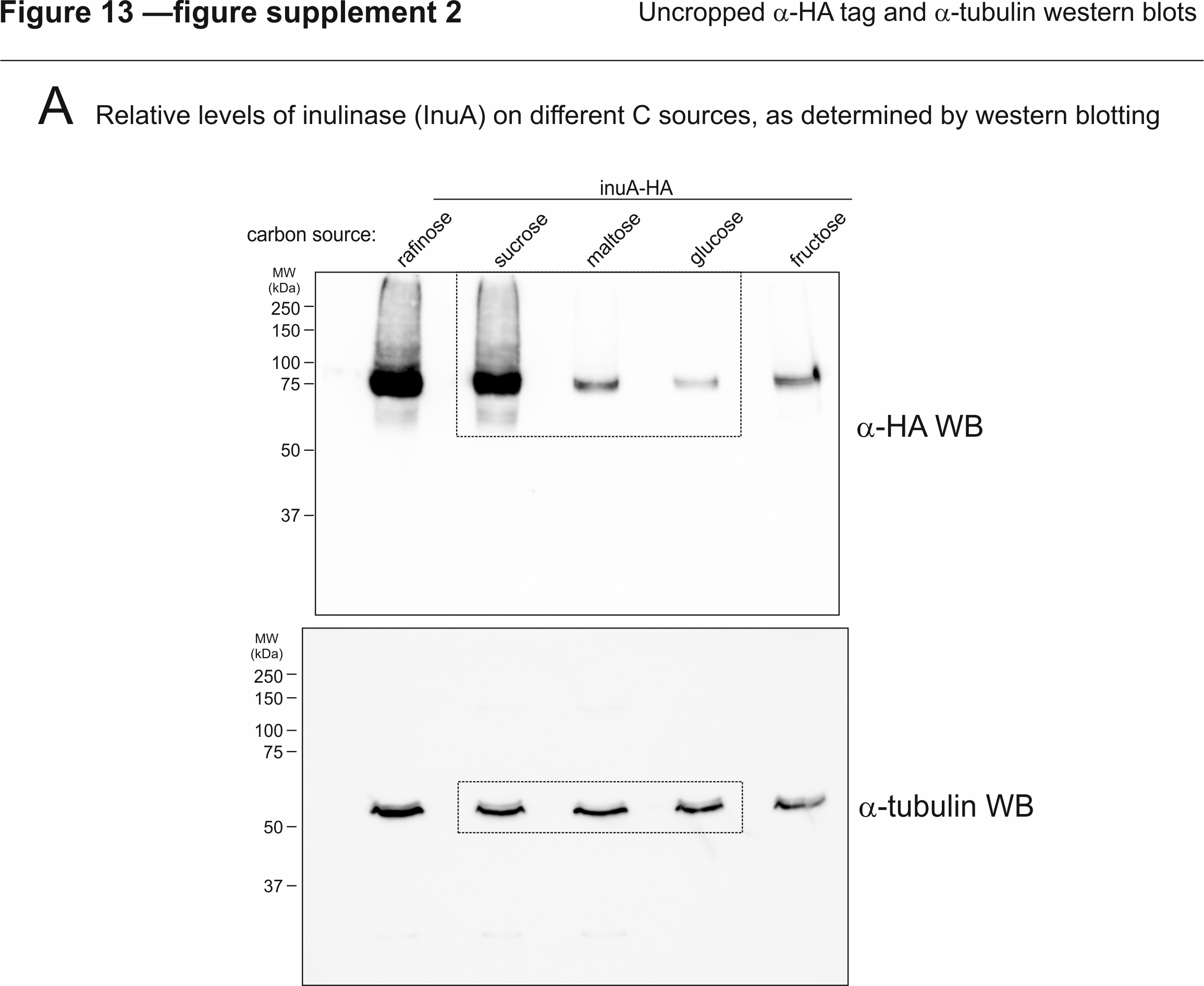

Supplement: Figure 13—figure supplement 2—source data 1. [file elife-85079-fig13-figsupp2-data1.zip › Figure 13 Fig suppl 2 Source data/uncropped images Figure 13-supplement 2 (v. 20).jpg]

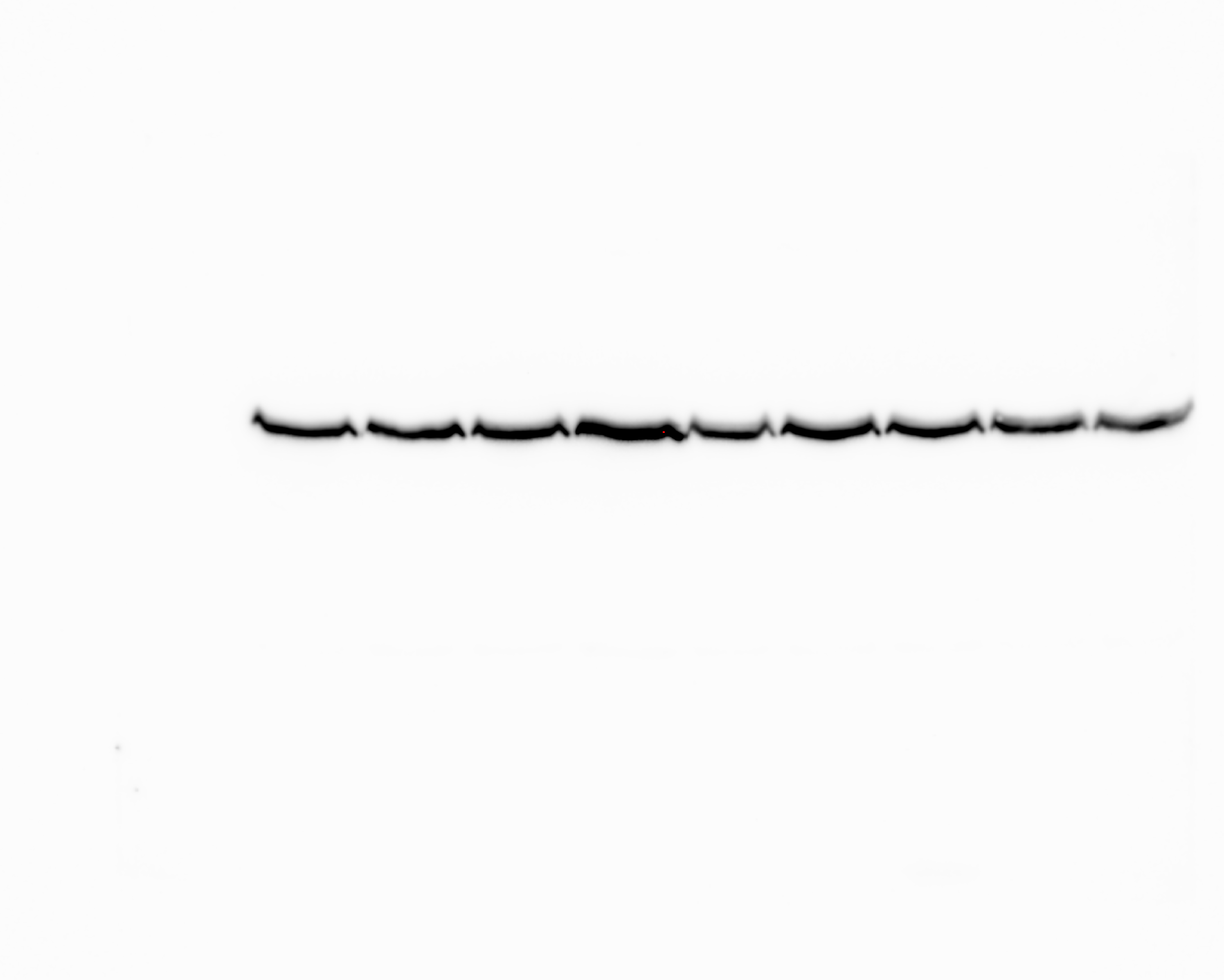

Supplement: Figure 14—source data 1. [file elife-85079-fig14-data1.zip › Figura 14 Source data/raw data/Figure 14A. raw data. WB anti-tubulin.tif]

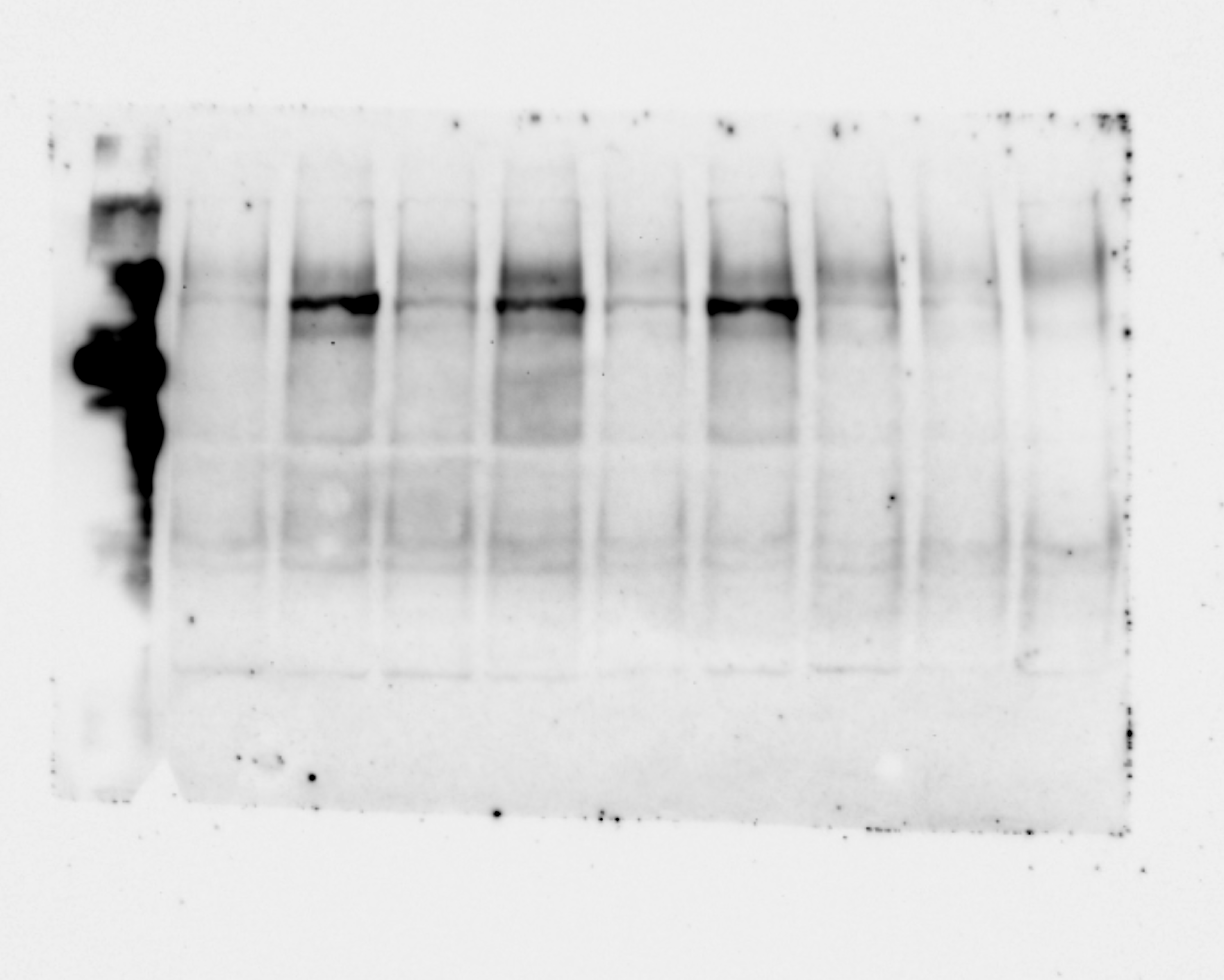

Supplement: Figure 14—source data 1. [file elife-85079-fig14-data1.zip › Figura 14 Source data/raw data/Figure 14A. raw data. WB anti-Uso1 GHD.tif]

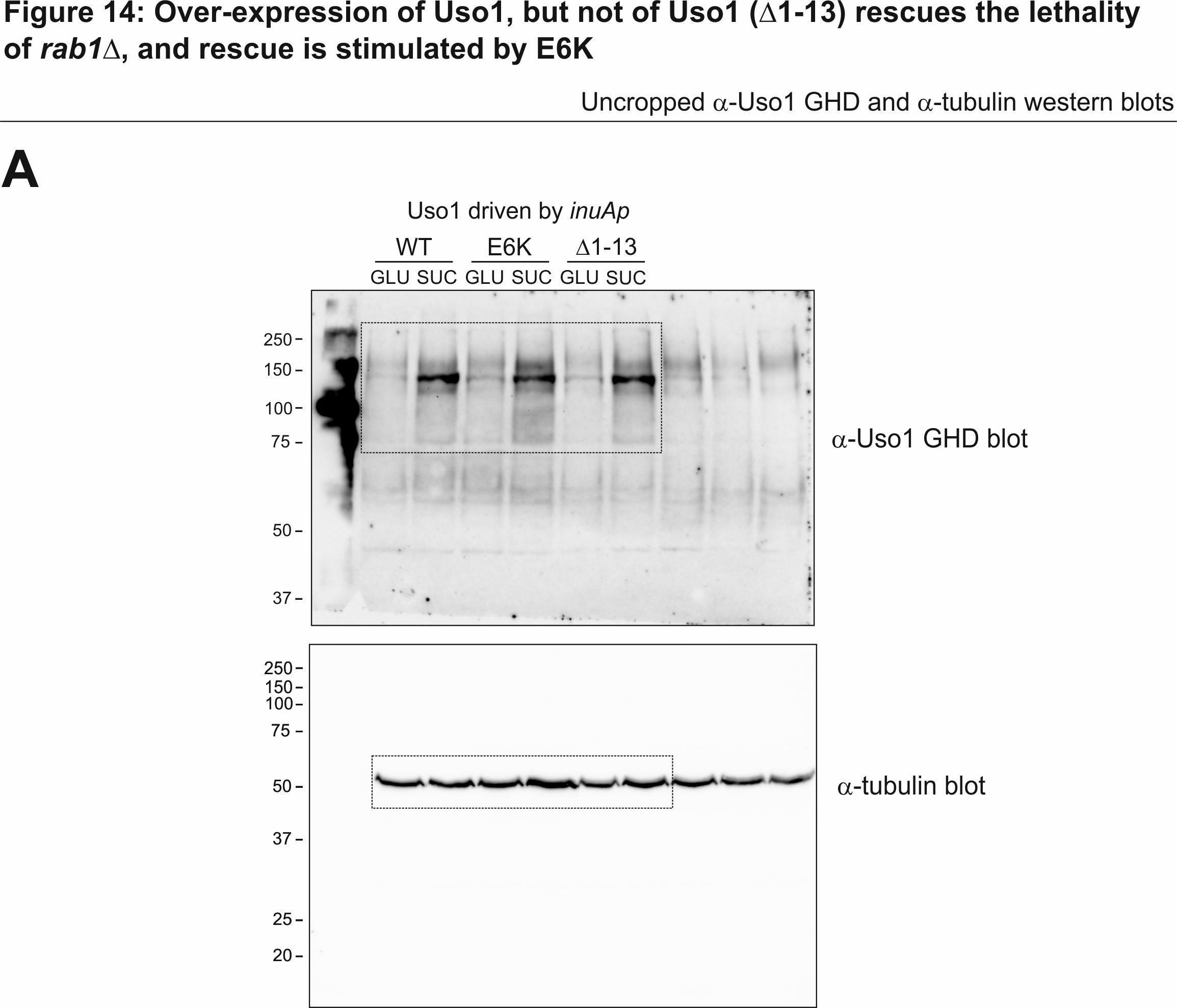

Supplement: Figure 14—source data 1. [file elife-85079-fig14-data1.zip › Figura 14 Source data/uncropped images Figure 14 (v. 20).jpg]
