# Supplementary material for: The Uso1 globular head interacts with SNAREs to maintain viability even in the absence of the coiled-coil domain
Source: eLife. 2023 May 30;12:e85079. doi: 10.7554/eLife.85079 (PMC10275640; doi:10.7554/eLife.85079)
Supplement: Supplementary file 2. [file elife-85079-supp2.docx]

**Supplementary table 1. *Aspergillus nidulans* strains used in this work.**

| Strain No. | Genotype |
| --- | --- |
| 5016 | *pyrG89; rab1^A136D^::pyrG^Af^; pyroA4 nkuA*Δ*::bar; uso1(E6K)* |
| 5018 | *pyrG89; rab1^A136D^::pyrGAf; pyroA4 nkuA*Δ*::bar; uso1(G540S)* |
| 5168 | *pyrG89; rab1^A136D^::pyrG^Af^; pyroA4 nkuA*Δ*::bar; uso1(E6K G540S)* |
| 5345 | *pyrG89; rabO^A136D^::pyrG^Af^; pyroA4 nkuA*Δ*::bar; su1* |
| 5346 | *pyrG89; rabO^A136D^::pyrG^Af^; pyroA4 nkuA*Δ*::bar; su85* |
| 5336 | *pyrG*89 *pabaA*1; *rab1*Δ*::pyrG^Af^ wA3*; *nkuA*Δ*::bar; usoA(E6K G540S)* |
| 6356 | *pyrG*89*? pabaA*1 *yA*2; *sec13-GA5x-mCherry-3´UTR-pyrG^Af^*; *nkuA*Δ*::bar?; uso1::gfp::pyrG^Af^* |
| 6359 | *pyrG*89*? pabaA*1 *yA*2; *nkuA*Δ*::bar?; uso1::gfp::pyrG^Af^* |
| 6590 | *pyrG*89 *pabaA*1; *wA*3; *nkuA*Δ*::bar; uso1(E6K G540S)::gfp::pyrG^Af^* |
| 6591 | *pyrG*89 *pabaA*1; *wA*3; *nkuA*Δ*::bar; uso1(E6K G540S)::gfp::pyrG^Af^* |
| 6872 | *pabaA*1*;nkuA*Δ*::bar; coy1*Δ*::riboB^Af^ riboB*2 |
| 6874 | *pabaA*1*;bug1*Δ*::riboB^Af^ nkuA*Δ*::bar; riboB*2 |
| 6876 | *pabaA*1*; nkuA*Δ*::bar; rud3Δ::riboB^Af^ riboB*2 |
| 6933 | *pabaA1 grh1*Δ*::riboB^Af^; nkuA*Δ*::bar; riboB*2 |
| 6995 | *pyrG*89?*; inoB*2 *pyroA*4*::[pyro^mut^-gpdAmini-mCherry-sed5] nkuA*Δ*::bar?; uso1::gfp::pyrG^Af^; niiA*4 |
| 6995 | *pyrG*89?*; inoB*2 *pyroA*4*::[pyro^mut^-gpdAmini-mCherry-sed5] nkuA*Δ*::bar?; uso1::gfp::pyrG^Af^; niiA*4 |
| 7001 | *pyrG*89?; wA3/4? *inoB*2 *pyroA*4*::[pyro^mut^-gpdAmini-mCherry-sed5] nkuA*Δ*::bar; uso1(E6K G540S)::gfp::pyrG^Af^* |
| 7001 | *pyrG*89?; wA3/4? *inoB*2 *pyroA*4*::[pyro^mut^-gpdAmini-mCherry-sed5] nkuA*Δ*::bar; uso1(E6K G540S)::gfp::pyrG^Af^* |
| 7060 | *pyrG*89*; pyroA*4 *nkuA*Δ*::bar; uso1::pyrG^Af^* |
| 7062 | *pyrG*89*; pyroA*4 *nkuA*Δ*::bar; uso1*Δ*CTR::pyrG^Af^* |
| 7100 | *pyrG*89*; wA*3*; sed5::HA3x::pyrG^Af^; nkuA*Δ*::bar; uso1(E6K G540S)::Stag::riboB^Af^ riboB*2 |
| 7102 | *pyrG*89 *pabaA*1*; wA*3*; sed5::HA3x::pyrG^Af^; nkuA*Δ*::bar; uso1::Stag::riboB^Af^ riboB*2 |
| 7126 | *pyrG*89*; wA*3*; nkuA*Δ*::bar; bet1::HA3x::pyrG^Af^; uso1(E6K G540S)::Stag::riboB^Af^ riboB*2 |
| 7128 | *pyrG*89 *pabaA*1*; wA*3*; nkuA*Δ*::bar; bet1::HA3x::pyrG^Af^; uso1::Stag::riboB^Af^ riboB*2 |
| 7130 | *pyrG*89 *bos1::HA3x::pyrG^Af^; wA*3*; nkuA*Δ*::bar; uso1(E6K G540S)::Stag::riboB^Af^ riboB*2 |
| 7132 | *pyrG*89 *bos1::HA3x::pyrG^Af^* *pabaA*1*; wA*3*; nkuA*Δ*::bar; uso1::Stag::riboB^Af^ riboB*2 |
| 7164 | *pyrG*89 *pabaA*1*; wA*3 *bapH::Stag::riboB^Af^; sed5::HA3x::pyrG^Af^; nkuA*Δ*::bar;riboB*2 |
| 7166 | *pyrG*89 *bos1::HA3x::pyrG^Af^* *pabaA*1*; wA*3 *bapH::Stag::riboB^Af^; nkuA*Δ*::bar; riboB*2 |
| 7166 | *pyrG*89 *bos1::HA3x::pyrG^Af^* *pabaA*1*; wA*3 *bapH::Stag::riboB^Af^; nkuA*Δ*::bar; riboB*2 |
| 7185 | *pyrG*89*; rab1^A136D^::riboB^Af^; pyroA*4 *nkuA*Δ*::bar; uso1::pyrG^Af^ riboB*2 |
| 7195 | *pyrG*89*; rab1^A136D^::riboB^Af^; pyroA*4 *nkuA*Δ*::bar; uso1*ΔCTR*::pyrG^Af^ riboB*2 |
| 7198 | *pyrG*89 *pabaA*1*; nkuA*Δ*::bar; uso1(E6K G540S)::pyrG^Af^* |
| 7200 | *pyrG*89 *pabaA*1*; nkuA*Δ*::bar; uso1(E6K G540S)*ΔCTR*::pyrG^Af^* |
| 7202 | *pyrG*89 *pabaA*1*; nkuA*Δ*::bar; uso1(E6K G540S)*ΔCC*::pyrG^Af^* |
| 7211 | *pyrG*89*; wA*3*; nkuA*Δ*::bar; sso1::HA3x::pyrG^Af^ ; uso1(E6K G540S)::Stag::riboB^Af^ riboB*2 |
| 7213 | *pyrG*89 *pabaA*1*; wA*3*; nkuA*Δ*::bar; sso1::HA3x::pyrG^Af^ ; uso1::Stag::riboB^Af^ riboB*2 |
| 7215 | *pyrG*89 *sec22::HA3x::pyrG^Af^; wA*3*; nkuA*Δ*::bar; uso1(E6K G540S)::Stag::riboB^Af^ riboB*2 |
| 7217 | *pyrG*89 *pabaA*1 *sec22::HA3x::pyrG^Af^; wA*3*; nkuA*Δ*::bar; uso1::Stag::riboB^Af^ riboB*2 |
| 7223 | *pyrG*89 *pabaA*1 *sec22::HA3x::pyrG^Af^; wA*3 *bapH::Stag::riboB^Af^; nkuA*Δ*::bar; riboB*2 |
| 7225 | *pyrG*89 *pabaA*1*; wA*3 *bapH::Stag::riboB^Af^; nkuA*Δ*::bar; sso1::HA3x::pyrG^Af^; riboB*2 |
| 7283 | *pyrG*89*; wA*3 *grh1::HA3x::pyrG^Af^; nkuA*Δ*::bar; uso1(E6K G540S)::Stag::riboB^Af^ riboB*2 |
| 7285 | *pyrG*89 *pabaA*1*; wA*3 *grh1::HA3x::pyrG^Af^; nkuA*Δ*::bar; uso1::Stag::riboB^Af^ riboB*2 |
| 7287 | *pyrG*89 *pabaA*1*; wA*3 *grh1::HA3x::pyrG^Af^ bapH::Stag::riboB^Af^; nkuA*Δ*::bar; riboB*2 |
| 7289 | *pyrG*89*; wA*3 *cog2::HA3x::pyrG^Af^; nkuA*Δ*::bar; uso1(E6K G540S)::Stag::riboB^Af^ riboB*2 |
| 7291 | *pyrG*89 *pabaA*1*; wA*3 *cog2::HA3x::pyrG^Af^; nkuA*Δ*::bar; uso1::Stag::riboB^Af^ riboB*2 |
| 7293 | *pyrG*89 *pabaA*1*; wA*3 *cog2::HA3x::pyrG^Af^ bapH::Stag::riboB^Af^; nkuA*Δ*::bar; riboB*2 |
| 7295 | *pyrG*89? *pabaA*1*; rab1^A136D^::pyrG^Af^; inoB*2 *pyroA*4 *nkuA*Δ*::bar; uso1::gfp::pyrG^Af^* |
| 7296 | *pyrG*89? *pabaA*1*; rab1^A136D^::pyrG^Af^; inoB*2 *pyroA*4 *nkuA*Δ*::bar; uso1::gfp::pyrG^Af^* |
| 7300 | *pyrG*89? *pabaA*1; *wA*3 *rab1^A136D^::pyrG^Af^; nkuA*Δ*::bar; uso1(E6K G540S)::gfp::pyrG^Af^* |
| 7301 | *pyrG*89?; *rab1^A136D^::pyrG^Af^; pyroA*4 *nkuA*Δ*::bar; uso1(E6K G540S)::gfp::pyrG^Af^* |
| 7308 | *pyrG*89*; rab1^A136D^::pyrG^Af^*; *bug1*Δ*::pyrG^Af^ pyroA*4 *nkuA*Δ*::bar; riboB*2 |
| 7309 | *pyrG*89*; rab1^A136D^::pyrG^Af^ grh1*Δ*::pyrG^Af^*; *pyroA*4 *nkuA*Δ*::bar; riboB*2 |
| 7314 | *pyrG*89*; wA*3*; nkuA*Δ*::bar; nsf::HA3x::pyrG^Af^; uso1(E6K G540S)::Stag::riboB^Af^ riboB*2 |
| 7316 | *pyrG*89 *pabaA*1*; wA*3*; nkuA*Δ*::bar; sec18::HA3x::pyrG^Af^; usoA::Stag::riboB^Af^ riboB*2 |
| 7318 | *pyrG*89 *pabaA*1*; wA*3 *slm2::Stag::riboB^Af^; nkuA*Δ*::bar; sec18::HA3x::pyrG^Af^; riboB*2 |
| 7320 | *pyrG*89*; wA*3*; nkuA*Δ*::bar;uso1(E6K G540S)::Stag::riboB^Af^ riboB*2 *βcop::HA3x::pyrG^Af^* |
| 7322 | *pyrG*89 *pabaA*1*; wA*3*; nkuA*Δ*::bar;uso1::Stag::riboB^Af^ riboB*2 *βcop::HA3x::pyrG^Af^* |
| 7324 | *pyrG*89 *pabaA*1*; wA*3 *bapH::Stag::riboB^Af^; nkuA*Δ*::bar;riboB*2 *βcop::HA3x::pyrG^Af^* |
| 7340 | *pyrG*89 *pabaA*1*; bug1*Δ*::pyrG^Af^ nkuA*Δ*::bar; uso1(E6K G540S)* |
| 7342 | *pyrG*89 *pabaA*1*; grh1*Δ*::pyrG^Af^*; *nkuA*Δ*::bar; uso1(E6K G540S)* |
| 7344 | *pabaA*1*; nkuA*Δ*::bar; uso1(E6K G540S)* |
| 7346 | *pyrG89? Sec7-tdTomato::pyrG^Af^; nkuA*Δ*::bar?; uso1::gfp::pyrG^Af^* |
| 7352 | *pyrG*89? *pabaA*1 *yA*2; *pyroA*4 *nkuA*Δ*::bar?; uso1::gfp::pyrG^Af^ sar1-*6*::pyrG^Af^* |
| 7354 | *pyrG*89?*;nkuA*Δ*::bar?; uso1(E6K G540S)::gfp::pyrG^Af^ sar1-*6*::pyrG^Af^* |
| 7359 | *pyrG*89*; wA*3*; bug1::HA3x::pyrG^Af^ nkuA*Δ*::bar; uso1(E6K G540S)::Stag::riboB^Af^ riboB*2 |
| 7361 | *pyrG*89 *pabaA*1*; wA*3*; bug1::HA3x::pyrG^Af^ nkuA*Δ*::bar; uso1::Stag::riboB^Af^ riboB*2 |
| 7363 | *pyrG*89 *pabaA*1*; wA*3 *slm2::Stag::riboB^Af^; bug1::HA3x::pyrG^Af^ nkuA*Δ*::bar; riboB*2 |
| 7365 | *pyrG*89*; wA*3*;nkuA*Δ*::bar; coy1::HA3x::pyrG^Af^ uso1(E6K G540S)::Stag::riboB^Af^ riboB*2 |
| 7367 | *pyrG*89 *pabaA*1*; wA*3*; nkuA*Δ*::bar; coy1::HA3x::pyrG^Af^ uso1::Stag::riboB^Af^ riboB*2 |
| 7369 | *pyrG*89 *pabaA*1*; wA*3 *bapH::Stag::riboB^Af^; nkuA*Δ*::bar; coy1::HA3x::pyrGAf riboB*2 |
| 7382 | *pabaA*1*; rab1^A136D^::riboB ^Af^; nkuA*Δ*::bar; uso1(E6K G540S) riboB*2 |
| 7383 | *pabaA*1*; grh1*Δ*::pyrG^Af^; rab1^A136D^::riboB ^Af^; nkuA*Δ*::bar; uso1(E6K G540S) riboB*2 |
| 7384 | *pabaA*1*; rabO^A136D^::riboB ^Af^; bug1*Δ*::pyrG^Af^ nkuA*Δ*::bar; uso1(E6K G540S) riboB*2 |
| 7482 | *pyrG*89*; wA*3 *rabO*Δ*::riboB^Af^; bug1*Δ*::pyrG^Af^ nkuA*Δ*::bar; uso1(E6K G540S) riboB*2 |
| 7485 | *pyrG*89*; wA*3 *rabO*Δ*::riboB^Af^ grh1*Δ*::pyrG^Af^; nkuA*Δ*::bar; uso1(E6K G540S) riboB*2 |
| 7500 | *pyrG*89 *pabaA*1*; wA*3; *inuA*Δ*::pyrG^Af^; nkuA*Δ*::bar* |
| 7505 | *pyrG*89*; wA*3 *rab1*Δ*::riboB^Af^; nkuA*Δ*::bar; rud3*Δ*::pyrG^Af^ uso1(E6K G540S) riboB*2 |
| 7511 | *pyrG*89*; wA*3 *rab1*Δ*::riboB^Af^; inuAΔ::pyrG^Af^; nkuA*Δ*::bar;uso1(E6K G540S) riboB*2 |
| 7521 | *pyrG*89*; wA*3 *rab1*Δ*::pyrG^Af^; pyroA*4 *nkuA*Δ*::bar; uso1(E6K)* |
| 7523 | *pyrG*89*; wA*3 *rab1*Δ*::pyrG^Af^; pyroA*4 *nkuA*Δ*::bar; uso1(G540S)* |
| 7596 | *pyrG*89? *pabaA*1*; rab1^A136D^::pyrG^Af^; pyroA*4 *nkuA*Δ*::bar; uso1(E6K G540S)*Δ*CTR::pyrG^Af^* |
| 7597 | *pyrG*89? *pabaA*1*; rab1^A136D^::pyrG^Af^; pyroA*4 *nkuA*Δ*::bar; uso1(E6K G540S)::pyrG^Af^* |
| 7875 | *pyrG*89 *pabaA*1*;* *gea1-tdTomato::pyrG^Af^;nkuA*Δ*::bar* |
| 8393 | *pyrG*89*; inuAp::uso1(*Δ*1-13)::riboB^Af^; pyroA*4 *nkuA*Δ*::bar; uds1::SPOTtag::pyrG^Af^ riboB*2 |
| 8395 | *pyrG*89*; inuAp::uso1(*Δ*1-13)::riboB^Af^; pyroA*4 *nkuA*Δ*::bar; uso1*Δ*::pyrG^Af^ riboB*2 |
| 8397 | *pyrG*89*; inuAp::uso1 wt::riboB^Af^; pyroA*4 *nkuA*Δ*::bar; uds1::SPOTtag::pyrG^Af^ riboB*2 |
| 8399 | *pyrG*89*; inuAp::uso1 wt::riboB^Af^; pyroA*4 *nkuA*Δ*::bar; uso1*Δ*::pyrG^Af^ riboB*2 |
| 8424 | *yA*2 *pyrG*89*?; pyroA*4 *nkuA*Δ*::bar?; pantoB100[pantoB*-gpdA^sh^::gfp::rer1]; uso1::mCherry::pyrG^Af^* |
| 8470 | *pyrG*89*? pabaA*1*; wA::bos1p::5'UTR::gfp::GA5x::bos1 cDNA::3'UTR::riboB^Af^;nkuA*Δ*::bar; riboB*2? *uso1::mCherry::pyrG^Af^* |
| 8497 | *pyrG*89 *pabaA*1*; rab1(A136D)::pyrG^Af^;nkuA*Δ*::bar?;uso1::GFP::pyrG^Af^* |
| 8500 | *pyrG*89 *pabaA*1*; rab1(A136D)::pyrG^Af^; nkuA*Δ*::bar?; gea1-tdTomato::pyrG^Af^* |
| 8501 | *pyrG89 pabaA*1*; rab1(A136D)::pyrG^Af^; nkuA*Δ*::bar?; gea1-tdTomato::pyrG^Af^  uso1::GFP::pyrG^Af^* |
| 8511 | *pyrG*89*; rab1*Δ*::pyrG^Af^ ; inuAp::uso1 wt::riboB^Af^; pyroA*4 *nkuA*Δ*::bar; riboB*2 |
| 8513 | *pyrG*89*; inuAp::uso1(E6K)::riboB^Af^; pyroA*4 *nkuA*Δ*::bar; uds1::SPOTtag::pyrG^Af^ riboB*2 |
| 8514 | *pyrG*89*; inuAp::uso1(E6K)::riboB^Af^; pyroA*4 *nkuA*Δ*::bar; uds1::SPOTtag::pyrG^Af^ riboB*2 |
| 8533 | *pyrG*89 *rab1*Δ*::pyrG^Af^; inuAp::uso1(E6K)::riboB^Af^; pyroA*4 *nkuA*Δ*::bar; riboB*2 |
| 8552 | *pyrG*89? *pabaA*1*; rab1(A136D)::pyrG^Af^; nkuA*Δ*::bar?; gea1-tdTomato::pyrG^Af^  uso1(E6K G540S)::GFP::pyrG^Af^* |
| 8556 | *pyrG*89 *pabaA*1*; nkuA*Δ*::bar?; gea1-tdTomato::pyrG^Af^  uso1(E6K G540S)::GFP::pyrG^Af^* |
