# Supplementary material for: The Uso1 globular head interacts with SNAREs to maintain viability even in the absence of the coiled-coil domain
Source: eLife. 2023 May 30;12:e85079. doi: 10.7554/eLife.85079 (PMC10275640; doi:10.7554/eLife.85079)
Supplement: Supplementary file 3. [file elife-85079-supp3.docx]

**Supplementary table 2. Oligonucleotides used for gene replacement or gene tagging cassette assembly and plasmid construction.**

| **Primer ID** | **Primer name** | **Sequence 5’ – 3’** |
| --- | --- | --- |

***uso1* (AN0706) knock-out cassette**

| VT57 | Fw prom usoA | CCGACGGCAATCATCAGGAG |
| --- | --- | --- |
| VT58 | Rv prom usoA | CGCAACGGGTCATTCGATATTTC |
| VT59 | Fw pyrG Afum cola | ACCATGAAATATCGAATGACCCGTTGCGGTAACGGCCGCCAGTGTGC |
| VT60 | Rv pyrG Afum cola | GCCTCCAGCATATAGAACGCGGTATATGCTGTCTGAGAGGAGGCACTG |
| VT61 | Fw usoA 3'UTR | CATATACCGCGTTCTATATGCTG |
| VT62 | Rv usoA 3'UTR | CCACACGGATTCCATAAACG |

***pyrG A. fumigatus*** (common fragment for fusion PCRs)

| IBP132 | Fw pyrGaf | ACCGGTCGCCTCAAACAATGC |
| --- | --- | --- |
| IBP133 | Rv pyrGaf | GTCTGAGAGGAGGCACTGATG |

***riboB A. fumigatus*** (common fragment for fusion PCRs)

| IBP230 | Fw riboB Af | GAGGCCGTTCAGGAGTCTGG |
| --- | --- | --- |
| IBP231 | Rv riboB Af | CAGAACGTTTGCGCTGCAGAAC |

***uso1::pyrGAf***

| VT7 | Fw ORF uso1 | CCGAGGTCGCTAATCTCAACAAC |
| --- | --- | --- |
| IBP294 | Rv fus Uso1END-pyrG | GCATTGTTTGAGGCGACCGGTTCACCCATGCTCCTCGTCAC |
| IBP292 | Fw fus pyrG-uso1_3' | CGCATCAGTGCCTCCTCTCAGACTTGAATGACTTTCTAGATACATATAC |
| VT12 | Rv uso1-3'UTR | CGAGTAGGGCATGACACGGAAG |

***uso1*Δ*CTR::pyrGAf***

| VT7 | Fw ORF uso1 | CCGAGGTCGCTAATCTCAACAAC |
| --- | --- | --- |
| IBP295 | Rv fus Uso1CTR-pyrG | CATTGTTTGAGGCGACCGGTTCAGAGCGCCTTCTCGAGC |
| IBP292 | Fw fus pyrG-uso1_3' | CGCATCAGTGCCTCCTCTCAGACTTGAATGACTTTCTAGATACATATAC |
| VT12 | Rv uso1-3'UTR | CGAGTAGGGCATGACACGGAAG |

***uso1*Δ*CCD::pyrGAf***

| IBP156 | Fw G540S | CGAGATACCTAACATACTGACTG |
| --- | --- | --- |
| IBP293 | Rv fus uso1_HD-pyrG | CATTGTTTGAGGCGACCGGTTCATTCTGGCTCACAATCAATCGCC |
| IBP292 | Fw fus pyrG-uso1_3' | CGCATCAGTGCCTCCTCTCAGACTTGAATGACTTTCTAGATACATATAC |
| VT12 | Rv uso1-3'UTR | CGAGTAGGGCATGACACGGAAG |

***bug1* (AN7680) knock-out cassettes**

| IBP233 | Fw upstream bug1 | GAGCAAGCGTGCTAAATACTGG |
| --- | --- | --- |
| IBP234 | Rv upstream bug1 | CAGACTCCTGAACGGCCTCTGTGCTAGCAATGAAATTAGGGAG |
| IBP235 | Fw downstream bug1 | TCTGCAGCGCAAACGTTCTGACCTTGCCCTCTTAATTTCTCAC |
| IBP236 | Rv downstream bug1 | GGTCCACGGTTTGAAACATGG |
| IBP384 | Rv fus bug1-pyrGAf | GCATTGTTTGAGGCGACCGGTTGTGCTAGCAATGAAATTAGGGAG |
| IBP385 | Fw fus bug1-pyrGAf | CATCAGTGCCTCCTCTCAGACACCTTGCCCTCTTAATTTCTCAC |

***coy1* (AN0762) knock-out cassettes**

| IBP239 | Fw upstream coy1 | CGGGACTCACTCCAATACTCC |
| --- | --- | --- |
| IBP240 | Rv upstream coy1 | CCAGACTCCTGAACGGCCTCCCGGCTCTCGTCGCTGCGT |
| IBP241 | Fw downstream coy1 | GTTCTGCAGCGCAAACGTTCTGAGTATTAGGGCGTTAAGGAGTTC |
| IBP242 | Rv downstream coy1 | GGAGGATGCCAGGTAGTTCG |
| IBP386 | Rv fus coy1-pyrGAf | GCATTGTTTGAGGCGACCGGTCCGGCTCTCGTCGCTGCGT |
| IBP387 | Fw fus coy1-pyrGAf | CATCAGTGCCTCCTCTCAGACAGTATTAGGGCGTTAAGGAGTTC |

***grh1* knock-out cassettes**

| MP20 | Fw grhA upstream | ATGGTTCGCATTAGCGCTTCTG |
| --- | --- | --- |
| IBP229 | Rv upstream grhA | CAGACTCCTGAACGGCCTCTGTGAAATCTGTTTAGTTGTCAAAC |
| IBP232 | Fw downstream grhA | GTTCTGCAGCGCAAACGTTCTGGAGATTTGTTTACGACTTGCAGC |
| MP18 | Rv grhA downstream | GCCATAGCAGCAACCACAACG |
| IBP383 | Rv fus grh1-pyrGAf | GCATTGTTTGAGGCGACCGGTTGTGAAATCTGTTTAGTTGTCAAAC |
| IBP384 | Fw fus grh1-pyrGAf | CATCAGTGCCTCCTCTCAGACGAGATTTGTTTACGACTTGCAGC |

***rud3* (AN10186) knock-out cassettes**

| IBP243 | Fw upstream rud3 | CAGCCCACTGGTCTACGTCC |
| --- | --- | --- |
| IBP244 | Rv upstream rud3 | CCAGACTCCTGAACGGCCTCCTTCAAAGGAGAGCTCTGTGG |
| IBP245 | Fw downstream rud3 | GTTCTGCAGCGCAAACGTTCTGGCTGGAGATATACAGCAACTCC |
| IBP246 | Rv downstream rud3 | CAGGCGTTAGTCTACCAACTG |
| IBP388 | Rv fus rud3-pyrGAf | GCATTGTTTGAGGCGACCGGTCTTCAAAGGAGAGCTCTGTGG |
| IBP389 | Fw fus rud3-pyrGAf | CATCAGTGCCTCCTCTCAGACGCTGGAGATATACAGCAACTCC |

**HA3 tagging of potential Uso1 interactors: co-precipitation experiments**

***uso1-Stag::riboB^Af^***

| VT7 | Fw uso1-genom | CCGAGGTCGCTAATCTCAACAAC |
| --- | --- | --- |
| IBP334 | Rv FUS Stag-riboBAf | CAGACTCCTGAACGGCCTCTTAGCTGTCCATGTGCTGGCG |
| IBP335 | Fw FUS riboBAf uso1 | CTGCAGCGCAAACGTTCTGCCGCGTTCTATATGCTGGAGG |
| VT12 | Rv uso1-3'UTR | CGAGTAGGGCATGACACGGAAG |

***uso1-HA3::pyrG^Af^***

| VT7 | Fw uso1-genom | CCGAGGTCGCTAATCTCAACAAC |
| --- | --- | --- |
| VT8 | Rv uso1-genom | CCCATGCTCCTCGTCACCCTC |
| VT9 | Fw uso1-HA fus | GACGAGGGTGACGAGGAGCATGGGGGAGCTGGTGCAGGCGCTGGAG |
| VT10 | Rv pyrG-uso1 fus | GCCTCCAGCATATAGAACGCGGGTCTGAGAGGAGGCACTGATGCGT |
| VT11 | Fw uso1-3'UTR | CCGCGTTCTATATGCTGGAGGC |
| VT12 | Rv uso1-3'UTR | CGAGTAGGGCATGACACGGAAG |

**GA5x-HA3-pyrG *A. fumigatus***

| IBP297 | Fw GA5x | GGAGCTGGTGCAGGCGCTG |
| --- | --- | --- |
| IBP133 | Rv pyrGaf | GTCTGAGAGGAGGCACTGATG |

**Sed5-HA3**

| MP66 |  | GGAGCACAATGATAATGTACGTTG |
| --- | --- | --- |
| IBP298 | Rv fus. sedV-GA5x | CAGCGCCTGCACCAGCTCCTCCTGAGATCAATACCCAGAGG |
| IBP299 | Fw fus. pyrG-sed5 3’ | CATCAGTGCCTCCTCTCAGACGCACATACTACTTGTTTTAATAATGT |
| MP15 |  | AAGTGCTAGAGCGACTCCAATCC |

**Bos1-HA3**

| IBP340 | Fw ORF Bos1 | CCTGCGACAACTCCTGCAGC |
| --- | --- | --- |
| IBP341 | Rv FUS Bos1-HA | CAGCGCCTGCACCAGCTCCTCTCAAGAAGTGCAATACTGCC |
| IBP342 | Fw FUS Bos1 pyrGaf | CATCAGTGCCTCCTCTCAGACGCTTGTACTGGCGGCTGTCG |
| IBP343 | Rv downstream Bos1 | GCTGTTAGTGTTGATGTGG |

**Bet1-HA3**

| IBP336 | Fw ORF Bet1 | CCTCTTCTAACCTCCTTTCTCC |
| --- | --- | --- |
| IBP337 | Rv FUS Bet1-HA | CAGCGCCTGCACCAGCTCCAGTGATCCACACATAGAAGAAAAG |
| IBP338 | Fw FUS Bet1 pyrGaf | CATCAGTGCCTCCTCTCAGACAGGCCTTGCCTTACCCAAGC |
| IBP339 | Rv downstream Bet1 | GGTGAGTTAGTACGACCACTG |

**Sec22-HA3**

| IBP373 | Fw ORF Sec22 | GGATAGCCTATAGTGGTGATAC |
| --- | --- | --- |
| IBP374 | Rv FUS Sec22-HA | CAGCGCCTGCACCAGCTCCGAAGAAGCGCCACCAAATGAG |
| IBP375 | Fw FUS Sec22 pyrGaf | CATCAGTGCCTCCTCTCAGACTCTCATTTCAGCGCCCAGCC |
| IBP376 | Rv downstream Sec22 | CGACCGGTCATTTAACCTCTC |

**Sso1-HA3**

| IBP390 | Fw ORF sso1 | CTACTACACAGACTAACCACGC |
| --- | --- | --- |
| IBP391 | Rv FUS sso1-GA5x | CAGCGCCTGCACCAGCTCCTCTCGTCTGCGTAACAGCAAC |
| IBP392 | Fw FUS pyrGAf-sso1 | CATCAGTGCCTCCTCTCAGACAGGAAAAAAGATCTCTTGGCTGG |
| IBP393 | Rv downstream sso1 | CTCTCTATCAGACGCCATAGTC |

**COG2-HA3**

| IBP400 | Fw ORF cog2 | CGATCCAGCGACGTATCTCTC |
| --- | --- | --- |
| IBP401 | Rv FUS ORFcog2-GA5x | CAGCGCCTGCACCAGCTCCTATAGTCAAGTCTTTTAGGGCGG |
| IBP402 | Fw FUS pyrGAf-cog2 | CATCAGTGCCTCCTCTCAGACAATTGCGTTTGTTATTGCATAGCG |
| IBP403 | Rv downstream cog2 | CTTCCGGACCTTGCTGCAGC |

**Sec18-HA3**

| IBP419 | Fw sec18 ORF | GCTCTTGATGAAATCCAGCCAG |
| --- | --- | --- |
| IBP420 | Rv FUS sec18-GA5x | CAGCGCCTGCACCAGCTCCAACTGCCCTTCTAGTAGTCAATC |
| IBP421 | Fw FUS pyrGaf-sec18 | CATCAGTGCCTCCTCTCAGACTCGGCGCTTAGAGGTCTCTAAG |
| IBP422 | Rv downstream sec18 | CTCCGTAAAGATCTAGCTTTCC |

**βCOP-HA3**

| IBP427 | Fw βCOP ORF | CAAGGCTACAGGTGGTGACTC |
| --- | --- | --- |
| IBP428 | Rv FUS βCOP-GA5x | CAGCGCCTGCACCAGCTCCCGCCGCAGAGGCTTTCAGAC |
| IBP429 | Fw FUS pyrGaf-βCOP | CATCAGTGCCTCCTCTCAGACTCTATATCCGAATAGTGATCATAC |
| IBP430 | Rv downstream βCOP | GACCATTCCACAACATGTGCG |

**Coy1-HA3**

| IBP423 | Fw coy1 ORF | CCACTCTAGAGAATGATCTTCTC |
| --- | --- | --- |
| IBP424 | Rv FUS coy1-GA5x | CAGCGCCTGCACCAGCTCCCCCAGCGTGATTGAATCCCTC |
| IBP425 | Fw FUS pyrGaf-coy1 | CATCAGTGCCTCCTCTCAGACTCGGTTGTATTTTTAGTATTAGGG |
| IBP426 | Rv downstream coy1 | CACGTTGTGCCTTCCCTAATC |

**Bug1-HA3**

| IBP437 | Fw ORF bug1 | GCTAAACTACGCGAAAGCGAG |
| --- | --- | --- |
| IBP438 | Rv fus bug1-GA5x | CAGCGCCTGCACCAGCTCCTATCTCAAAGATTTCCCCCATGC |
| IBP385 | Fw fus bug1-pyrGAf | CATCAGTGCCTCCTCTCAGACACCTTGCCCTCTTAATTTCTCAC |
| IBP236 | Rv downstream bug1 | GGTCCACGGTTTGAAACATGG |

**Grh1-HA3**

|  | Fw GSP1-Grh1 | CGAGTTACCTTTGCAGTATGGAGCG |
| --- | --- | --- |
| IBP399 | Rv fus grh1-GA5x | CAGCGCCTGCACCAGCTCCTCCGGATTCCTTTTGCTCCTG |
| IBP383 | Rv fus grh1-pyrGAf | GCATTGTTTGAGGCGACCGGTTGTGAAATCTGTTTAGTTGTCAAAC |
| MP18 | Rv grhA downstream | GCCATAGCAGCAACCACAACG |

**pET21b Uso1 His6x-tagging expression plasmids**

**pET21b-Uso1-His6x and pET21b-Uso1(E6K/G540S)-His6x**

| IBP176 | Fw usoA head (NdeI) | ATATATCATATGTTTCGAATCCTCGAATCAC |
| --- | --- | --- |
| IBP214 | Rv usoA full (NotI) | ATATATGCGGCCGCCCCATGCTCCTCGTCACCC |
| IBP274 | Fw usoA E6K (NdeI) | ATATATCATATGTTTCGAATCCTCAAATCAC |

**pET21b-Uso1ΔCTR-His6x and pET21b-Uso1(E6K/G540S)ΔCTR-His6x**

| IBP176 | Fw usoA head (NdeI) | ATATATCATATGTTTCGAATCCTCGAATCAC |
| --- | --- | --- |
| IBP296 | Rv uso1 CTR (NotI) | ATATATGCGGCCGCGAGCGCCTTCTCGAGCGC |

**pET21b-Uso1GHD-His6x and pET21b-Uso1(E6K/G540S)GHD-His6x**

| IBP176 | Fw uso1 head (NdeI) | ATATATCATATGTTTCGAATCCTCGAATCAC |
| --- | --- | --- |
| IBP177 | Rv uso1 head (XhoI) | ATATATCTCGAGTTCTGGCTCACAATCAATCGC |
| IBP274 | Fw uso1 E6K (NdeI) | ATATATCATATGTTTCGAATCCTCAAATCAC |

**pET21b-Uso1 CCD-His6x**

| IBP271 | Fw uso1-CCtail (NdeI) | ATATATCATATGATCGAAATTTCCGTGATTACAAAC |
| --- | --- | --- |
| IBP214 | Rv uso1 full (NotI) | ATATATGCGGCCGCCCCATGCTCCTCGTCACCCc |

**TNT^®^ expression plasmids**

**pSP64-Sly1-HA**

| IBP487 | Fw NsiI-sly1 PCR A | ATATATATGCATATGGCATCACAACCAATGTCTC |
| --- | --- | --- |
| IBP488 | Rv Sly1 PCR A | GCTTTGTCGCGATAAGCTCAG |
| IBP489 | Fw Sly1 PCR B | CCATACCGATCATACGCTGTC |
| IBP490 | Rv Sly1-XmaI PCR B | ATATATCCCGGGACCTTCCCTCCCTAATTTCGC |
| IBP453 | Fw XmaI GA5x | ATATATCCCGGGGGAGCTGGTGCAGGCGCTG |
| IBP454 | Rv SacI HA3x | ATATATGAGCTCTTACTGAGCAGCGTAATCTGGAAC |

**pET21b SNARE cytosolic domain-GA5x-GST expression plasmids**

**GA5x-GST**

| IBP406 | Fw FUS GA5x+GST | GTGCAGGCGCTGGAGCCGGTGCCTCCCCTATACTAGGTTATTGGAA |
| --- | --- | --- |
| IBP407 | Rv *Sal*I GST | ATATATGTCGACTCTATCAGGATCCACGCGGAAC |

**Sed5 (Qa SNARE) cytoplasmic domain: residues 1–322**

| IBP404 | Fw *Nde*I SedV cDNA | ATATATCATATGACCGGGCCTACGATACAG |
| --- | --- | --- |
| IBP405 | Rv FUS SedV-CD+GA5x | GCTCCAGCGCCTGCACCAGCTCCTCGGTTTCCCGATACTCGTG |

**Bos1 (Qb SNARE) cytoplasmic domain: residues 1–219**

| IBP410 | Fw NdeI Bos1 cDNA | ATATATCATATGAATTCACTGTTCAACTCCGC |
| --- | --- | --- |
| IBP411 | Rv FUS Bos1-CD+GA5x | GCTCCAGCGCCTGCACCAGCTCCTTTGTCCTGCTTCGCTCGTCG |

**Bet1 (Qc SNARE) cytoplasmic domain: residues 1–71**

| IBP408 | Fw NdeI Bet1 cDNA | ATATATCATATGGCTTCAAGATTCTCTCGGTC |
| --- | --- | --- |
| IBP409 | Rv FUS Bet1-CD+GA5x | GCTCCAGCGCCTGCACCAGCTCCCGTGCGCTCTGCCATGCGC |

**Sec22 (R SNARE) cytoplasmic domain: residues 1–198**

| IBP412 | Fw NheI Sec22 cDNA | ATATATGCTAGCATGGTTAAATCAACCCAGATTGC |
| --- | --- | --- |
| IBP413 | Rv FUS Sec22-CD+GA5x | GCTCCAGCGCCTGCACCAGCTCCAGGCCCATACTGTTTTACTAGC |

**Sso1 (Qa SNARE) cytoplasmic domain: residues 1–271**

| IBP414 | Fw NdeI Sso1 cDNA | ATATATCATATGAGTTACGGACAAAGTTACAAC |
| --- | --- | --- |
| IBP415 | Rv FUS Sso1-CD+GA5x | GCTCCAGCGCCTGCACCAGCTCCTTTCCACCTCCGGGCCTTGCG |

**Gos1** **(Qb SNARE) cytoplasmic domain: residues 1–208**

| IBP475 | Fw NdeI Gos1 cDNA | ATATATCATATGGCGTCCTCTACTGGTGC |
| --- | --- | --- |
| IBP476 | Rv FUS Gos1-CD+GA5x | GCTCCAGCGCCTGCACCAGCTCCATCTCTCCTCCTTTTATTCCC |

**Sft1 (Qc SNARE) cytoplasmic domain: residues 1–73**

| IBP477 | Fw NdeI Sft1 cDNA | ATATATCATATGGCGGACGCCTACGAACG |
| --- | --- | --- |
| IBP488 | Rv FUS Sft1-CD+GA5x | GCTCCAGCGCCTGCACCAGCTCCCGTGTCACCCTGTCTCGCC |

**InuA promoter-driven expression of Uso1 GHD in *Aspergillus nidulans***

**Gene replacement cassette assembled by 4-way fusion PCR:**

**(1) inuA promoter**

| MAPS254 | Fw upstream inuA | GTGGAGGCCACTCTCGGAAAC |
| --- | --- | --- |
| MHG267 | Rv upstream inuA | GCGGTCAGCGACATCACCAAA |

**(2) cDNA sequence encoding Uso1 wt or mutant (E6K, G540S) GHD (residues 1 – 659)**

| IBP484 | Fw inuAp-uso1 wt FUS | GCGGTCAGCGACATCACCAAAATGTTTCGAATCCTCGAATCACAG |
| --- | --- | --- |
| IBP485 | Fw inuAp-uso1 E6K FUS | GCGGTCAGCGACATCACCAAAATGTTTCGAATCCTCAAATCACAG |
| IBP486 | Rv uso1deltaCCD-riboB FUS | CCAGACTCCTGAACGGCCTCTCATTCTGGCTCACAATCAATCGCC |

**(3) *Aspergillus fumigatus riboB* gene as selection marker**

| IBP230 | Fw riboB Af | GAGGCCGTTCAGGAGTCTGG |
| --- | --- | --- |
| IBP231 | Rv riboB Af | CAGAACGTTTGCGCTGCAGAAC |

**(4) *inuA* gene 3’-flanking region**

| IBP279 | Fw Fus-inuA3-riboB | GTTCTGCAGCGCAAACGTTCTGGGATCTAGCTAGATGTTTTGTTG |
| --- | --- | --- |
| SF3 | downstream inuA | CAGCAGTCAAGCAATACCAAGC |

**InuA promoter-driven expression of full-length and Δ1-13 mutant Uso1 in *Aspergillus nidulans***

**Gene replacement cassette assembled by 4-way fusion PCR:**

**(1) inuA promoter**

| MAPS254 | Fw upstream inuA | GTGGAGGCCACTCTCGGAAAC |
| --- | --- | --- |
| MHG267 | Rv upstream inuA | GCGGTCAGCGACATCACCAAA |

**(2) cDNA sequence encoding full length wt Uso1, E6K mutant or Δ1-13 mutant lacking the N-terminal helix**

| IBP484 | Fw inuAp-uso1 wt FUS | GCGGTCAGCGACATCACCAAAATGTTTCGAATCCTCGAATCACAG |
| --- | --- | --- |
| IBP485 | Fw inuAp-uso1 E6K FUS | GCGGTCAGCGACATCACCAAAATGTTTCGAATCCTCAAATCACAG |
| IBP629 | Fw N-helixΔuso1 | GCGGTCAGCGACATCACCAAAATGACGGCGACGGACACGATC |
| IBP628 | Rv uso1 full-riboB FUS | CCAGACTCCTGAACGGCCTCTCACCCATGCTCCTCGTCAC |

**(3) *Aspergillus fumigatus riboB* gene as selection marker**

| IBP230 | Fw riboB Af | GAGGCCGTTCAGGAGTCTGG |
| --- | --- | --- |
| IBP231 | Rv riboB Af | CAGAACGTTTGCGCTGCAGAAC |

**(4) *inuA* gene 3’-flanking region**

| IBP279 | Fw Fus-inuA3-riboB | GTTCTGCAGCGCAAACGTTCTGGGATCTAGCTAGATGTTTTGTTG |
| --- | --- | --- |
| SF3 | downstream inuA | CAGCAGTCAAGCAATACCAAGC |

**Expression of GFP-Bos1 fusion driven by its own promoter from the *wA* locus**

**Gene replacement cassette assembled by 6-way fusion PCR:**

**(1) upstream *wA***

| MP411 | Fw upstream *wA* | CGTCTATTGTAGAGCCTCCATGC |
| --- | --- | --- |
| MP412 | Rv upstream *wA* | GATCAGGAGAAGGAGAGTCAAG |

**(2) upstream *bos1(*AN11900) + 5’UTR**

| IBP620 | Fw ups wA-bos1p+5'UTR FUS | CTTGACTCTCCTTCTCCTGATCTTCAAATGATCCGGCACTCGATTG |
| --- | --- | --- |
| IBP621 | Rv bos1+5'UTR-GFP FUS | GAAAAGTTCTTCTCCTTTACTCATCTTCTGGTTTGTTCTCAGCAAGTTC |

**(3) GFP + GA5x linker**

| IBP622 | Fw GFP | ATGAGTAAAGGAGAAGAACTTTTCAC |
| --- | --- | --- |
| IBP623 | Rv GFP-GA5x | GGCTCCAGCGCCTGCACCAGCACCTTTGTATAGTTCATCCATGCCATG |

**(4) *bos1 cDNA +* 3’UTR**

| IBP626 | Fw GA5x-bos1 cDNA | GGTGCAGGCGCTGGAGCCGGTGCCAATTCACTGTTCAACTCCGCTC |
| --- | --- | --- |
| IBP627 | Rv bos1 3'UTR-riboB FUS | CCAGACTCCTGAACGGCCTCCGTTCCGGTCACATGGCAAC |

**(5) *Aspergillus fumigatus riboB* gene as selection marker**

| IBP230 | Fw riboB Af | GAGGCCGTTCAGGAGTCTGG |
| --- | --- | --- |
| IBP452 | Rv riboBAf-dw wA | CGGGAAAGTAAAACCTTATGAGCCAGAACGTTTGCGCTGCAGAAC |

**(6) downstream *wA***

| MP415 | Fw downstream *wA* | GCTCATAAGGTTTTACTTTCCCG |
| --- | --- | --- |
| MP416 | Rv downstream *wA* | CACCTGACTCGCATTGGAGAAC |
