# Supplementary material for: The Uso1 globular head interacts with SNAREs to maintain viability even in the absence of the coiled-coil domain
Source: eLife. 2023 May 30;12:e85079. doi: 10.7554/eLife.85079 (PMC10275640; doi:10.7554/eLife.85079)
Supplement: MDAR checklist [file elife-85079-mdarchecklist1.docx]

**Materials Design Analysis Reporting (MDAR)**

**Checklist for Authors**

The [MDAR framework](https://osf.io/xfpn4/) establishes a minimum set of requirements in transparent reporting mainly applicable to studies in the life sciences.

*eLife* asks authors to **provide detailed information within their article** to facilitate the interpretation and replication of their work. Authors can also upload supporting materials to comply with relevant reporting guidelines for health-related research (see [EQUATOR Network](http://www.equator-network.org/%20)), life science research (see the [BioSharing Information Resource](http://biosharing.org/)), or animal research (see the [ARRIVE Guidelines](http://www.plosbiology.org/article/info:doi/10.1371/journal.pbio.1000412) and the [STRANGE Framework](https://doi.org/10.1038/d41586-020-01751-5); for details, see *eLife*’s [Journal Policies](https://reviewer.elifesciences.org/author-guide/journal-policies)). Where applicable, authors should refer to any relevant reporting standards materials in this form.

For all that apply, please note **where in the article** the information is provided. Please note that we also collect information about data availability and ethics in the submission form.

**Materials:**

| **Newly created materials** | **Indicate where provided: section/figure legend** | **N/A** |
| --- | --- | --- |
| The manuscript includes a dedicated "materials availability statement" providing transparent disclosure about availability of newly created materials including details on how materials can be accessed and describing any restrictions on access. | This specifically refers to a fungal strains and DNA constructs that will be supplied to others upon reasonable request. Both strains and primers required to obtain the DNA constructs are listed as attachments in the form of supplemental tables. With the detailed list of primers provided, and the schematic description of the molecules included in Methods, anyone proficient molecular cloning should be capable of generating any of their construct used in this work. A dedicated Materials availability statement is included within Methods.s |  |
|  |  |  |
| **Antibodies** | **Indicate where provided: section/figure legend** | **N/A** |
| For commercial reagents, provide supplier name, catalogue number and [RRID](https://scicrunch.org/resources), if available. | The supplier and catalogue numbers for all commercial antibodies used in this study are listed in a table included in Materials & Methods, section “Antibodies for western blotting”. Antiserum against the Uso1 GHD is available upon request . |  |
|  |  |  |
| **DNA and RNA sequences** | **Indicate where provided: section/figure legend** | **N/A** |
| Short novel DNA or RNA including primers, probes: Sequences should be included or deposited in a public repository. | Primers are listed in supplementary table III. None is novel and worth to be included in a repository. |  |
|  |  |  |
| **Cell materials** | **Indicate where provided: section/figure legend** | **N/A** |
| Cell lines: Provide species information, strain. Provide accession number in repository OR supplier name, catalog number, clone number, OR RRID. | Does not apply | **X** |
| Primary cultures: Provide species, strain, sex of origin, genetic modification status. | Does not apply | **X** |
|  |  |  |
| **Experimental animals** | **Indicate where provided: section/figure legend** | **N/A** |
| Laboratory animals or Model organisms: Provide species, strain, sex, age, genetic modification status. Provide accession number in repository OR supplier name, catalog number, clone number, OR RRID. | Does not apply | **X** |
| Animal observed in or captured from the field: Provide species, sex, and age where possible. | Does not apply | **X** |
|  |  |  |
| **Plants and microbes** | **Indicate where provided: section/figure legend** | **N/A** |
| Plants: provide species and strain, ecotype and cultivar where relevant, unique accession number if available, and source (including location for collected wild specimens). | Does not apply | **X** |
| Microbes: provide species and strain, unique accession number if available, and source. | *Aspergillus nidulans* strains used in this study are listed in supplementary table II. They are all deposited in the Madrid collection and available upon reasonable request |  |
|  |  |  |
| **Human research participants** | **Indicate where provided: section/figure legend) or state if these demographics were not collected** | **N/A** |
| If collected and within the bounds of privacy constraints report on age, sex, gender and ethnicity for all study participants. | Does not apply | **X** |

**Design:**

| **Study protocol** | **Indicate where provided: section/figure legend** | **N/A** |
| --- | --- | --- |
| If the study protocol has been pre-registered, provide DOI. For clinical trials, provide the trial registration number OR cite DOI. |  | **X** |
|  |  |  |
| **Laboratory protocol** | **Indicate where provided: section/figure legend** | **N/A** |
| Provide DOI OR other citation details if detailed step-by-step protocols are available. | Does not apply | X |
|  |  |  |
| **Experimental study design (statistics details) *** | | |
| **For in vivo studies: State whether and how the following have been done** | **Indicate where provided: section/figure legend. If it could have been done, but was not, write “not done”** | **N/A** |
| Sample size determination | Does not apply | X |
| Randomisation | Does not apply | **X** |
| Blinding | Does not apply | X |
| Inclusion/exclusion criteria | Does not apply |  |
|  |  |  |
| **Sample definition and in-laboratory replication** | **Indicate where provided: section/figure legend** | **N/A** |
| State number of times the experiment was replicated in the laboratory. | For microscopy experiments, the number of hyphal cells observed or events included in the statistical analysis is indicated on the graph and/or in the text. Check results/section “Uso1-GFP localizes to the early Golgi in a Rab1-dependent manner” see also Figures 4, 5 and 6. Results/section “Uso1 delocalization and partial Golgi disorganization after RAB1 impairment rescued by E6K G540S” check also figure 7.  S-tag co-precipitation experiments (Results, section titled “Uso1 is an associate of the early Golgi SNARE machinery, with the double substitution E6K/G540S increasing this association”) Figure 10, foot note says “Each panel is a representative experiment of three technical replicates”.  Pull down assays with recombinant proteins (Results/ “Golgi SNAREs bind directly to the Uso1 GHD; effects of Uso1 E6K/G540S” section) the number of independent technical replicates is listed on Figure 11, panels B, E and H.  Materials & Methods, section Dynamic Light Scattering, DLS indicates that “At least 18 replicates from each dataset were averaged to yield the mean and standard deviation of the autocorrelation curve values for a given time” |  |
| Define whether data describe technical or biological replicates. | Number of hyphal cells (biological replicates) for microscopy experiments. Results/section “Uso1-GFP localizes to the early Golgi in a Rab1-dependent manner” also Figures 4, 5 and 6. Results/section “Uso1 delocalization and partial Golgi disorganization after RAB1 impairment rescued by E6K G540S” see also figure 7.  S-tag co-precipitation experiments (Results, section titled “Uso1 is an associate of the early Golgi SNARE machinery, with the double 516 substitution E6K/G540S increasing this association”) Figure 10, foot note says “Each panel is a representative experiment of three technical replicates”.  For pull down assays with recombinant proteins (Results/ “Golgi SNAREs bind directly to the Uso1 GHD; effects of Uso1 E6K/G540S” section) the number of independent technical replicates is listed on Figure 11, panels B, E and F. |  |
|  |  |  |
| **Ethics** | **Indicate where provided: section/submission form** | **N/A** |
| Studies involving human participants: State details of authority granting ethics approval (IRB or equivalent committee(s), provide reference number for approval. |  | X |
| Studies involving experimental animals: State details of authority granting ethics approval (IRB or equivalent committee(s), provide reference number for approval. |  | X |
| Studies involving specimen and field samples: State if relevant permits obtained, provide details of authority approving study; if none were required, explain why. |  | X |
|  |  |  |
| **Dual Use Research of Concern (DURC)** | **Indicate where provided: section/submission form** | **N/A** |
| If study is subject to dual use research of concern regulations, state the authority granting approval and reference number for the regulatory approval. |  | X |

**Analysis:**

| **Attrition** | **Indicate where provided: section/figure legend** | **N/A** |
| --- | --- | --- |
| Describe whether exclusion criteria were pre-established. Report if sample or data points were omitted from analysis. If yes, report if this was due to attrition or intentional exclusion and provide justification. | The strategy used to calculate the Half-life of Uso1 in cisternae, and how representative events were chosen is detailed in Results, section “The punctate pattern of localization of USO1-GFP is dependent on RAB1”. |  |
|  |  |  |
| **Statistics** | **Indicate where provided: section/figure legend** | **N/A** |
| Describe statistical tests used and justify choice of tests. | For multiple comparisons of number of Uso1 puncta among different conditions, statistical tests are described in Figure 5, panel D footnote. In the case of Gea1 cisternae, as depicted in Figure 7C, a description of the statistical analysis performed can be found at panel D footnote  Pull down assays with recombinant proteins. Statistical test used to compare levels of wild-type and mutant prey recovery are indicated in Figure 11, panels B, E and H footnote. |  |
|  |  |  |
| **Data availability** | **Indicate where provided: section/submission form** | **N/A** |
| For newly created and reused datasets, the manuscript includes a data availability statement that provides details for access (or notes restrictions on access). | All data generated or analysed during this study are included in the manuscript and supporting files; Source Data files have been provided for Figures 8 through 11. Supplemental tables II and III (strains and primers) ensure the reproducibility of the experiments. |  |
| When newly created datasets are publicly available, provide accession number in repository OR DOI and licensing details where available. |  | X |
| If reused data is publicly available provide accession number in repository OR DOI, OR URL, OR citation. |  | X |
|  |  |  |
| **Code availability** | **Indicate where provided: section/figure legend** | **N/A** |
| For any computer code/software/mathematical algorithms essential for replicating the main findings of the study, whether newly generated or re-used, the manuscript includes a data availability statement that provides details for access or notes restrictions. | AlphaFold2 (Jumper et al., 2021) predictions were run using versions of the program installed locally on ColabFold (Mirdita et al., 2022) with the AlphaFold2_advanced.ipynb notebook and the MMseqs2 MSA option (materials and Methods) |  |
| Where newly generated code is publicly available, provide accession number in repository, OR DOI OR URL and licensing details where available. State any restrictions on code availability or accessibility. |  | X |
| If reused code is publicly available provide accession number in repository OR DOI OR URL, OR citation. | <https://github.com/sokrypton/ColabFold/blob/main/beta/AlphaFold2_advanced.ipynb> |  |

**Reporting:**

The MDAR framework recommends adoption of discipline-specific guidelines, established and endorsed through community initiatives.

| **Adherence to community standards** | **Indicate where provided: section/figure legend** | **N/A** |
| --- | --- | --- |
| State if relevant guidelines (e.g., ICMJE, MIBBI, ARRIVE, STRANGE) have been followed, and whether a checklist (e.g., CONSORT, PRISMA, ARRIVE) is provided with the manuscript. |  | **X** |

* We provide the following guidance regarding transparent reporting and statistics; we also refer authors to [Ten common statistical mistakes to watch out for when writing or reviewing a manuscript](https://doi.org/10.7554/eLife.48175).

**Sample-size estimation**

- You should state whether an appropriate sample size was computed when the study was being designed
- You should state the statistical method of sample size computation and any required assumptions
- If no explicit power analysis was used, you should describe how you decided what sample (replicate) size (number) to use

**Replicates**

- You should report how often each experiment was performed
- You should include a definition of biological versus technical replication
- The data obtained should be provided and sufficient information should be provided to indicate the number of independent biological and/or technical replicates
- If you encountered any outliers, you should describe how these were handled
- Criteria for exclusion/inclusion of data should be clearly stated
- High-throughput sequence data should be uploaded before submission, with a private link for reviewers provided (these are available from both GEO and ArrayExpress)

**Statistical reporting**

- Statistical analysis methods should be described and justified
- Raw data should be presented in figures whenever informative to do so (typically when N per group is less than 10)
- For each experiment, you should identify the statistical tests used, exact values of N, definitions of center, methods of multiple test correction, and dispersion and precision measures (e.g., mean, median, SD, SEM, confidence intervals; and, for the major substantive results, a measure of effect size (e.g., Pearson's r, Cohen's d)
- Report exact p-values wherever possible alongside the summary statistics and 95% confidence intervals. These should be reported for all key questions and not only when the p-value is less than 0.05.

**Group allocation**

- Indicate how samples were allocated into experimental groups (in the case of clinical studies, please specify allocation to treatment method); if randomization was used, please also state if restricted randomization was applied
- Indicate if masking was used during group allocation, data collection and/or data analysis
